# A Re-Evaluation of the Chasmosaurine Ceratopsid Genus *Chasmosaurus* (Dinosauria: Ornithischia) from the Upper Cretaceous (Campanian) Dinosaur Park Formation of Western Canada

**DOI:** 10.1371/journal.pone.0145805

**Published:** 2016-01-04

**Authors:** James A. Campbell, Michael J. Ryan, Robert B. Holmes, Claudia J. Schröder-Adams

**Affiliations:** 1 Department of Earth Sciences, Carleton University, Ottawa, Ontario, Canada; 2 Department of Biological Sciences, University of Calgary, Calgary, Alberta, Canada; 3 Department of Vertebrate Paleontology, Cleveland Museum of Natural History, Cleveland, Ohio, United States of America; 4 Department of Biological Sciences, University of Alberta, Edmonton, Alberta, Canada; NYIT College of Osteopathic Medicine, UNITED STATES

## Abstract

**Background:**

The chasmosaurine ceratopsid *Chasmosaurus* is known from the Upper Cretaceous (Campanian) Dinosaur Park Formation of southern Alberta and Saskatchewan. Two valid species, *Chasmosaurus belli* and *C*. *russelli*, have been diagnosed by differences in cranial ornamentation. Their validity has been supported, in part, by the reported stratigraphic segregation of chasmosaurines in the Dinosaur Park Formation, with *C*. *belli* and *C*. *russelli* occurring in discrete, successive zones within the formation.

**Results/Conclusions:**

An analysis of every potentially taxonomically informative chasmosaurine specimen from the Dinosaur Park Formation indicates that *C*. *belli* and *C*. *russelli* have indistinguishable ontogenetic histories and overlapping stratigraphic intervals. Neither taxon exhibits autapomorphies, nor a unique set of apomorphies, but they can be separated and diagnosed by a single phylogenetically informative character—the embayment angle formed by the posterior parietal bars relative to the parietal midline. Although relatively deeply embayed specimens (*C*. *russelli*) generally have relatively longer postorbital horncores than specimens with more shallow embayments (*C*. *belli*), neither this horncore character nor epiparietal morphology can be used to consistently distinguish every specimen of *C*. *belli* from *C*. *russelli*.

**Status of *Kosmoceratops* in the Dinosaur Park Formation:**

*Kosmoceratops* is purportedly represented in the Dinosaur Park Formation by a specimen previously referred to *Chasmosaurus*. The reassignment of this specimen to *Kosmoceratops* is unsupported here, as it is based on features that are either influenced by taphonomy or within the realm of individual variation for *Chasmosaurus*. Therefore, we conclude that *Kosmoceratops* is not present in the Dinosaur Park Formation, but is instead restricted to southern Laramidia, as originally posited.

## Introduction

**Fig 1:**
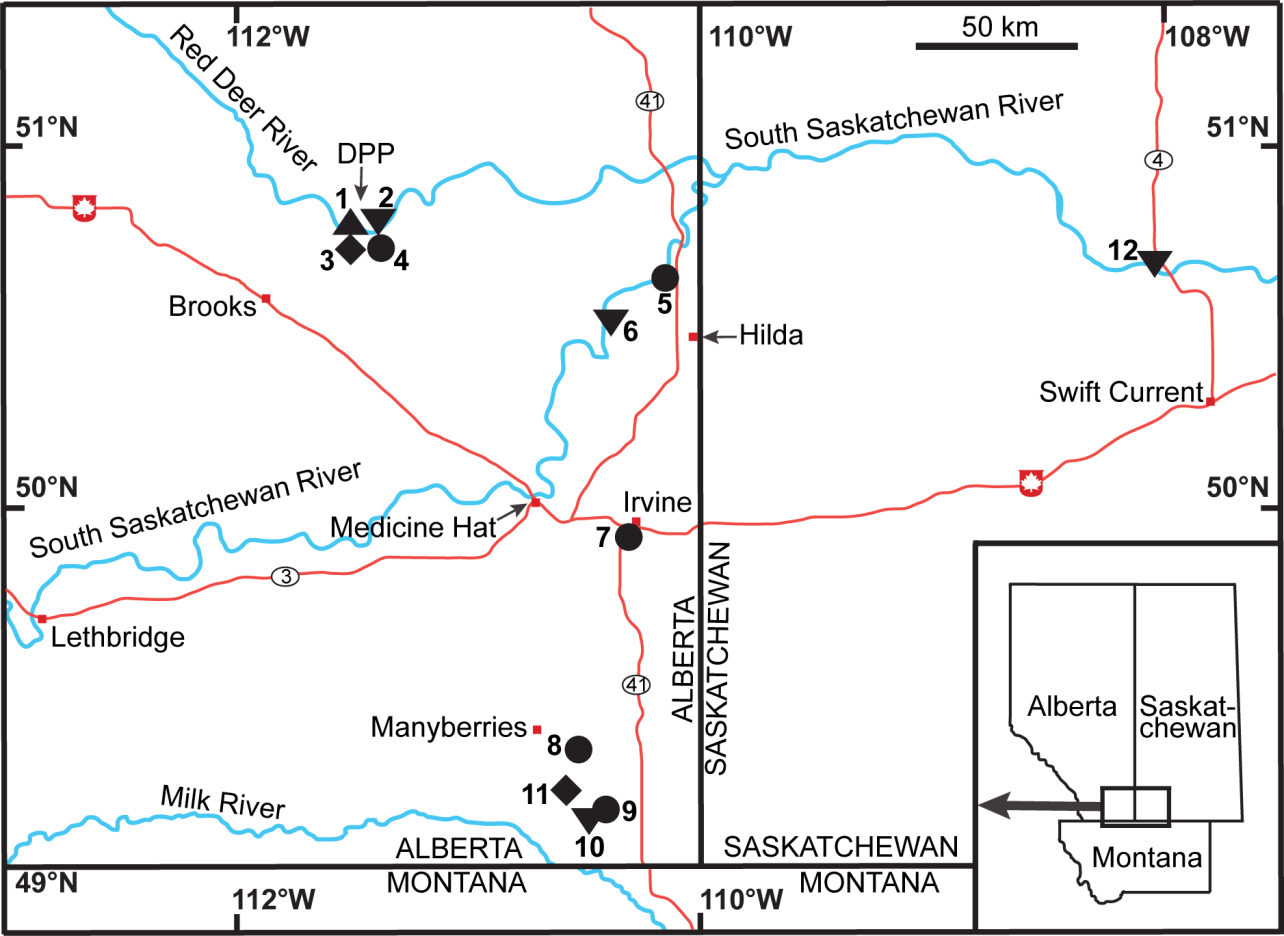
Regional map of *Chasmosaurus belli*, *Chasmosaurus russelli*, *Chasmosaurus* sp., and *Vagaceratops* specimens. (1) AMNH 5402, CMN 0491, CMN 2245, NHMUK R4948, ROM 843, and YPM 2016; (2) AMNH 5656, CMN 2280, and TMP 1999.055.0292; (3) AMNH 5401, CMN 1254, CMN 8801, CMN 34829, CMN 34832, ROM 839, TMP 1979.011.0147, TMP 1981.019.0175, TMP 1993.082.0001, and UALVP 40; (4) TMP 1987.045.0001; (5) TMP 2009.034.0009; (6) TMP 1997.132.0002; (7) CMN 41357; (8) TMP 2011.053.0046; (9) TMP 1998.102.0008; (10) CMN 8800; (11) CMN 8802; and (12) CMN 8803. *Chasmosaurus belli* (triangle), *C*. *russelli* (inverted triangles), *Chasmosaurus* sp. (diamonds), and *Vagaceratops* (circles); DPP = Dinosaur Provincial Park. [planned for page width].

*Chasmosaurus* is a chasmosaurine ceratopsid known from the Upper Cretaceous (Campanian) Dinosaur Park Formation (DPF) of southern Alberta and Saskatchewan (Fig 1). The first specimens of *Chasmosaurus* (CMN 0491, CMN 1254) were collected by Lawrence Lambe in 1897 from what is now Dinosaur Provincial Park (DPP). Originally referred to *Monoclonius* [1], these specimens were subsequently reassigned to the new genus *Chasmosaurus* [2]. Since that time, dozens of partial-to-complete skulls have been collected from the DPF of DPP, southeast Alberta, and Saskatchewan.

*Chasmosaurus* has a long convoluted taxonomic history, with seven species having been described. A detailed reappraisal of five of these species (*C*. *belli*, *C*. *brevirostris*, *C*. *canadensis*, *C*. *kaiseni*, and *C*. *russelli*) was conducted by Godfrey & Holmes [3], who implicitly tested the null hypothesis that only one valid species exists. Although admitting that considerable gradational variation existed among *Chasmosaurus* specimens, they retained *C*. *belli* and *C*. *russelli* as distinct taxa, and used the purported stratigraphic segregation of specimens assigned to these taxa in the DPF (i.e., *C*. *belli* temporally replacing *C*. *russelli*) to support their conclusions. The two remaining species, *Chasmosaurus mariscalensis* from the Aguja Formation of Texas, and *Chasmosaurus irvinensis* from the DPF of Alberta, have been reassigned to the new genera *Agujaceratops* [4] and *Vagaceratops* [5], respectively. Longrich [6] subsequently erected the new taxon, *Mojoceratops perifania*, from specimens previously referred to *Chasmosaurus*. Maidment & Barrett [7] supported and refined the conclusions of Godfrey & Holmes [3], and argued that *M*. *perifania* is a junior subjective synonym of *C*. *russelli* based on their inability to confirm any of the autapomorphies of *Mojoceratops* identified by Longrich [6]. Longrich [8] later reassigned a previously referred *Chasmosaurus* skull (CMN 8801) to *Kosmoceratops* sp. Most recently, Konishi [9] proposed that *C*. *canadensis* may be a valid species of *Chasmosaurus*, characterized by elongate postorbital horncores (*sensu* [10]), but did not formally resurrect this taxon.

**Fig 2:**
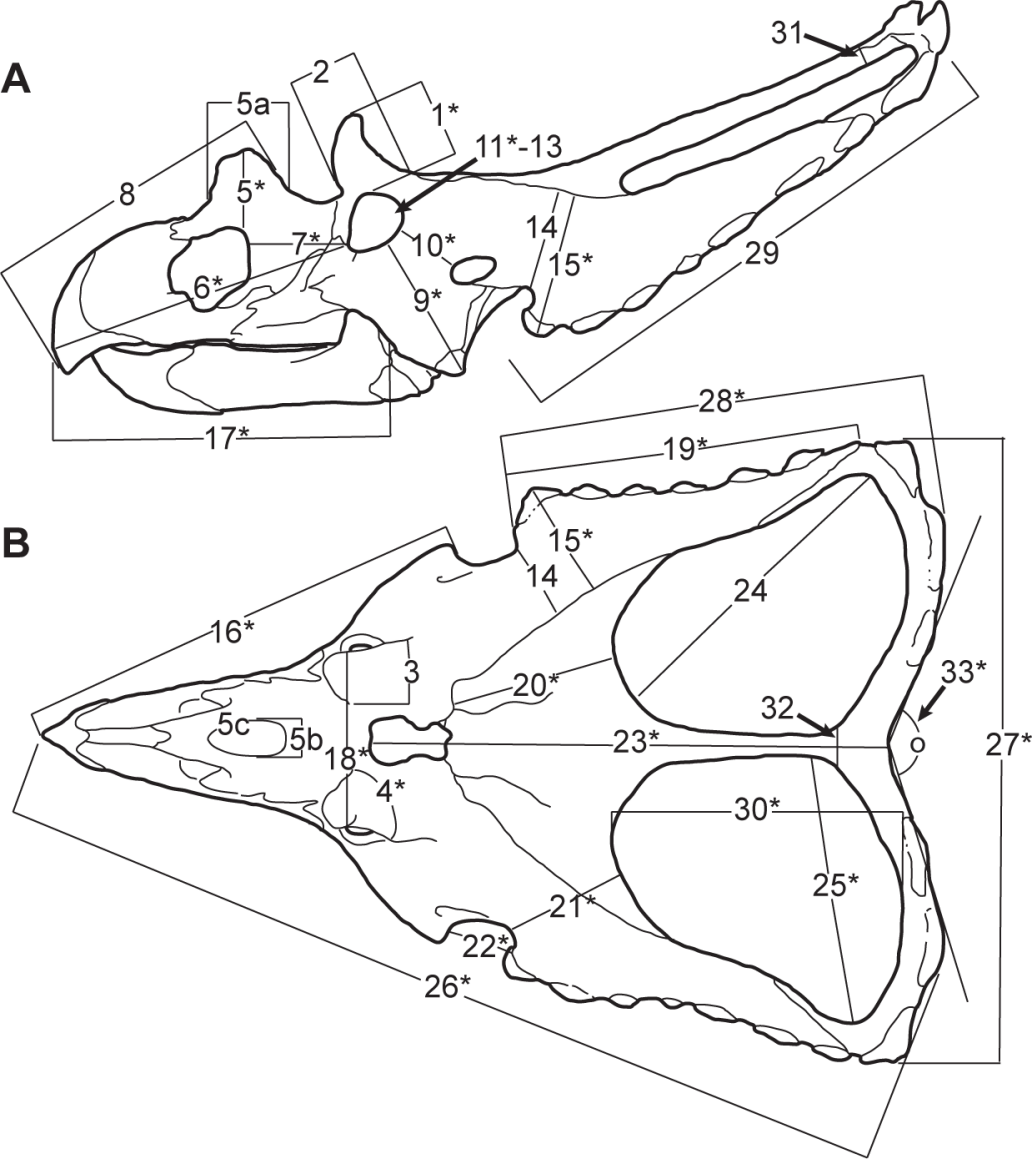
Parameters of cranial measurements used in this study. (A) lateral view, and (B) dorsal view (modified from [3]: Fig 5). Asterisks denote parameters used in principal component analysis. [planned for column width].

The distinction between *C*. *belli* and *C*. *russelli* is obscured by a significant degree of morphological overlap in purportedly diagnostic cranial characters, in particular the morphology and ornamentation of the posterior margin of the parietal (Fig 2). Lehman [11] argued that *C*. *belli* and *C*. *russelli* are end-members of a gradational morphological spectrum resulting from individual, ontogenetic, and/or sexual dimorphism, but did not quantify this variation or formally synonymise these taxa. He also questioned the existence of geographic or stratigraphic separation of the two species, but did not provide specific support for this argument.

This study revisits the null hypothesis that there is only one species of *Chasmosaurus*. This was tested by evaluating the stratigraphic, phylogenetic, morphometric, and ontogenetic relationships of all DPF chasmosaurine specimens previously referred to *Chasmosaurus*. These analyses are facilitated by detailed specimen stratigraphic work (e.g., [12]; this study) and descriptions of recently-prepared specimens (e.g., [7]) that have been conducted in the past 20 years. While we test previous claims about the taxonomic composition of *Chasmosaurus*, we also remain open to the possibility that other, previously unrecognized species may be present.

### Geology and biostratigraphy of the Dinosaur Park Formation

The DPF is the uppermost unit of the terrestrial Belly River Group, which was deposited along the western margin of the Western Interior Seaway [13]. This group also includes, in ascending stratigraphic order, the Foremost and Oldman formations. The Oldman and Dinosaur Park formations both have a wedge-shaped geometry, due to differing sediment sources along the rising Cordillera to the west, resulting in a regionally-diachronous contact between them that becomes younger to the south and west [14]. Sediments of the DPF record an overall transgression, transitioning from a sandy to muddy to coaly interval (the Lethbridge Coal Zone (LCZ)). The DPF is overlain by the marine Bearpaw Formation [13].

In Dinosaur Provincial Park (DPP), where the entirely exposed DPF is 70 m thick, bentonites collected from the top of the Oldman Formation (5.5 m below the DPF), the middle of the DPF (36.0 m above the Oldman Formation), and near the base of the LCZ (61.5 m above the Oldman Formation) yielded ^40^Ar/^39^Ar dates of 77.0±0.5 Ma, 76.4±0.4 Ma, and 76.1±0.5 Ma, respectively (D. Eberth, pers. comm.). Assuming a constant sedimentation rate in DPP, the lower (0.0–36.0 m) and upper (36.0–61.5 m) halves of the DPF experienced rates of approximately 6.9 cm/1000 years and 8.5 cm/1000 years, respectively.

The DPF also currently has three recognized distinct faunal zones, each of which is characterized by a unique assemblage of centrosaurine and lambeosaurine taxa [15, 16]. These Dinosaur Park faunal zones (DPFZs) are, in ascending stratigraphic order: *Centrosaurus*-*Corythosaurus* Zone (DPFZ 1), *Styracosaurus*-*Lambeosaurus lambei* Zone (DPFZ 2), and *Lambeosaurus magnicristatus*-pachyrhinosaur Zone (DPFZ 3). *Chasmosaurus russelli*, *C*. *belli*, and *V*. *irvinensis* are also thought to be constrained to these three zones, respectively [15, 16].

### Institutional abbreviations

**AMNH**, American Museum of Natural History, New York; **CMN**, Canadian Museum of Nature, Ottawa; **NHMUK**, Natural History Museum of the United Kingdom, London; **ROM**, Royal Ontario Museum, Toronto; **TMP**, Royal Tyrrell Museum of Palaeontology, Drumheller; **UALVP**, University of Alberta Laboratory for Vertebrate Palaeontology; **YPM**, Yale Peabody Museum, Yale University, New Haven.

### Anatomical abbreviations

**Es**, episquamosal; **es lc**, episquamosal locus; **fpf**, frontoparietal fontanelle; **fr**, frontal; **m**, maxilla; **n**, nasal; **nhc**, nasal horncore; **ns**, nares; **n sp**, narial spine; **n st**, narial strut; **p**, epiparietal; **pau**, parietal undulation (possible epiparietal locus); **p lc**, epiparietal locus; **p-es**, epiparietosquamosal; **pm**, premaxilla; **pm-n dc**, premaxilla-nasal dorsal contact; **pm ome**, expanded oral margin of premaxilla; **pm pvpr**, premaxilla posteroventral process; **pm s**, premaxillary septum; **pm s vr**, ventral recess on premaxillary septum; **po**, postorbital; **r**, rostral; **r dpr**, rostral dorsal process; **r vpr**, ventral process of rostral; **sbor f**, subordinate fossa; **sf**, septal fossa; **sf acc st**, septal fossa accessory strut; **sfl**, septal flange; **tpr**, triangular process; **tpr rc**, triangular process recess.

## Materials and Methods

All specimens previously referred to *Chasmosaurus* and *Vagaceratops* were studied, measured, and photographed first hand, except for NHMUK R4948, whose relevant information was obtained from Maidment & Barrett [7]. Provenience data for all chasmosaurine specimens collected from the Dinosaur Park Formation (DPF) were obtained from Currie & Russell [12], the CMN (M. Currie, pers. comm.) and TMP collections database [17], and D. Eberth (pers. comm.). Measurements for AMNH 5401 and AMNH 5402 were taken from Godfrey & Holmes [3], as these specimens are inaccessibly displayed. These data, as well as associated historical taxonomic referrals, are given in S1 File. This DPF chasmosaurine dataset also includes the specimens referred to *Mercuriceratops gemini* (UALVP 54559; [18]) and *Pentaceratops aquilonius* (CMN 9813 and CMN 9814; [8]), for the sake of completeness. The expression “DPF chasmosaurine specimens”, as used in this paper, excludes the above three specimens.

The specimens were subjected to stratigraphic, phylogenetic, morphometric, and ontogenetic analyses to determine if any of these methodologies could be used to cluster the specimens into more than one discrete operative taxonomic unit that could be diagnosed as a species within the clade *Chasmosaurus*. Specimens previously referred to *Vagaceratops irvinensis* were also included in the analyses as it has previously been recovered as a species of *Chasmosaurus* in the DPF [19].

We define a fossil species using the morphological species concept instead of the biological species concept, as in the case of this study, it cannot be determined whether distinct morphs (i.e., putative species) were reproductively isolated or not [20].

### Stratigraphic analysis

*Chasmosaurus* and *Vagaceratops* specimens (n = 15) with verified locality data were arranged into a stratigraphic sequence to test for the discrete partitioning of *C*. *belli* and *C*. *russelli*.

The stratigraphic sequence of specimens within the DPF of Dinosaur Provincial Park (DPP) was determined by plotting their geographic locations onto the DPP structural contour map of Eberth [13]. The contours of this map mark the approximate elevation (metres above sea level) of the regional disconformity within this region; the elevation of this disconformity varies by 30 m between the northwest and southeast corners of the DPP region [13]. By subtracting a specimen’s elevation above sea level from the contact’s elevation above sea level in a given locality on the map, that specimen’s elevation above the contact could be determined. For specimens collected to the south and west of DPP where the Oldman-Dinosaur Park formational contact is younger [14], their stratigraphic positions were determined relative to the base of the Lethbridge Coal Zone. Error bars for each specimen were taken at ± 5 m following the methods of Ryan [21], which take into account the potential problem of specimens preserved in palaeochannels.

### Phylogenetic analysis

A phylogenetic analysis was performed using a matrix modified from Campbell ([22]; see S2 File for details). This matrix consists of 155 characters, of which 133 are cranial and 22 are postcranial, including three new characters (153–155) of the P4 and P5 epiparietals.

The analysis was performed using the tree bisection reconnection search algorithm, with 10 trees saved per replication (n = 1000), and with *Leptoceratops* assigned to the outgroup. All characters were treated as unordered. We started with Wagner trees, with a random seed of one. The analysis (strict and 50% majority rule consensus trees) and corresponding tree statistics (Bootstrap values and Bremer support) were conducted in TNT 1.1 [23, 24]. Bootstrap values were calculated using 1000 replicates. Consistency (CI) and retention (RI) indices were obtained in PAUP 4b10 [25] using the matrix assembled in Mesquite v.2.75 [26]. The matrix is provided in TNT and NEXUS formats in S3 and S4 Files, respectively.

Each taxon included in this analysis has one set of coded characters, except for *Chasmosaurus* and *Vagaceratops*, whose referred specimens were coded separately; i.e., no *a priori* assumptions were made about their specific identities. Some taxa and specimens included in the analysis are especially fragmentary and/or lack diagnostic features, and were excluded in subsequent iterations in order to improve the resolution within the consensus tree. These exclusions were conducted *a posteriori*, and the consensus tree recalculated, in order to avoid excluding potentially informative morphological data from the analysis.

We note that ontogenetically variable characters, while useful in growth studies, can be problematic in phylogenetic analyses, as some have states that vary phylogenetically as well. For example, a relatively immature ceratopsid specimen lacking features expressed later in life (e.g., epiparietals) could plot out as a less derived species on a phylogenetic tree than would a more mature representative of that taxon [27]. Therefore, character codings for a given taxon should be derived from relatively mature specimens. The ontogenetic nature of some characters does not justify their removal from our phylogenetic analysis; rather, these characters should be included, but their ontogenetic nature should be noted (see S5 File).

### Morphometric analysis

A principal component analysis (PCA) of *Chasmosaurus* specimens (n = 16) was conducted to determine their morphometric relationships. We then tested the morphometric null hypothesis that specimens assigned *a priori* to *C*. *belli* and *C*. *russelli* do not form statistically different clusters in morphospace. If this null hypothesis is rejected, *C*. *belli* and *C*. *russelli* are morphometrically distinguishable, supporting their validities as distinct taxa. *Vagaceratops irvinensis* specimens (n = 2) were also included due to their previous referral to *Chasmosaurus*.

**Fig 3:**
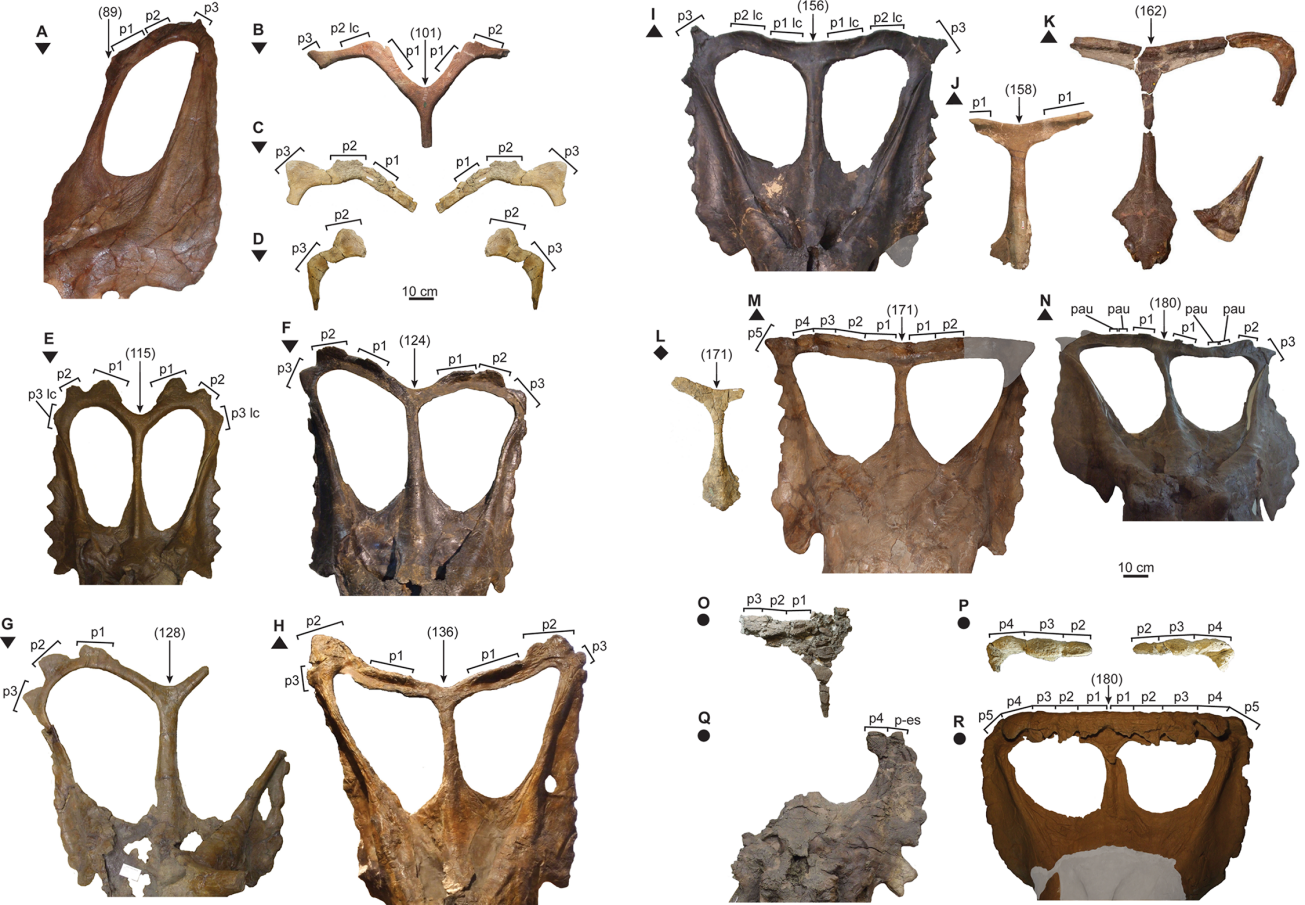
*Chasmosaurus* and *Vagaceratops* frills. *Chasmosaurus*: (A) CMN 8800 (*Chasmosaurus russelli* holotype), (B) CMN 8803, (C) TMP 1999.055.0292, (D) TMP 1997.132.0002, (E) AMNH 5656, (F) CMN 2280, (G) TMP 1983.025.0001 (*Mojoceratops perifania* holotype), (H) ROM 843 (cast), (I) CMN 2245, (J) CMN 0491 (*Chasmosaurus belli* holotype), (K) NHMUK R4948 ([7]: Fig 9), (L) TMP 2008.012.0001, (M) YPM 2016, and (N) AMNH 5402. *Vagaceratops*: (O) TMP 2009.034.0009, (P) TMP 1998.102.0008, (Q) TMP 1987.045.0001, and (R) CMN 41357 (*V*. *irvinensis* holotype; cast). Rectangular brackets delimit size of epiossifications; numbers in brackets above frill denote posterior embayment angle. The posterior margin ‘K’ and ‘L’ are not labelled, as this region is either too difficult to interpret (‘K’) or insufficiently preserved (‘L’). In both ‘C’ and ‘D’, the real right fragment is reflected on the left to help reconstruct the frill. *Chasmosaurus belli* (triangle), *C*. *russelli* (inverted triangles), *Chasmosaurus* sp. (diamond), and *V*. *irvinensis* (circles). Plaster reconstruction = grey. [two pages, each planned for page width].

The PCA was conducted using the R statistical programming language software [28]. Of the 36 cranial parameters recorded in this study (Fig 2, S6 File), 12 were appropriate for testing because they capture the overall shape of the skull while minimizing redundant measurements. The variance-covariance matrix was used, as all 12 parameters (1, 4, 5, 16, 18, 19, 22, 27, 28, 30, 31, and 32) are measured in the same unit (mm); these parameters were log-transformed prior to conducting the PCA. The embayment of the posterior bar (defined by the angle between two lines that originate at the midpoint of the posterior parietal bar, and lay tangent to the posterior margin of the posterior bar excluding the epiparietals; Fig 2, parameter 33) is one of the most variable features among *Chasmosaurus* skulls (Fig 3). This parameter was included in an earlier version of this PCA (using the correlation matrix instead), which yielded a significant difference between *C*. *belli* and *C*. *russelli*. This parameter was excluded from the PCA in this study to determine whether these two species could be distinguished by other parameters.

A Bayesian PCA (BPCA; [29]) was conducted, using the “bpca” function in the R package “pcaMethods” [30]. BPCA has been shown to be the most reliable missing data estimator method for morphometric analyses, in cases where the amount of missing data is less than 35% of the dataset [31]; the amount of missing data in our study is 27%. The PCA described above (PCA 1) was then run on this estimated, complete dataset. The scores of each principal component (PC) axis in PCA 1 were then plotted against the size-related parameter 17 (Fig 2; rostral to back of maxillary toothrow). This was done to determine whether any PCs are strongly correlated with skull size and, hence, ontogeny.

An attempt was also made to reduce the confounding effects of variable skull sizes as a result of ontogenetic differences. This was done by plotting each of the 12 complete, estimated parameters used in PCA 1 against parameter 17, and assigning a line-of-best-fit (ordinary least squares). The parameter residuals obtained from the line-of-best-fit were then included in a second PCA (PCA 2). Statistical comparisons between morphometric clusters of specimens corresponding to *C*. *belli* and *C*. *russelli* in both PCA 1 and PCA 2 were made using PC scores, and performed using PAST 3.01 [32]. An analysis of variance (ANOVA) was performed on clusters that showed separation along a given PC axis. A non-parametric multivariate ANOVA (NPMANOVA) was also performed on taxonomic clusters that showed separation along pairs of PC axes, measured by permutation across groups using 10,000 replicates, and based on the Mahalanobis distance measure [33].

### Ontogenetic analysis

Ontogenetically variable characters are useful for testing the diversity of taxa in a fossil ecosystem, and their use in other dinosaur growth studies have resulted in: 1) the establishment of age classes within a given taxon, e.g., *Albertosaurus sarcophagus* [34] and *Agujaceratops mariscalensis* [35]; or 2) the invalidation of taxa originally based on immature material, e.g., referring the tyrannosaurs *Stygivenator molnari* and *Dinotyrannus megagracilis* to juvenile and subadult members, respectively, of *Tyrannosaurus rex* [36].

In this section we conduct an ontogenetic analysis to test the validity of previously named chasmosaurine taxa in the DPF by determining the ontogenetic status or ranges of referred specimens. If the ontogenetic null hypothesis (specimens referred to *Chasmosaurus belli* and *Chasmosaurus russelli* do not form separate, non-overlapping ontogenetic stages) is rejected, this indicates that the specimens represent discrete ontogenetic stages, which may correspond to the growth history of a single species.

To conduct this analysis we identified 18 ontogenetically variable cranial characters (see S7 File) within *Chasmosaurus* based on previous ontogenetic studies of ceratopsids (e.g., [37–43]) and other dinosaurs (*Protoceratops* [44]; *Psittacosaurus* [45]), and studies on the pattern of skull suture closure in both extant [46] and fossil [43] taxa. These characters were then assigned character states following the methodologies of Carr & Williamson [36] and Carr [34] where the least mature state is assigned '0' and successively more mature states are assigned successively higher numerical values. The characters pertain to the articulation of epiossifications onto the margin of the skull (1, 2, 11, 14, and 17), inferred remodelling of bone (3, 4, 13, 15, and 16), articulation between elements of the skull roof (characters 5–10), and dimensional changes in cranial elements (12) that have been suggested to vary with age [39, 43, 47]. In this study, “articulation” refers to when two or more cranial elements are in direct contact with each other, as preserved.

Fourteen skulls were included in the analysis because they are relatively complete and express key transitional ontogenetic changes. An artificial embryo, coded “0” for all characters, was used as the outgroup to root the tree and polarize the characters (see [34, 36]). The analysis was conducted on the resulting matrix (S7 File) using the tree bisection reconnection search algorithm, with 10 trees saved per replication (n = 1000). We started with Wagner trees, with a random seed of one. Eight characters (1, 2, 4, 11, 12, 14, 15, and 17) were ordered, as they have three sequential states. The analysis (strict consensus) and corresponding tree statistics (Bootstrap values and Bremer support) were conducted in TNT 1.1 [23, 24]. Bootstrap values were calculated using 1000 replicates. The matrix is provided in TNT and NEXUS formats in S8 and S9 Files, respectively.

### Permits

The *Chasmosaurus russelli* holotype (CMN 8800) quarry, located at the Onefour Research Station, Alberta, was accessed in July, 2012, with Government of Alberta Permits To Excavate Palaeontological Resources Nos. 12–008 issued to D. Evans (ROM) and 12–034 issued to M. Ryan (CMNH). Although an excavation was not conducted, weathering and erosion since the specimen was originally collected have not exposed any additional material. All necessary permits were obtained for the described study, which complied with all relevant regulations.

## Results

### Stratigraphic analysis

**Fig 4:**
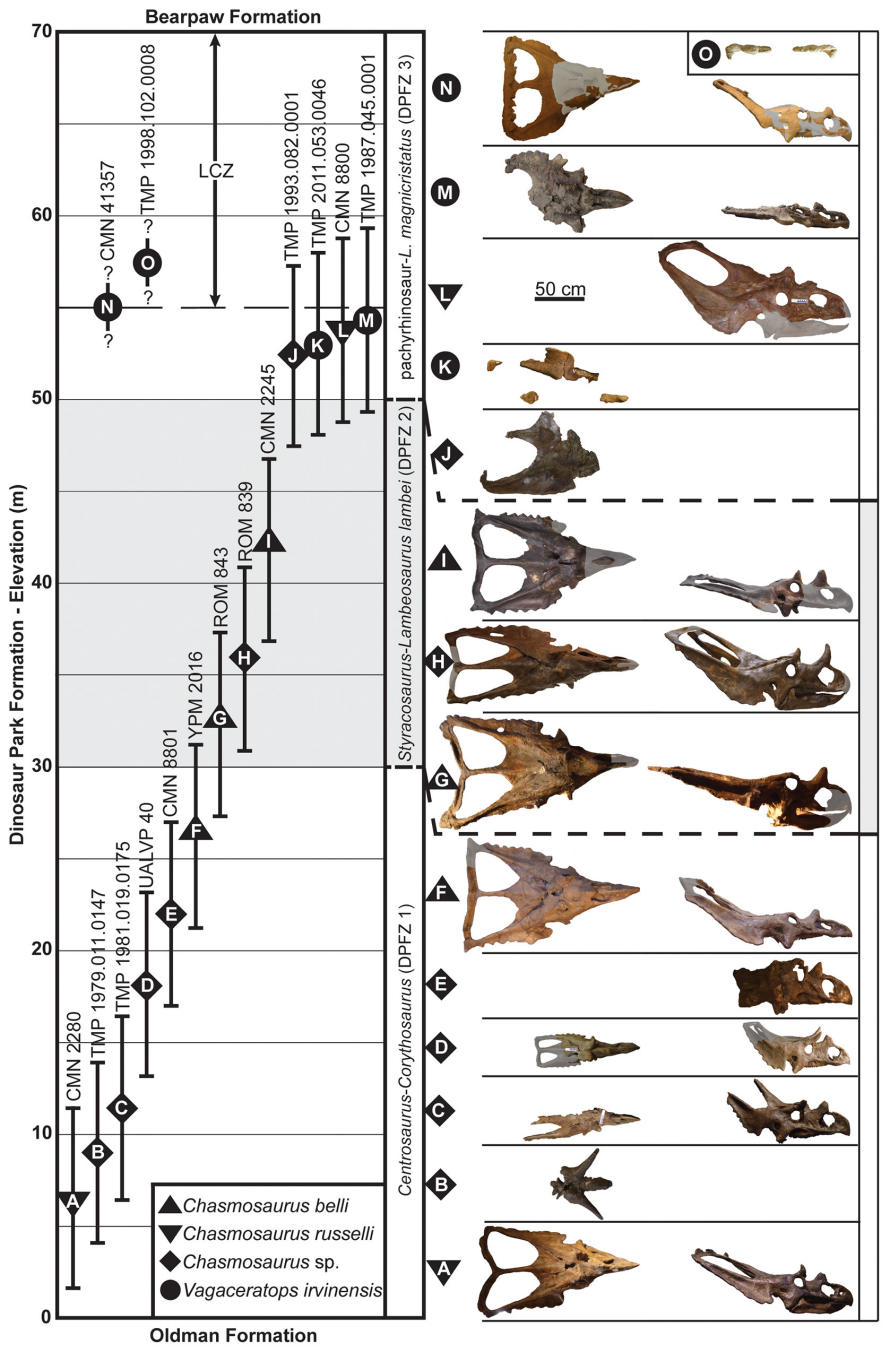
Stratigraphic positions of chasmosaurine specimens from the Dinosaur Park Formation. Specimens above the regional disconformity (0 m) separating the Dinosaur Park and Oldman formations (error bars ±5 m). Specimens are shown on right in ascending stratigraphic order, in dorsal and lateral views. CMN 41357 was collected either at the base of or within the Lethbridge Coal Zone (LCZ), and TMP 1998.102.0008 was collected somewhere within the LCZ, as indicated by the vertical dashed lines. DPFZ = Dinosaur Park Faunal Zone ([15, 16]). Plaster reconstruction = grey. [planned for page width].

The stratigraphic sequence of DPF chasmosaurine specimens are shown in Fig 4. The stratigraphic ranges of *C*. *belli* (i.e., YPM 2016 = 26.5 m to 42.0 m above the Oldman Formation = CMN 2245) and *C*. *russelli* (i.e., CMN 2280 = 6.6 m to approximately 54 m = CMN 8800) represent approximately 209 and 638 Kyr, respectively, following the recently calculated sedimentation rates of the DPF in DPP (D. Eberth, pers. comm.). The relatively large stratigraphic range of *C*. *russelli* is the result of recently conducted fieldwork, which revealed that the holotype (CMN 8800) of this species was recovered from the upper DPF, and not the lower DPF as previously supposed. This sequence supports the stratigraphic null hypothesis of no temporal separation between *Chasmosaurus* specimens previously referred to *C*. *belli* and *C*. *russelli* (Fig 4; *contra* [5, 15, 16]).

### Phylogenetic analysis

**Fig 5:**
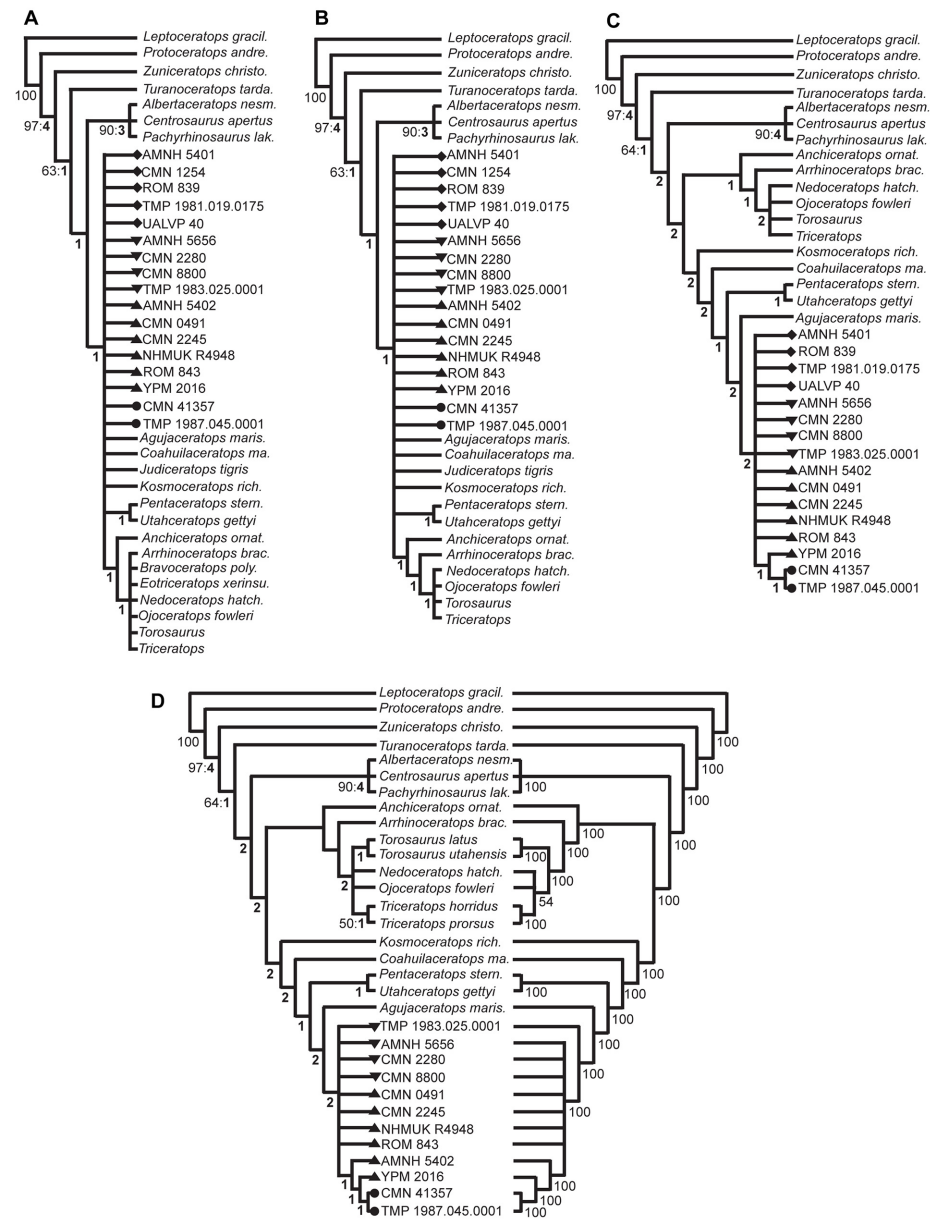
Specimen-based phylogenetic analysis of *Chasmosaurus* and *Vagaceratops* specimens, using 153 cranial and postcranial characters. Strict consensus trees of: (A) *Chasmosaurus*, *Vagaceratops*, and other taxa included in Campbell [22] (5410 most parsimonious trees, tree length (TL) = 293 steps, consistency index (CI) = 0.61, retention index (RI) = 0.76); (B) same taxa as ‘A’, but excluding *Bravoceratops* and *Eotriceratops* (*sensu* [48]) (TL = 282 steps, CI = 0.63, RI = 0.77); (C) same taxa as ‘B’, but excluding *Judiceratops* and CMN 1254, the latter of which is the most fragmentary of those specimens missing the species-specific posterior frill region (TL = 279 steps, CI = 0.63, RI = 0.77); (D) same taxa as ‘C’, but excluding AMNH 5401, ROM 839, TMP 1981.019.0175, and UALVP 40, which are also missing the posterior frill region (TL = 276 steps, CI = 0.64, RI = 0.76; 50% majority rule consensus tree on right). Bootstrap replicate frequency and Bremer support (bold) values are shown below each node; only Bootstrap values of 50% or higher are given. *Chasmosaurus belli* (triangles), *C*. *russelli* (inverted triangles), *Chasmosaurus* sp. (diamonds), and *Vagaceratops* specimens (circles). [planned for page width].

The phylogenetic analysis produced a strict consensus tree (5410 most parsimonious trees, tree length (TL) = 293 steps, CI = 0.61, RI = 0.76; Fig 5A) with a poorly resolved Chasmosaurinae clade. In an attempt to improve resolution within this clade, the fragmentary *Bravoceratops* and *Eotriceratops* were excluded (*sensu* [48]); however, this change had little effect on the tree topology (TL = 282 steps, CI = 0.63, RI = 0.77; Fig 5B). The subsequent exclusion of the fragmentary *Judiceratops* had no effect on the tree topology. The further exclusion of the most fragmentary specimen (CMN 0491, *C*. *belli* holotype) also had no effect; this specimen was retained in later tree iterations, as it preserves the diagnostic posterior frill margin (see Results).

The most fragmentary (CMN 1254, *C*. *canadensis* holotype) of the specimens not preserving the posterior parietal bar (parameter 33, Fig 2) was subsequently excluded. This greatly improved the resolution of the tree (TL = 279 steps, CI = 0.63, RI = 0.77; Fig 5C) and yielded the clade (YPM 2016 + (CMN 41357 + TMP 1987.045.0001)) which forms a polytomy with other specimens. The further exclusion of all other specimens missing the posterior frill margin (AMNH 5401, CMN 1254, ROM 839, TMP 1981.019.0175, and UALVP 40) yielded the clade (AMNH 5402 + (YPM 2016 + (CMN 41357 + TMP 1987.045.0001))) (TL = 276 steps, CI = 0.64, RI = 0.76; Fig 5D left). The rest of the DPF chasmosaurine specimens, all previously referred to *C*. *belli* (CMN 2245, NHMUK R4948, and ROM 843) and *C*. *russelli* (AMNH 5656, CMN 2280, CMN 8800, and TMP 1983.025.0001), form a monophyletic clade. None of these specimens can be differentiated on the basis of the characters used in this analysis. The latter strict consensus tree is similar to the strict reduced consensus tree of Mallon *et al*. ([48]: Fig 15B), except that: the centrosaurine clade is unresolved; ‘*Mojoceratops*’ (TMP 1983.025.0001 and AMNH 5656) clusters with *Chasmosaurus* specimens; and *Vagaceratops* (CMN 41357 and TMP 1987.045.0001) is a sister taxon to *Chasmosaurus* and not to *Kosmoceratops* (Fig 5D left). The corresponding 50% majority rule tree (Fig 5D right) has a similar topology to the strict consensus tree (Fig 5D left), except that: *Torosaurus* is an outgroup to the (*Nedoceratops* + *Ojoceratops* + *Triceratops*) clade; and TMP 1983.025.0001 is basal to other DPF chasmosaurine specimens.

### Morphometric analysis

**Fig 6:**
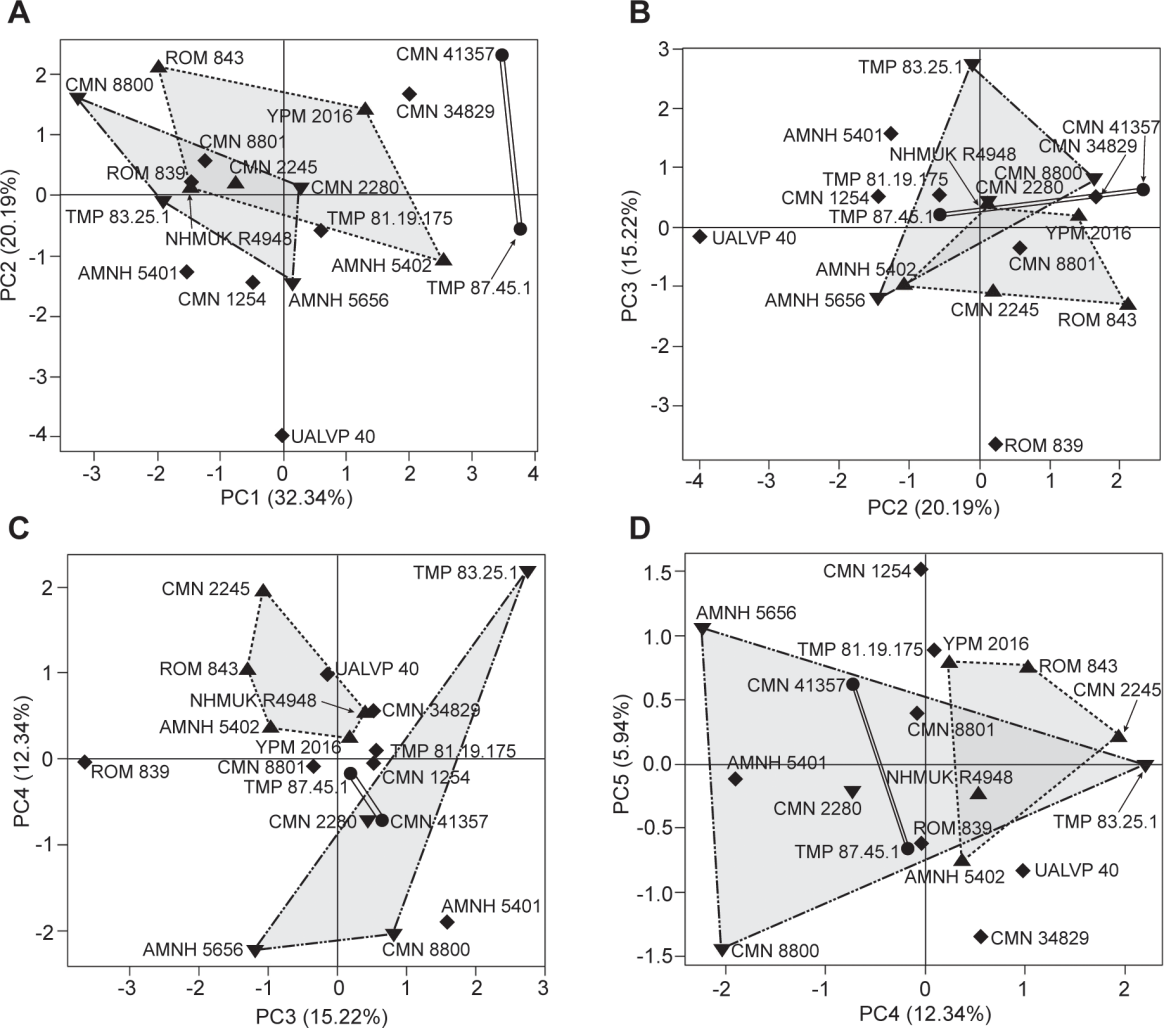
Principal component analysis (PCA 1) of *Chasmosaurus* and *Vagaceratops*. PCA of 18 specimens of *Chasmosaurus belli* (triangles; n = 5), *C*. *russelli* (inverted triangles; n = 4), *Chasmosaurus* sp. (diamonds; n = 7), and *Vagaceratops irvinensis* (circles; n = 2): (A) PC1 vs. PC2 (52.5% of variance), (B) PC2 vs. PC3 (35.4% of variance), (C) PC3 vs. PC4 (27.6% of variance), and (D) PC4 vs. PC5 (18.3% of variance). [planned for page width].

In PCA 1, the first five PCs account for 86.0% of the total variance (Fig 6; Table A in S10 File). In PC1 and PC2, most parameter loadings are subequal, indicating that skull size is a major influence on the variation in these axes (Table A in S10 File). Variation along the remaining PC axes is largely independent of size. The PC1 and PC2 axes in PCA 1 are significantly correlated (*p*<0.05 and 0.01, respectively) with parameter 17, and hence skull size.

**Fig 7:**
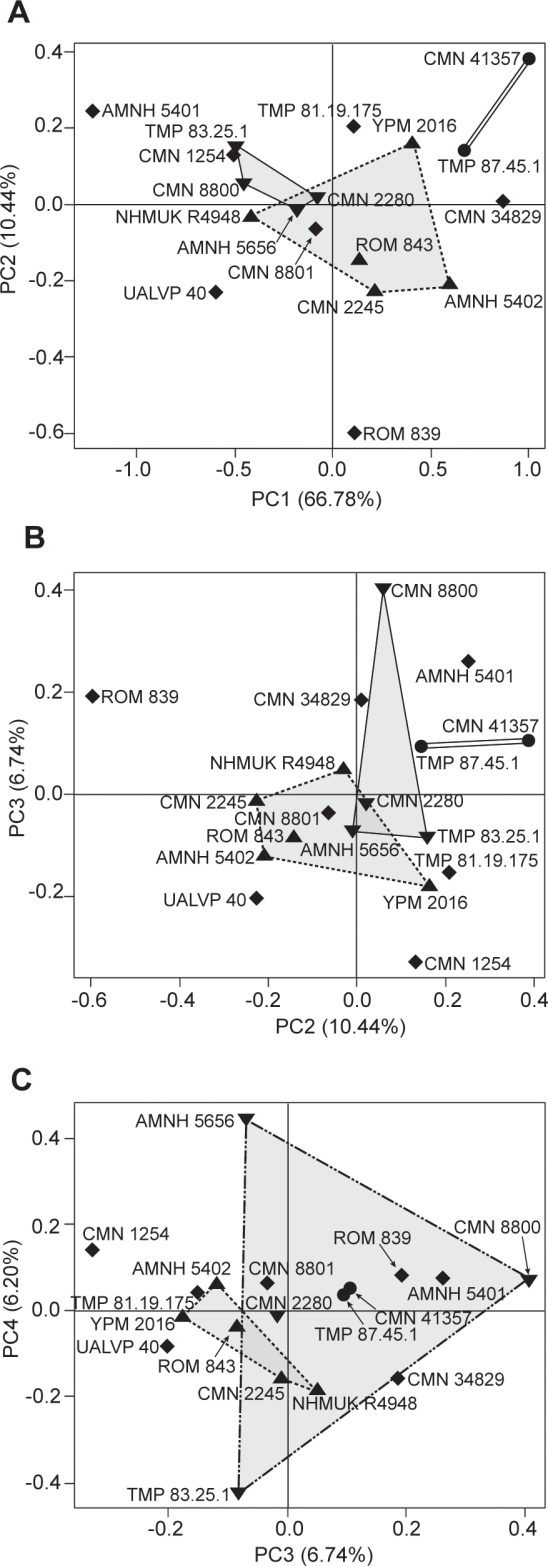
Principal component analysis (PCA 2) of *Chasmosaurus* and *Vagaceratops*, controlling for ontogenetic differences. PCA of 18 specimens of *Chasmosaurus belli* (triangles; n = 5), *Chasmosaurus russelli* (inverted triangles; n = 4), *Chasmosaurus* sp. (diamonds; n = 7), and *Vagaceratops irvinensis* (circles; n = 2): (A) PC1 vs. PC2 (77.2% of variance); (B) PC2 vs. PC3 (17.2% of variance); and (C) PC3 vs. PC4 (12.9% of variance). [planned for column width].

In PCA 2, the first four PCs account for 90.2% of the total variance (Fig 7; Table B in S10 File). There is considerable variation in parameter loadings along each of these axes, indicating that skull size is not a major influence on the variation in these axes. There are no significant differences between clusters of *C*. *belli* and *C*. *russelli* specimens along any PC axis, or combination of axes, for either PCA 1 or PCA 2. However, these clusters do show separation, albeit non-significant, along the combined PC3 and PC4 axes (27.6% of variance) in PCA 1 (Fig 6). The highest loading parameters on these combined axes, in descending order of importance, indicate that *C*. *belli* specimens have a relatively wider parietal (parameter 27), a dorsoventrally thinner medial parietal bar (parameter 31), a longer nasal horncore (parameter 5), and a wider medial parietal bar (parameter 32) (Table B in S10 File).

Although specimens previously referred to *C*. *belli* (AMNH 5402, CMN 0491, CMN 2245, NHMUK R4948, ROM 843, and YPM 2016) and *C*. *russelli* (AMNH 5656, CMN 2280, CMN 8800, CMN 8803, and TMP 1983.025.0001) cannot be significantly differentiated in either PCA 1 or 2, they can be distinguished from each other by the relative embayment of the posterior parietal bar (parameter 33, Fig 2; 136° to 180°, Fig 3H–3N, vs. 89° to 128°, Fig 3A–3G, respectively); parameter 33 was not included in PCAs 1 or 2.

### Ontogenetic analysis

**Fig 8:**
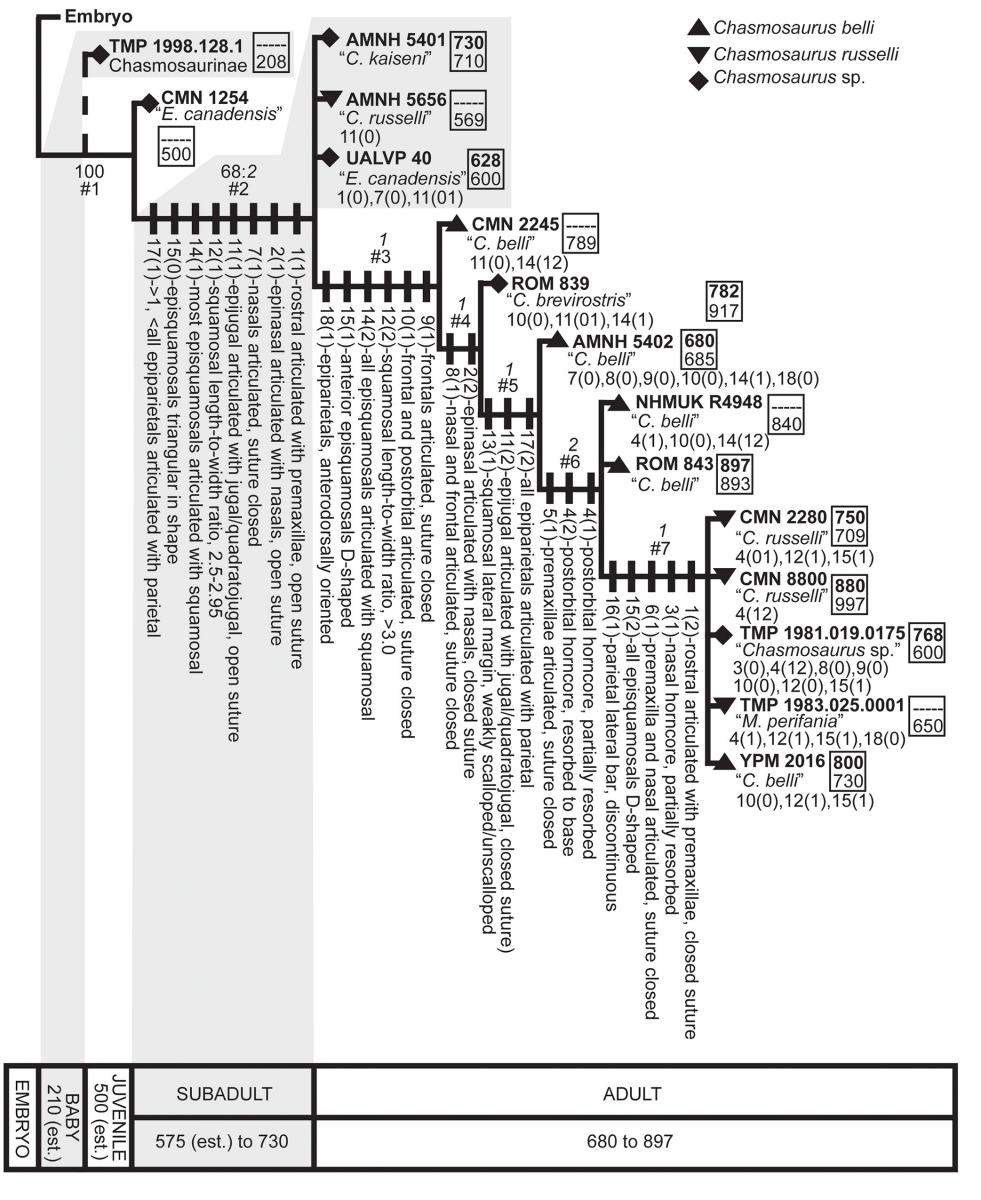
Ontogenetic transformation series for *Chasmosaurus* specimens. Strict consensus of 24 most parsimonious trees (TL = 40 steps, CI = 0.65, RI = 0.81) using 18 cranial characters. Bootstrap replicate frequency is shown above each node (only Bootstrap values of 50% or higher are given); Bremer values are shown above each node, in italics. Characters and associated states (shown in brackets) are shown on left and are mapped at their earliest occurence on the tree. These character state changes represent an inferred sequence of development; however, due to variable timing of character changes amongst specimens, this sequence is an approximation. Below each specimen is the previous taxonomic designation; state reversals are listed below. In box to the right of each specimen, rostral-to-epijugal length (top, bold, in mm; parameter 16, Fig 2) and squamosal length (bottom, in mm; parameter 19, Fig 2) are given for skull size reference, where preserved. Although TMP 1998.128.0001 was not included in the analysis, its basal position on the tree is indicated by a dashed line, inferred based on its small size and possession of immature character states (12(0), 13(0), and 14(0)). Boxes at bottom delimit age classes (see text for definitions); numbers below each box represent rostral-to-epijugal lengths (in mm) for each age class (est. = estimated). [planned for page width].

The analysis produced a strict consensus tree of 24 most parsimonious trees (tree length = 40 steps, consistency index = 0.65; retention index = 0.81) composed of eight groupings of successively more mature skulls (Fig 8). The first occurrence of a given character state in the tree, as well as character state reversals, are shown in Fig 8. Specimens referred to *C*. *belli* and *C*. *russelli* do not form discrete ontogenetic stages, but instead have considerable ontogenetic overlap.

## Discussion

Our analyses of *Chasmosaurus* specimens from the Dinosaur Park Formation (DPF) support the validity of *C*. *belli* and *C*. *russelli* based on a single character, the degree of embayment of the posterior parietal bar. Although this character is intuitively obvious when observing the specimens first hand, it can only be statistically confirmed through the analysis of continuous data in a principal component analysis (PCA), rather than as a discrete character in linear statistical analyses. Although we quantified this embayment character in our phylogenetic analysis, its states, as defined, do not differentiate specimens previously referred to *C*. *belli* and *C*. *russelli*. In the latter analysis, resolution is poor with even the subfamily Chasmosaurinae only being resolved after the exclusion of *Bravoceratops*, *Eotriceratops*, *Judiceratops*, and CMN 1254 (Fig 5B and 5C). The subsequent removal of the other DPF chasmosaurine specimens missing their diagnostic posterior frill margins (AMNH 5401, ROM 839, TMP 1981.019.0175, and UALVP 40) did not differentiate taxa within *Chasmosaurus*, but did resolve *Vagaceratops* (and the *Chasmosaurus* skulls AMNH 5402 and YPM 2016) as the sister group to *Chasmosaurus*. This lack of resolution for the *Chasmosaurus* specimens (Fig 5D) (excluding AMNH 5402 and YPM 2016) is interpreted as being due to an insufficient number of character states describing the relative degree to which the posterior parietal bar is embayed, making it difficult to differentiate specimens of *Chasmosaurus* when coding this character.

We acknowledge the fact that the support for *C*. *belli* and *C*. *russelli* is based on a limited sample size (*C*. *belli*, AMNH 5402, CMN 0491, CMN 2245, NHMUK R4948, ROM 843, and YPM 2016; *C*. *russelli*, AMNH 5656, CMN 2280, CMN 8800, CMN 8803, and TMP 1983.025.0001), and that the angle degree difference separating these taxa (*C*. *russelli* = 89° to 128°, Fig 3A–3G, vs. 136° to 180° = *C*. *belli*, Fig 3H–3N) is rather small. While these taxa currently can be distinguished on the basis of this character, the discovery of more specimens will be necessary to corroborate this apparent bimodal distribution.

Godfrey & Holmes [3] also used two additional characters to support the validity of *C*. *belli* and *C*. *russelli*: (1) the presence/absence of complete lateral parietal bars in conjunction with the embayment of the posterior bar (continuous/weakly embayed = *C*. *belli*; discontinuous/strongly embayed = *C*. *russelli*); and (2) three, subequal epiparietals (*C*. *russelli*) vs. enlarged epiparietal (*C*. *belli*) on the posterolateral corner of the parietal. For the former character, as noted by Maidment & Barrett [7], the morphology of the two linked features are not discretely distributed across the two taxa, e.g., AMNH 5656 (Fig 3E; *C*. *russelli*) has an embayed posterior bar with continuous lateral bars, and YPM 2016 (Fig 3M; *C*. *belli*) has a straight posterior bar and discontinuous lateral bars. This variation is probably attributable to ontogenetic and/or individual variation. For the latter character, the morphology of the three epiparietals are not unique to each taxon. For example: the epiparietals of AMNH 5656 (Fig 3E) and CMN 8800 (Fig 3A), both referred to *C*. *russelli*, are variable enough to not be considered ‘subequal’ in size; and, the P3 position of ROM 843, referred to *C*. *belli*, is interpreted here as being occupied by a P2 on each side of the parietal.

Additionally, the use of the stratigraphic separation of the two taxa to support their taxonomic assignments (Godfrey & Holmes [3]) is no longer supported with the recognition that the holotype of *C*. *russelli* (CMN 8800) comes from the top of the DPF, meaning both taxa overlapped in time. While the temporal range of specimens previously referred to *C*. *belli* (209 Kyr) is within the range of other dinosaur taxa in the Dinosaur Park Formation, that of *C*. *russelli* (638 Kyr) is unusually long [49]. Dinosaurs with similarly long (or longer) temporal ranges are known, but generally occur in younger strata such as the Horseshoe Canyon Formation of Alberta [50, 51], e.g., *Arrhinoceratops brachyops* = 860 Kyr [52] and *Anchiceratops ornatus* = 1.5 to 2.0 Myr [53]. Differing hypotheses have been presented to account for the discrepancy in species longevity between these formations [51, 53], which are summarized in Mallon *et al*. [48]. The discovery of more *Chasmosaurus* specimens exhibiting the ‘*C*. *russelli’* condition (i.e., relatively deep posterior parietal embayment), and intermediate to the stratigraphic end members of this species (CMN 2280 and CMN 8800; Fig 4), will help to better understand the apparently long temporal range of this species.

In the morphometric analysis, the only separation, albeit non-significant, between specimens of *C*. *belli* and *C*. *russelli* was along the combined principal component 3 and 4 axes of PCA 1 (Fig 6). None of the highest-loading parameters (5, 27, 31, 32; Table B in S10 File) along these axes have been previously used in distinguishing these two species.

### *Chasmosaurus* ontogeny

Our ontogenetic analysis identified 18 characters that are ontogenetically variable, similar to the number determined by Longrich & Field ([43]; n = 24) for *Triceratops*.

Specimens referred to *C*. *belli* and *C*. *russelli* are distributed randomly across the ontogenetic tree (Fig 8), supporting the ontogenetic null hypothesis of no ontogenetic separation between these taxa. The overlapping growth histories of *C*. *belli* and *C*. *russelli* specimens may correspond to two distinct but ontogenetically conservative taxa, or they could be interpreted as a variable growth history of a single taxon, resulting from individual or sexual dimorphism (Fig 8). However, the latter scenario is unlikely, given the large degree of variability in both the posterior parietal embayment and size of the postorbital horncores in *Chasmosaurus*.

In general, increased maturity corresponds positively with increased skull size (Fig 8; [31, 41]). As in *Triceratops* [39], *Agujaceratops* [54], *Centrosaurus apertus* [38], and *Pachyrhinosaurus lakustai* [55], small, immature *Chasmosaurus* skulls are rare, probably the result of taphonomic bias against small, relatively delicate individuals [56]. As a result, data on growth trajectories of chasmosaurines is limited, with *Triceratops* providing the most complete dataset [39].

The sequence of successively more mature groupings in Fig 8 provide a framework for subdividing the ontogenetic spectrum of *Chasmosaurus*, and allow us to infer separate ontogenetic stages/growth categories for the specimens examined.

**Fig 9:**
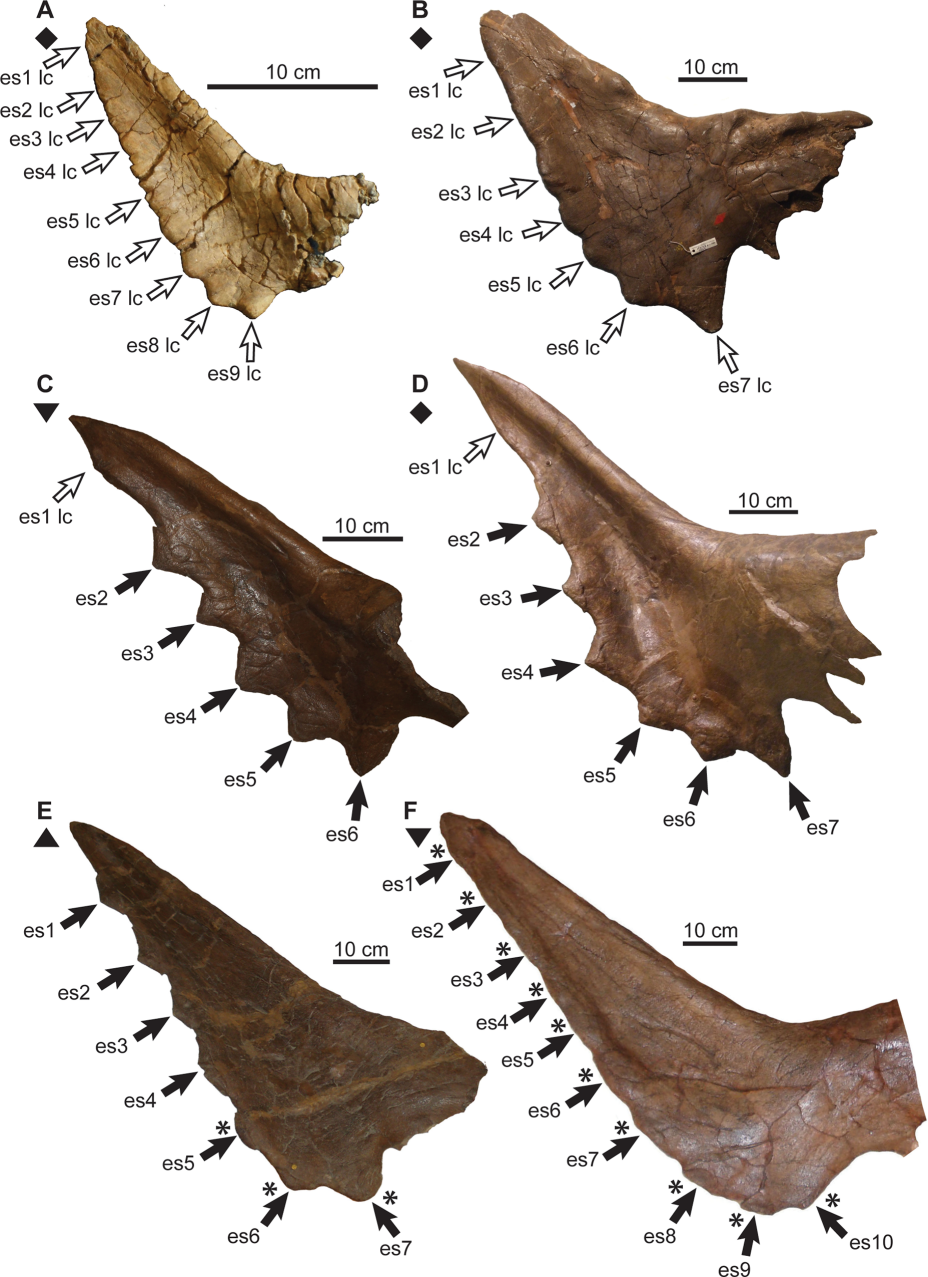
Ontogenetic changes in squamosal of *Chasmosaurus*. Increase in length/width ratio (character 12), changing outline of lateral margin (character 13), articulation (character 14) and remodeling (character 15) of episquamosals: (A) TMP 1998.128.0001 (flipped), (B) CMN 1254, (C) AMNH 5656, (D) AMNH 5401, (E) NHMUK R4948 (courtesy of J. Mallon), and (F) CMN 8800. Blank arrows = unadorned episquamosal loci; solid arrows = articulated episquamosals; solid arrows with asterisks = remodeled episquamosals. *Chasmosaurus belli* (triangle), *Chasmosaurus russelli* (inverted triangles) and *Chasmosaurus* sp. (diamonds). [planned for page width].

*Baby*: This is the ontogenetically least developed and smallest skull size category, with the rostral-to-epijugal length ranging up to approximately 250 mm. The isolated squamosal TMP 1998.128.0001 (Fig 9A) was not included in this analysis, but probably belongs to this stage. The relatively small squamosal length/width ratio (2.0) of this specimen, as well as its pronounced and unadorned episquamosal loci, are typical of relatively small, immature chasmosaurine individuals (e.g., *Triceratops*; [42, 43]).

**Fig 10:**
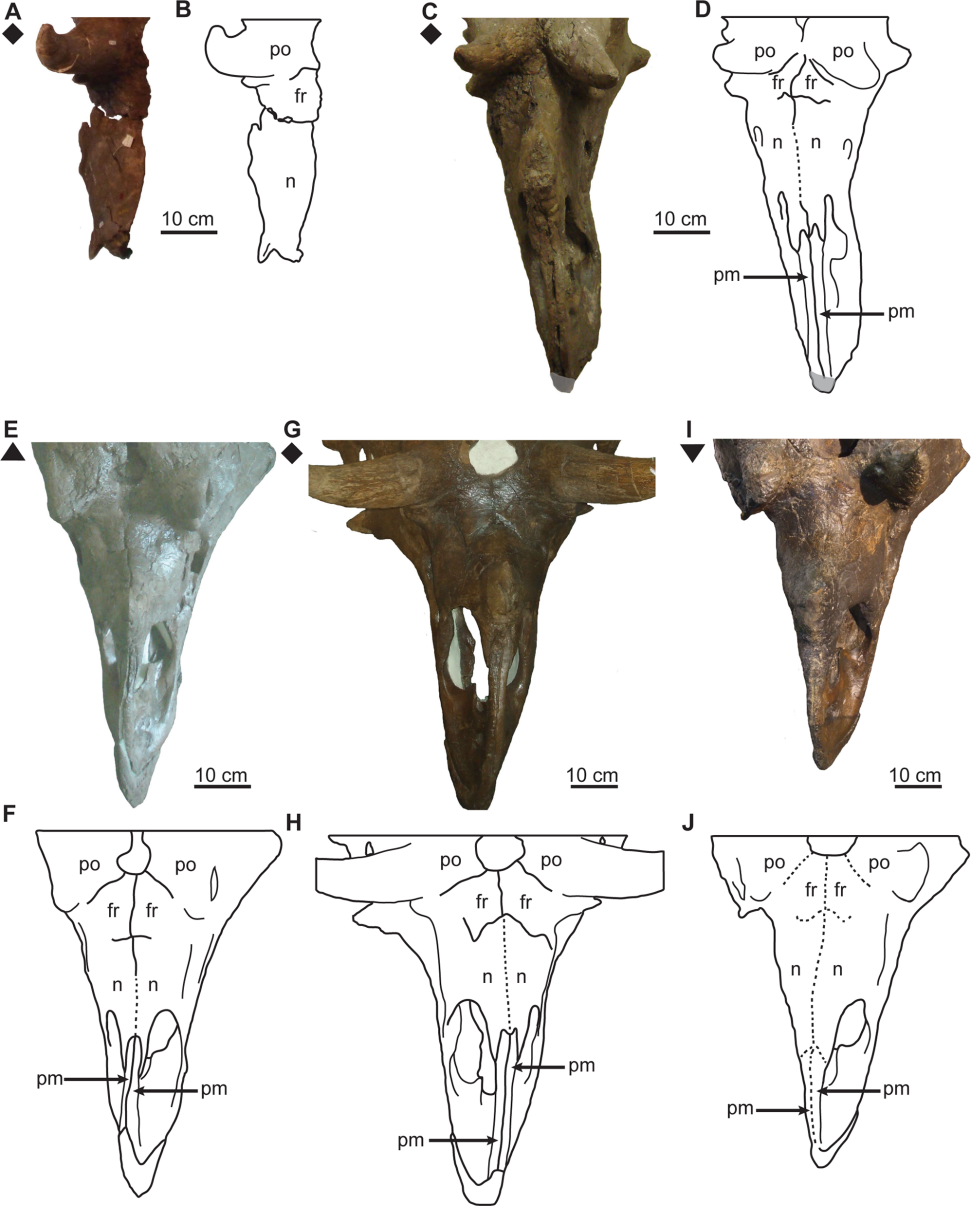
Ontogenetic changes in skull roof of *Chasmosaurus*. Articulation between: premaxillae (character 5), premaxilla-nasal (character 6), nasals (character 7), nasal-frontal (character 8), frontals (character 9), and frontal-postorbital (character 10): (A–B) CMN 1254, (C–D) UALVP 40, (E–F) AMNH 5402, (G–H) AMNH 5401 (courtesy of J. Mallon), and (I–J) CMN 2280. Solid lines = open sutures; dotted lines = closed sutures; plaster reconstruction = grey. *Chasmosaurus belli* (triangle), *Chasmosaurus russelli* (inverted triangle), and *Chasmosaurus* sp. specimens (diamonds). [planned for page width].

**Fig 11:**
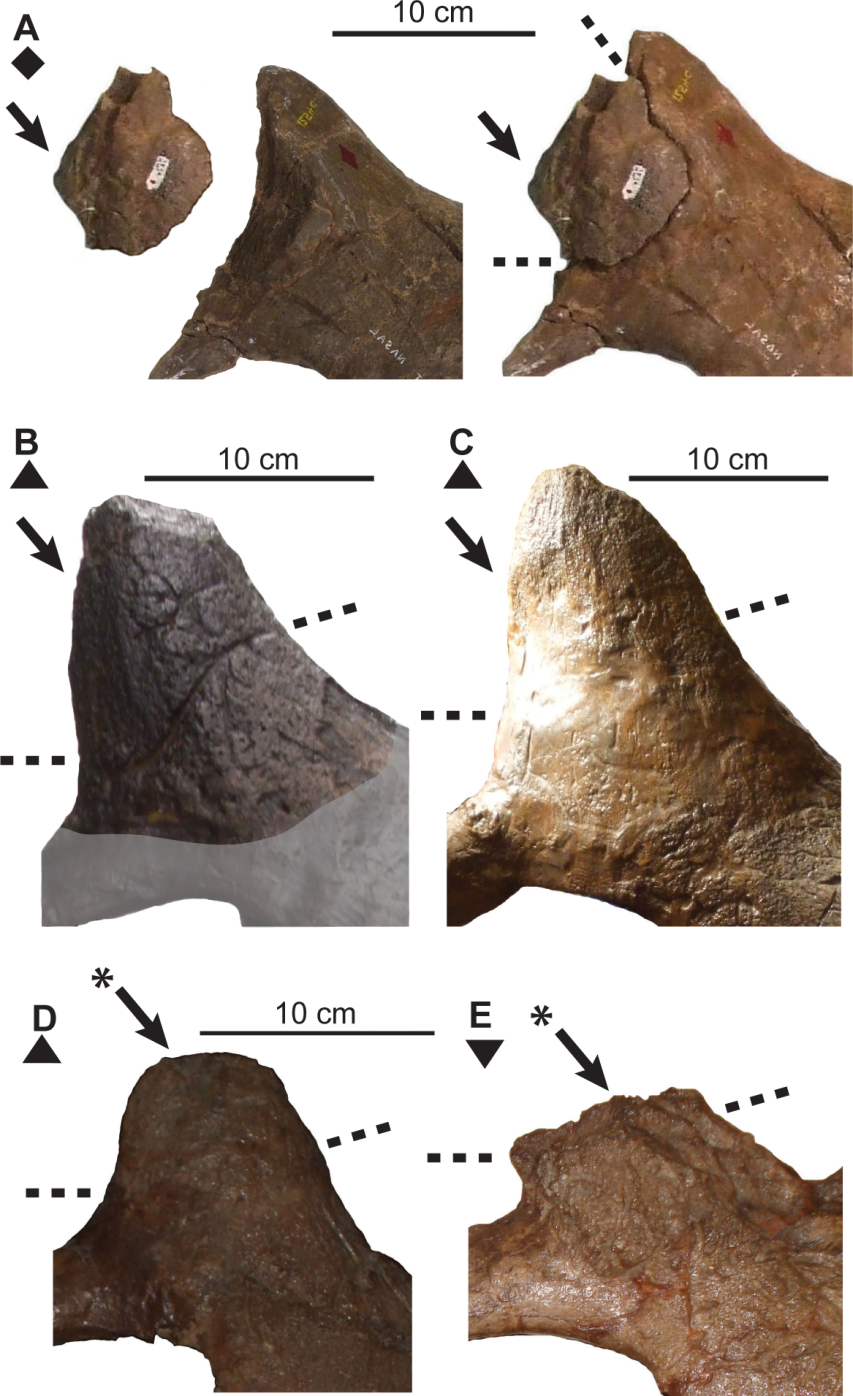
Ontogenetic changes in nasal horncore of *Chasmosaurus*. Articulation of epinasal with nasals (character 2) and subsequent resorption of nasal horncore (character 3): (A) epinasal disarticulated (CMN 1254; flipped), (B) epinasal articulated with suture open (CMN 2245), (C) epinasal articulated with suture closed, and (D) (YPM 2016) and (E) (CMN 8800) horncore partly resorbed. Arrows = confirmed or inferred epinasals; arrows with asterisks = resorbed surfaces; dashed lines = confirmed or inferred sutural contacts; plaster reconstruction = grey. *Chasmosaurus belli* (triangles), *Chasmosaurus russelli* (inverted triangle), and *Chasmosaurus* sp. specimens (diamond). [planned for column width].

**Fig 12:**
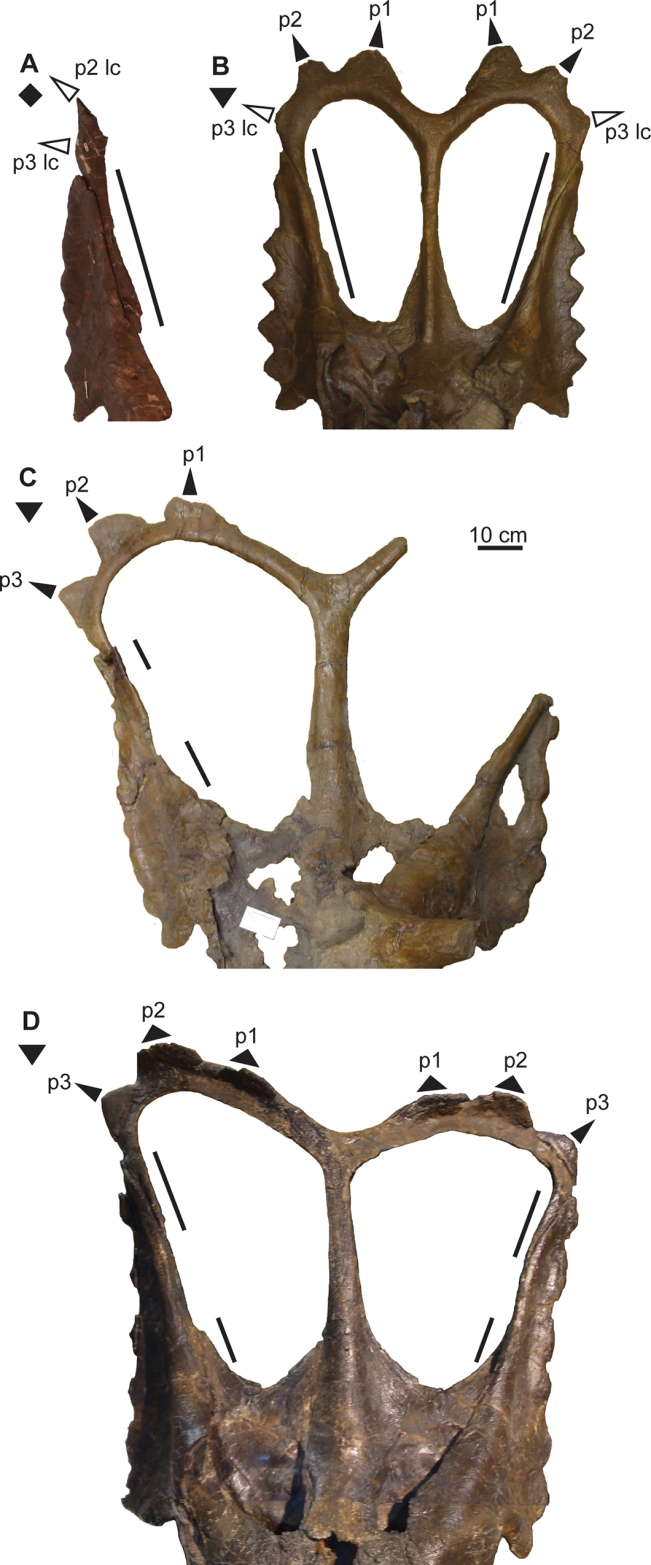
Ontogenetic changes in parietal of *Chasmosaurus*. Thinning of lateral bar (character 16), articulation (character 17) and reorientation (character 18) of epiparietals (character 18): (A) CMN 1254, (B) AMNH 5656, (C) TMP 1983.025.0001, and (D) CMN 2280 (flipped). Blank triangles = unadorned epiparietal loci; solid triangles = articulated epiparietals; long triangles = epiparietals (or loci) oriented posteriorly in the plane of the frill, and short triangles = epiparietals oriented anterodorsally. Lines represent extent of lateral bars. *Chasmosaurus russelli* (inverted triangles) and *Chasmosaurus* sp. (diamond). [planned for column width].

*Juvenile*: Juveniles have the same ontogenetic character states as the baby category, but are larger, with the rostral-to-epijugal length between approximately 250 mm and 550 mm. This category is represented by CMN 1254 (Node 1; *Chasmosaurus* (*Eoceratops*) *canadensis* holotype, Fig 9B), which has a squamosal length of 500 mm, and a length/width ratio of 2.0. This specimen exhibits several other traits typical of immaturity, including open sutures between the nasals, frontals, and postorbitals (Fig 10A and 10B), epinasal disarticulated from nasals (Fig 11A), and epiparietals disarticulated from parietal (Fig 12A; [42, 43]).

**Fig 13:**
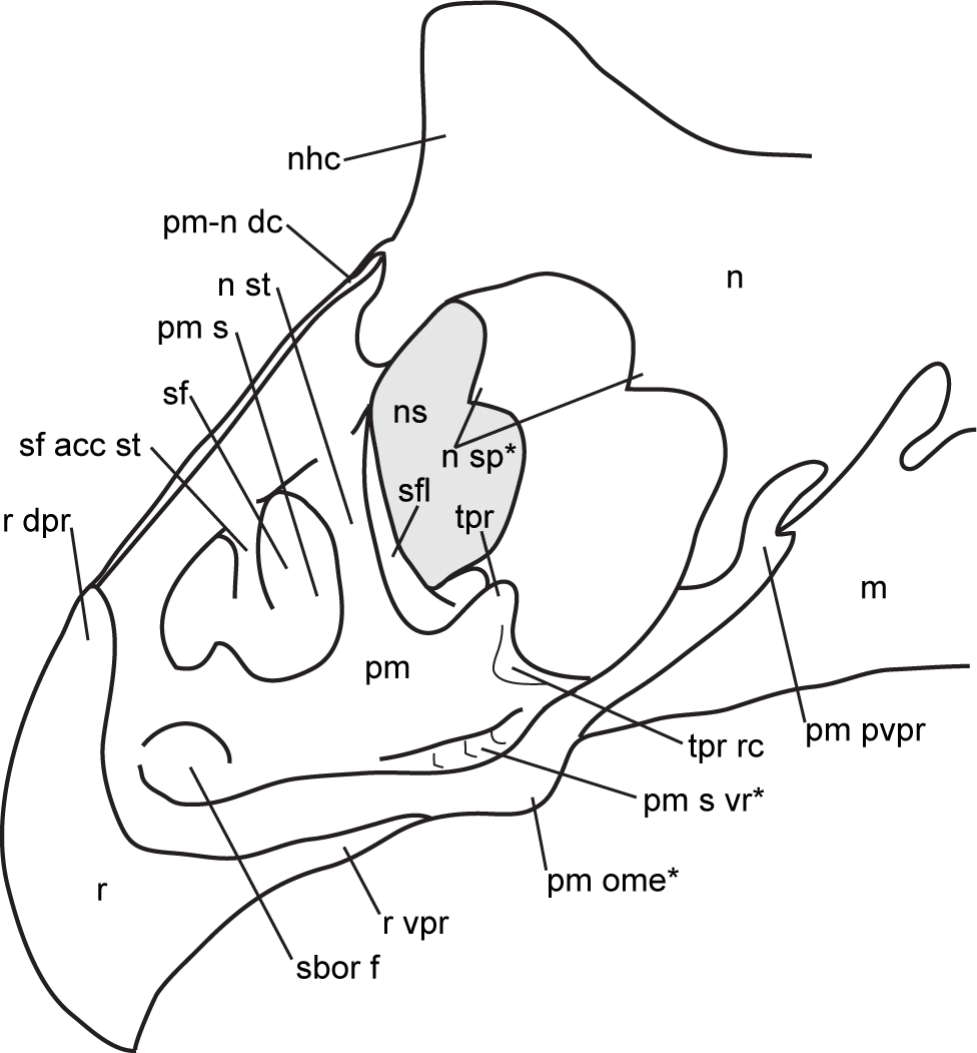
Schematic of an idealized chasmosaurine snout ([11]: Fig 8, modified). Centrosaurine features (i.e., not present in chasmosaurines) are indicated by asterisks (*) ([35]: fig 16.1). [planned for column width].

**Fig 14:**
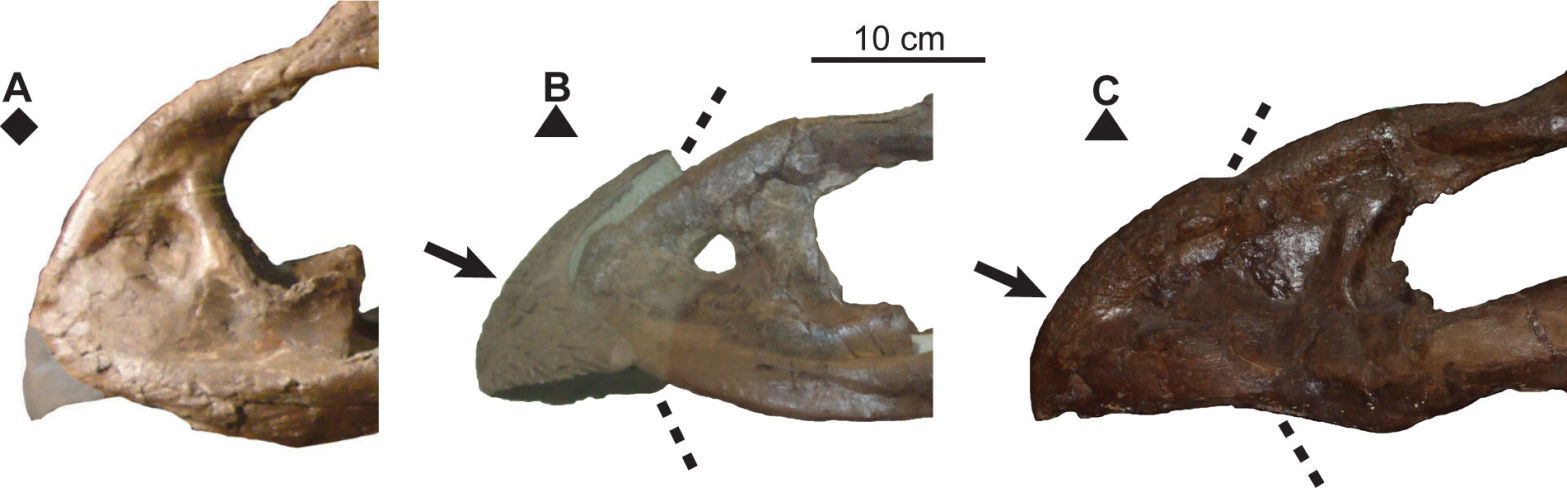
Ontogenetic changes in rostral of *Chasmosaurus*. Articulation of rostral with premaxillae (character 1): (A) rostral separate and missing (UALVP 40); (B) rostral articulated, but dorsal suture open (AMNH 5402); and (C) rostral articulated with suture closed (YPM 2016). Arrows = rostra; dashed lines = sutural contacts; plaster reconstruction = grey. *Chasmosaurus belli* (triangles) and *Chasmosaurus* sp. specimens (diamond). [planned for page width].

**Fig 15:**
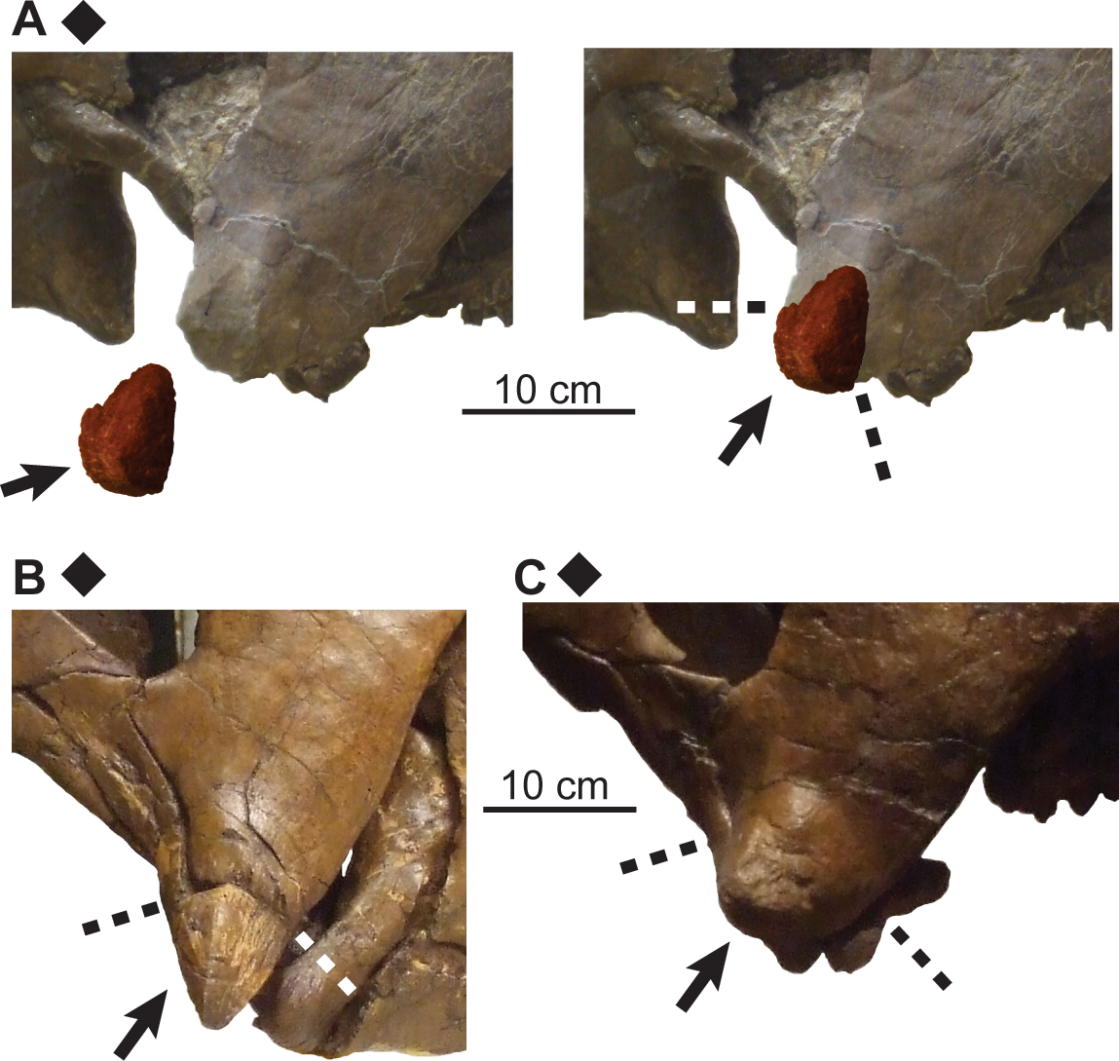
Ontogenetic changes in epijugal of *Chasmosaurus*. Articulation of epijugal with jugal and quadratojugal (character 11): (A) epijugal disarticulated (UALVP 40), (B) epijugal articulated with open suture (ROM 839) and (C) epijugal articulated with closed suture (TMP 1981.019.0175). Arrows = epijugals; dashed lines = sutural contacts. *Chasmosaurus* sp. (diamonds). [planned for column width].

*Subadult*: Subadult specimens (Node #2; AMNH 5401, AMNH 5656, and UALVP 40) are characterized by a rostral-to-epijugal length between approximately 550 mm and 750 mm, and otherwise can show a mosaic of juvenile and adult characteristics. This category is characterized by the articulation (but not sutural closure) of the rostral to the premaxillae (Figs 13 and 14B), the epinasal to the nasals (Fig 11B), the epijugal to the jugal and quadratojugal (Fig 15A and 15B), the triangular-shaped episquamosals to the squamosal (Fig 9C and 9D), as well as the epiparietals to the parietal (Fig 12B), as occurs over ceratopsid ontogeny [37, 39]. The anteroposterior sequence of episquamosal attachment in *Chasmosaurus* is consistent with other chasmosaurines [37]. The obliteration of sutures between elements of the skull roof, which occurs over ceratopsid ontogeny [43], begins at this stage, starting with the nasals. The squamosal length/width ratios (2.5 to 2.95) also increases as in *Triceratops* ontogeny [42].

The relatively long epijugal and postorbital horncore of AMNH 5401 were used by Brown [57] as diagnostic characters of *Chasmosaurus kaiseni*. However, the length of the epijugal is highly variable within *Chasmosaurus* specimens (Fig 15) and is likely attributable to ontogenetic, and likely individual and sexually dimorphic variation. As discussed below (see comments on postorbital horncore development), we do not consider the long-horned skull AMNH 5401 to be sufficiently preserved to determine whether it represents a species distinct from *C*. *russelli* or *C*. *belli*, as it is lacking the posterior parietal bar.

*Adult*: Adult specimens in the successively more mature nodes #3–7 are considered to be the largest and most ontogenetically derived. This category is characterized by rostral-to-epijugal lengths between approximately 750 mm and 900 mm, although smaller sizes also occur. Adult ontogimorphs would be expected to show all of the derived advanced characters including complete articulation of all epiossifications and obliteration of all sutures; however, some of the specimens examined here still retain some subadult characters.

At Node #3 (CMN 2245), sutural closure occurs between the frontals, and the frontals and postorbitals (Fig 10I and 10J). All episquamosals have articulated with the squamosal and the anteriormost episquamosals have been remodelled from triangular to D-shaped morphology (Fig 9E and 9F), which progresses in an anteroposterior direction in chasmosaurines [22, 37]. The squamosal length/width ratio exceeds 3.0.

The posterior parietal margin starts to curve anterodorsally, resulting in a similar orientation for the medialmost epiparietals (Fig 12E). In the early ontogenetic stages of ceratopsids, the posterior margin of the parietal is dorsoventrally thin and oriented in the plane of the frill, but thickens later in ontogeny as loci become more developed (e.g., *Centrosaurus* [38]; *Triceratops* [39]; *Styracosaurus* [58]). In *Centrosaurus apertus*, locus 1 recurves into an anterodorsal orientation, while the associated epiparietal develops into an elongate, anteriorly oriented hook [38]. Anterodorsally oriented epiparietals are also present in chasmosaurines other than *Chasmosaurus*, e.g., P1–4 of *Vagaceratops* (Fig 3O–3R; [19]); P1–3 of *Kosmoceratops* and P1 of *Utahceratops* [5]; and P1 of *Anchiceratops* [53]; the orientation and ontogenetic remodelling of the loci below the epiparietals in these latter four taxa are unknown, as immature specimens lacking epiparietals are unknown. The anterodorsal orientation of the loci underlying the medialmost epiparietals in adult *Chasmosaurus* is considered here to be ontogenetic in nature.

At Node #4 (ROM 839), sutural closure between the epinasal and nasals (Fig 11C), and the nasal and frontal occurs (Fig 10I and 10J). The relatively short snout of ROM 839 was used by Lull [59] as a diagnostic character of *Chasmosaurus brevirostris*. However, its length is emphasized by its anteroposteriorly short, partly reconstructed rostral, whose shape is within the range of other *Chasmosaurus* specimens (short in AMNH 5401 and TMP 1981.019.0175, and longer in others).

At Node #5 (AMNH 5402), the epijugal undergoes remodelling and obliterates its sutural contact with the jugal and quadratojugal (Fig 15C). It has been postulated that epijugals undergo remodelling with age, which may account for the variation in the length of this feature in *Chasmosaurus* (e.g., *Pachyrhinosaurus lakustai* [55]; [60]). The reduced scalloping of the lateral margin of the squamosal (Fig 9) at this stage is likely ontogenetic, as in *Triceratops* [42, 43], and is likely correlated with an elongation of the squamosal loci and the squamosal itself. All epiparietals have articulated with the parietal at this stage (Fig 12D).

**Fig 16:**
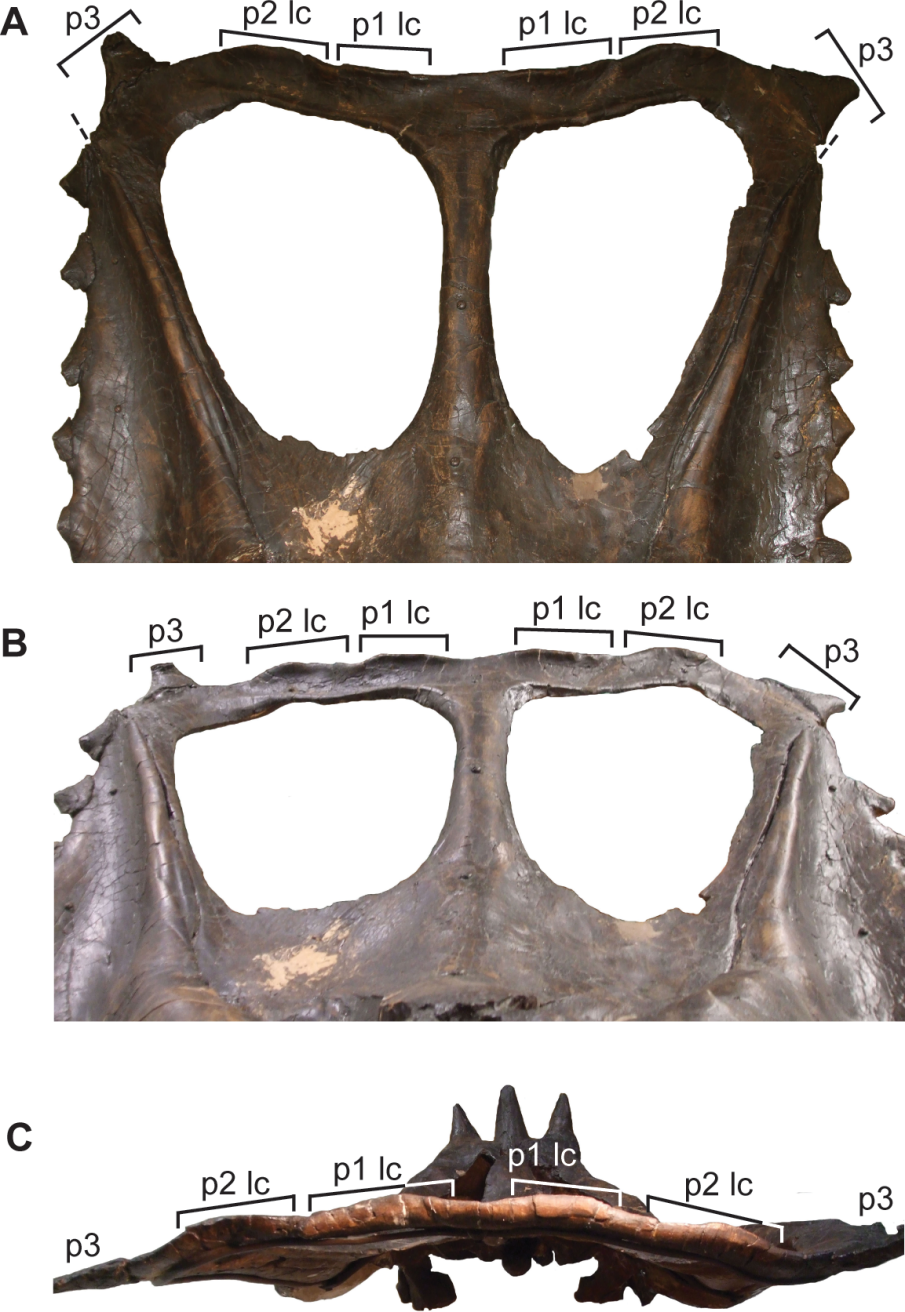
Frill of CMN 2245 (*Chasmosaurus belli*). Frill in: (A) dorsal, (B) anterior, and (C) posterior views. Brackets delimit size of epiossifications; dashed lines = parietal-squamosal contacts. [planned for column width].

Although an anteroposterior sequence of episquamosal attachment has been established for *Chasmosaurus*, no consistent pattern of epiparietal fusion has been demonstrated. While the traditionally accepted pattern of articulation (lateral to medial; [37]) can be inferred for some *Chasmosaurus* skulls (e.g., CMN 2245, Node #3, Fig 16), in several, the epiparietals appear to have articulated in a medial to lateral pattern. In AMNH 5656 (Node #2, Figs 3E and 12B), the P1s and P2s are articulated, but the P3 loci are unoccupied. In CMN 2280 (Node #7, Figs 3F and12D) and ROM 843 (Node #6, Fig 3H), all three epiparietals have articulated, but their basal sutures become increasingly obvious progressing laterally, suggesting a medial to lateral pattern.

**Fig 17:**
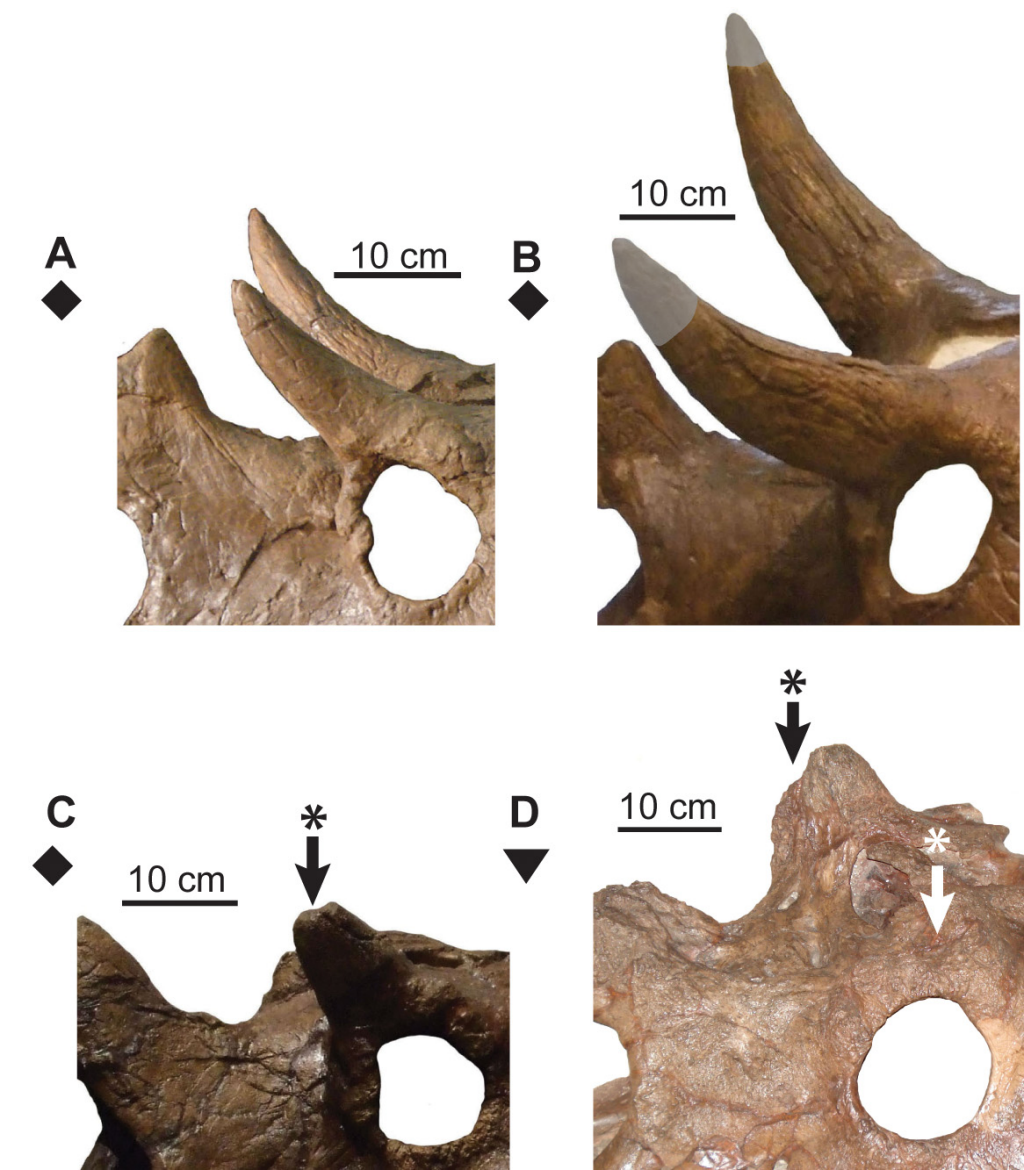
Ontogenetic changes in postorbital horncores of *Chasmosaurus*. Resorption of horncores (character 4): (A) (UALVP 40) and (B) (AMNH 5401; flipped), horncore complete; (C) horncore partly resorbed (TMP 1981.019.0175; flipped); and (D) horncore resorbed to base (CMN 8800). Arrows with asterisks = resorbed surfaces; plaster reconstruction = grey. *Chasmosaurus russelli* (inverted triangle) and *Chasmosaurus* sp. (diamonds). [planned for column width].

At Node #6 (NHMUK R4948 and ROM 843), the dorsal surface of the postorbital horncore undergoes partial to extensive resorption (Fig 17C and 17D). The restriction of resorbed horncores to relatively large specimens suggests that it is likely ontogenetic in nature (*sensu* [37]). Sutural closure between the premaxillae also occurs at this stage (Fig 10I and 10J).

At Node #7 (CMN 2280, CMN 8800, TMP 1981.019.0175, TMP 1983.025.0001, and YPM 2016), sutural closure between the rostral and premaxillae occurs. The nasal horncore also undergoes resorptive pitting (Fig 11D and 11E), similar to the postorbital horncore. Sutural obliteration of the skull roof is complete, with the closure between the premaxilla and nasal (Fig 10I and 10J). All the episquamosals become D-shaped (Fig 9F), and the lateral parietal bar becomes discontinuous (Fig 12C and 12D), possibly as a result of ontogenetic bone resorption. However, exceptions to this pattern (e.g., continuous bars in large skull ROM 843; Fig 3H) may represent individual variation. The continuity of the lateral bar also seems to vary in *V*. *irvinensis* (CMN 41357), which has a continuous left bar and discontinuous right bar ([19]; Fig 3R).

The lack of ontogenetic separation between the skull YPM 2016, which bears five epiparietals per side, and other skulls at Node #7, which bear three epiparietals per side (Fig 8), indicates that *Chasmosaurus* specimens with three epiparietal loci (adorned or not) per side do not mature into specimens with five per side. Also, the differing count cannot be attributed to post-mortem loss, as there is no room to add two more epiparietals onto each side of the posterior bar (e.g., AMNH 5656, Fig 3E; and CMN 2280, Fig 3F).

Maidment & Barrett [7] argued that *Mojoceratops perifania* (TMP 1983.025.0001; Fig 12D) is a junior subjective synonym of *C*. *russelli* based on their inability to confirm any of the autapomorphies of *Mojoceratops* identified by Longrich [6]. We support their conclusions with the following, additional observations. The deep grooves along the fenestral margins of the medial and posterior parietal bars of some *Chasmosaurus* specimens, resulting in an “I” cross sectional shape of the medial bar (e.g., CMN 8803, TMP 1983.025.0001, and TMP 1999.055.0292) were considered by Longrich [6] to be diagnostic of *M*. *perifania*. Although such grooves are most pronounced on the above specimens, they are also present on the *C*. *russelli* holotype, and reduced and restricted to the medial bar in some other specimens (e.g., CMN 2280 and YPM 2016). The variable expression of these grooves in *Chasmosaurus* likely represents individual variation. Furthermore, Longrich’s [6] provisional assignment of CMN 1254 and AMNH 5401 to *M*. *perifania* is controversial, as these specimens are the holotypes of *C*. *canadensis* [35] and *C*. *kaiseni* [57], respectively.

The establishment of a growth stage classification for *Chasmosaurus* allowed us to test the validity of previously referred chasmosaurine taxa in the DPF. The holotypes of *C*. (*Eoceratops*) *canadensis* (CMN 1254) and *C*. *kaiseni* (AMNH 5401) represent a juvenile and old subadult, respectively, and otherwise cannot be distinguished from other *Chasmosaurus* specimens. The holotypes of *C*. *brevirostris* (ROM 839) and *M*. *perifania* (TMP 1983.025.0001) represent relatively young and old adults, respectively, but do not possess autapomorphies or unique suites of apomorphies to support their taxonomic distinctiveness. *Chasmosaurus russelli* and *C*. *belli* specimens represent subadult to adult, and adult specimens, respectively.

Our growth stages for *Chasmosaurus* compare well with those of Horner & Goodwin [39] for *Triceratops*, with similar proportional increases in skull size from one stage to the next. However, *Triceratops* attains a larger skull size, and undergoes element articulation (e.g., episquamosals and epiparietals) and remodeling of bone (e.g., reduced scalloping of squamosal lateral margin) earlier in ontogeny than does *Chasmosaurus*. Our *Chasmosaurus* growth series closely matches that of Mallon *et al*. [52], who used a smaller (n = 7) sample consisting of *Chasmosaurus* sp. and *C*. *belli* specimens, and a larger (n = 24) number of characters. In their study, ROM 843 was recovered as the most mature specimen, whereas in our analysis (Fig 8), CMN 2280, CMN 8800, TMP 1981.019.0175, and TMP 1983.025.0001 were all recovered as the most mature specimens. These latter four specimens were not included in Mallon *et al*. [52], as most of them (CMN 2280, CMN 8800, and TMP 1983.025.0001) have been previously referred to *C*. *russelli*.

### Comments on postorbital horncore development

Postorbital horncores have been shown to lengthen over ontogeny in centrosaurines (e.g., *Centrosaurus apertus* and *Coronosaurus brinkmani* and then resorbed in old age [38, 47]). Ontogenetic lengthening of postorbital horncores has been documented in some chasmosaurines (e.g., *Agujaceratops* [35]; *Pentaceratops sternbergii* [61]), but is best documented in *Triceratops horridus* [39]), whose horncores are stub-like early in ontogeny, but can become massive (>80 cm long) by adulthood [39]. This study confirms ontogenetic modification of the postorbital horncore in *Chasmosaurus*, although the pattern is not consistent across all specimens of each taxon.

**Fig 18:**
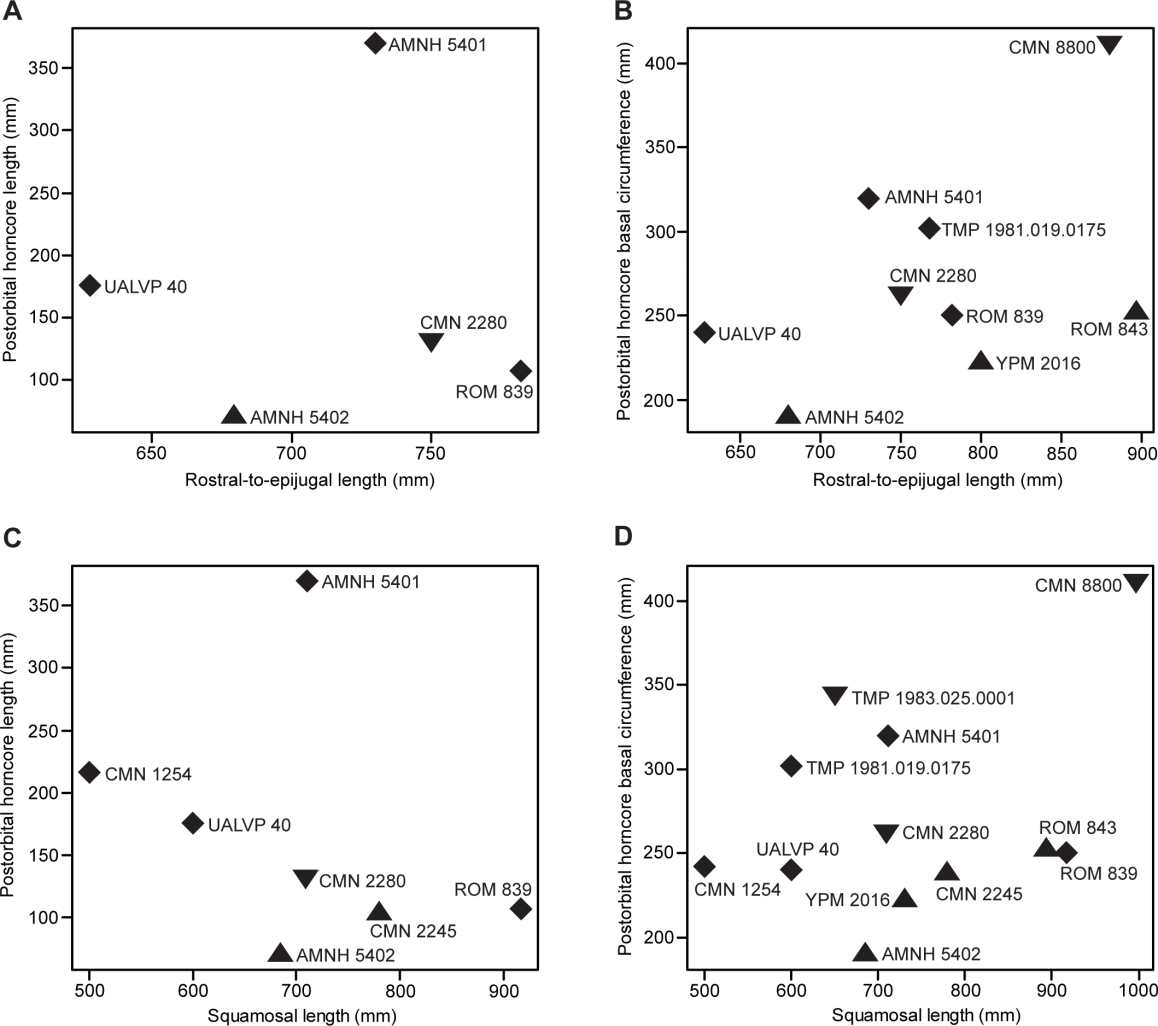
Scatter plots showing *Chasmosaurus* postorbital horncore size over skull size. Horncore length and basal circumference (parameters 1 and 4, Fig 2, respectively) used as proxies for horncore size; rostral-to-epijugal length and squamosal length (parameters 16 and 19, Fig 2, respectively) used as proxies for skull size. Rostral-to-epijugal length vs. horncore length (A) and basal circumference (B); and squamosal length vs. horncore length (C) and basal circumference (D). *Chasmosaurus belli* (triangles), *Chasmosaurus russelli* (inverted triangles), and *Chasmosaurus* sp. specimens (diamonds). [planned for page width].

**Fig 19:**
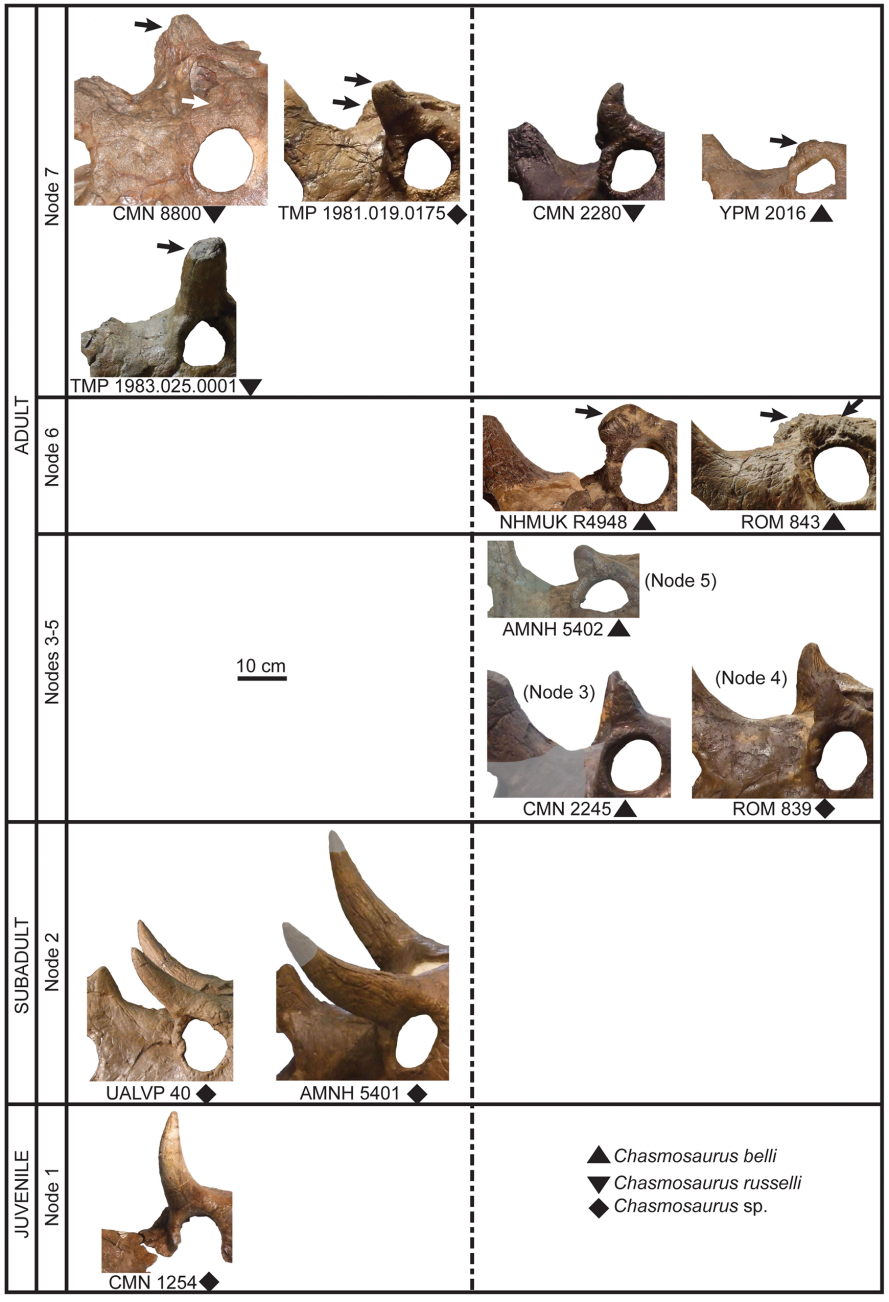
Ontogenetic development of postorbital horncores of Dinosaur Park Formation chasmosaurine skulls. Horncores arranged by ontogenetic stage; age classes and node numbers on left correspond to those given in the ontogenetic tree (Fig 8). Proposed “long horncore” (left) and “short horncore” (right) ontogenetic trajectories. Arrows = resorbed postorbital horncores; plaster reconstruction = grey. Image of ROM 843 courtesy of J. Mallon; image of NHMUK R4948 modified from Maidment & Barrett ([7]: Fig 4); images of CMN 1254, NHMUK R4948, ROM 839, and TMP 1981.019.0175 flipped. [planned for page width].

Ontogenetic changes in the size of the postorbital horncore (length and basal circumference–parameters 1 and 4, respectively, Fig 2) of *Chasmosaurus* were evaluated strictly on the basis of skull size (rostral-to-epijugal length and squamosal length–parameters 16 and 19, respectively, Fig 2), with larger skulls inferred as being more mature than smaller ones. The resulting discrepancy in horncore length and basal circumference amongst skulls of similar size and putatively similar growth stages (Fig 18) suggests that horncore size does not change consistently over ontogeny. This is supported by the age class distribution of horncores (Fig 19) in the ontogenetic analysis with the most mature specimens having a large discrepancy in size and shape.

Although horncore size is not positively allometric in *Chasmosaurus* (Figs 18 and 19), the *Triceratops* growth model suggests that variation in horncore length nevertheless is at least partly explained by ontogeny. This was recognized by Lehman [35], who suggested that the horncore of the relatively small skull of *Eoceratops canadensis* (CMN 1254; Fig 10A) could theoretically develop into the longer horncore of the larger skull of *Chasmosaurus kaiseni* (AMNH 5401; Figs 10G and 17B). He used this as evidence for their synonymy (i.e., *Chasmosaurus canadensis*). Konishi [9] supported Lehman’s [35] view, and suggested that such a transformation between the successively larger skulls UALVP 40 (Fig 17A), TMP 1979.011.0147, and AMNH 5401 was also possible. Konishi [9] postulated that *C*. *canadensis* may be valid, diagnosed by long horncores, and supported by the restriction of such specimens with known stratigraphy (i.e., TMP 1979.011.0147 and UALVP 40; Fig 4) to the lower DPF (Fig 4), but he refrained from formally resurrecting this taxon. We cannot support this interpretation because all of the specimens formally or informally referred to the long-horned *C*. *canadensis* lack the posterior parietal bar, making accurate taxonomic assignment very difficult, if not impossible.

Another skull, TMP 1983.025.0001 (Figs 3G and 19), has relatively long horncores and a relatively deep posterior parietal embayment, the latter of which is considered here to be diagnostic of *Chasmosaurus russelli*. The postorbital horncores of the *C*. *russelli* holotype (CMN 8800; Fig 17D) are extensively resorbed, but are inferred here to have been long, based on their large basal circumferences (Fig 18). The presence of long horncores on specimens with relatively deep posterior embayments in itself does not necessarily invalidate *C*. *canadensis*, nor does it mean that long horncores are instead diagnostic of *C*. *russelli*.

While Lehman [35] and Konishi’s [9] proposed ontogenetic trajectory may apply to some *Chasmosaurus* specimens (i.e., that some specimens developed relatively long horncores), not all specimens underwent such dramatic changes in horncore length over ontogeny (Figs 18 and 19). In general, specimens with deeper posterior parietal embayments (i.e., *C*. *russelli*) typically have longer postorbital horncores than those with more shallow embayments (i.e., *C*. *belli*) and fall within their assignments to *C*. *russelli* and *C*. *belli*, respectively. However, the discrepancy or potential dichotomy in horncore size (Fig 19) does not strictly correspond to specimens previously referred to *C*. *belli* and *C*. *russelli* (see CMN 2280, Fig 19), as first noted by Godfrey & Holmes [3]. Therefore, long and short postorbital horncore size are not robust diagnostic characters of *C*. *russelli* and *C*. *belli*, respectively, within *Chasmosaurus*.

Postorbital horncore orientation is also variable in *Chasmosaurus*, ranging from being upright and posteriorly curved (e.g., CMN 1254), to anterolaterally-directed and posteriorly curved (e.g., AMNH 5401, Fig 17B), to anterolaterally-directed in a sub-horizontal plane relative to the skull roof and straight (e.g., TMP 1979.011.0147). The ability to recognize orientation changes in the postorbital horncores of *Chasmosaurus* is partly hindered by the gradual resorption of these structures with age (Fig 19). However, based on the condition of intact and unmodified horncores, orientational variation of these structures does not appear to be ontogenetically integrated in *Chasmosaurus*, unlike *Triceratops* [39] and *Coronosaurus* [47]. Such variability in *Chasmosaurus* may represent a combination of ontogenetic, individual, and sexually dimorphic variation.

### Comments on *Kosmoceratops* sp. in the Dinosaur Park Formation

**Fig 20:**
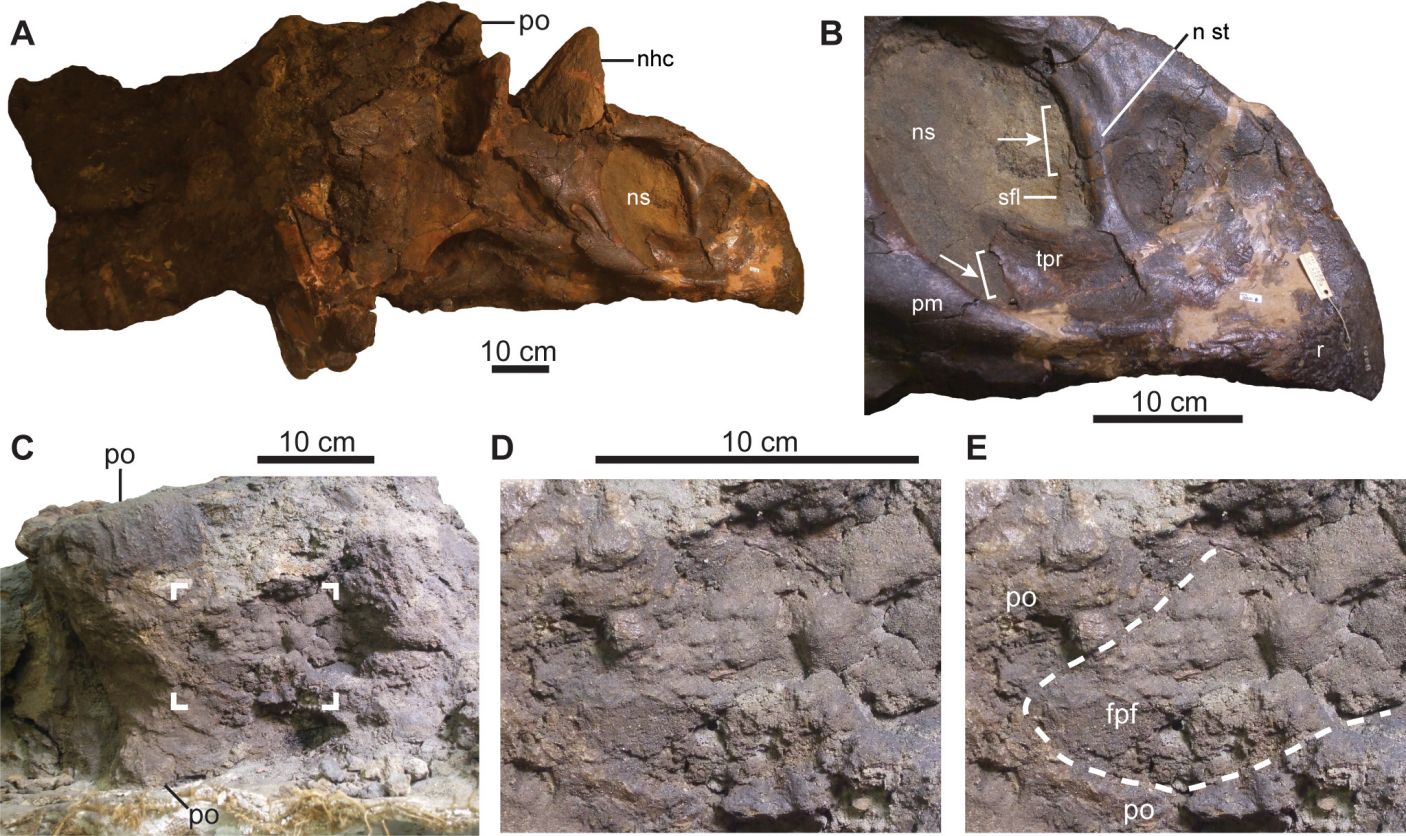
Skull of CMN 8801 (*Chasmosaurus* sp.). Skull in (A) right lateral view. Snout in (B) right lateral view; brackets and arrows delimit extent of breakage along the triangular process and septal flange of the premaxillae. Skull roof in (C) dorsal view; corner brackets delimit region shown in (D) and (E). Dashed line in ‘E’ represents the extent of the frontoparietal fontanelle, which is infilled with sediment. [planned for page width].

Longrich’s [8] reassignment of the *Chasmosaurus* skull CMN 8801 (Fig 20) to *Kosmoceratops* sp. was based on the following shared features: 1) relatively straight, weakly hooked rostral; 2) posteriorly inclined narial strut; 3) ventrally restricted septal flange; 4) triangular shaped, posterodorsally projecting triangular process; 5) posteriorly situated nasal horncore, relative to nares; and 6) roofed over frontoparietal fontanelle.

The shape of the rostral in CMN 8801 (Fig 20A and 20B) is within the range of *Chasmosaurus* (relatively straight in e.g., AMNH 5402, Fig 14B; concave in e.g., AMNH 5401) and is therefore likely attributable to individual, and not taxonomic, variation. The inclination of the narial strut is likely due to individual variation as well, as it is variable within *Chasmosaurus* (e.g., anteriorly inclined in UALVP 40, and posteriorly inclined in YPM 2016; Fig 14).

The septal flange of the premaxilla (Fig 13) in CMN 8801 is not restricted to the ventral half of the narial strut, but extends the full length of the strut, as in *Chasmosaurus*, although it is poorly preserved dorsally (Fig 20B, *contra* [8]). The triangular process of the premaxilla in CMN 8801 was probably square-shaped as in *Chasmosaurus* (Fig 20B), but is triangular shaped as preserved due to incomplete preservation of the posterior margin (*contra* [8]). The posteriorly situated nasal horncore, relative to the nares, of CMN 8801 is consistent with the degree of variability in *Chasmosaurus* (e.g., compare CMN 2280 with ROM 839, Fig 4), and is likely attributable to individual variation.

In some *Chasmosaurus* specimens (i.e., CMN 8800, Fig 17D; and YPM 2016), the frontoparietal fontanelle is partially covered by a bridge of bone formed by the postorbitals, but in other skulls, it remains open throughout ontogeny, unlike that of *Triceratops prorsus*, which secondarily closes with age [62]. In both TMP 1981.019.0175 (Fig 17C) and UALVP 40 (Figs 10C, 10D and 17A), transverse compression has distorted the fontanelle, such that its original shape cannot be reconstructed. Longrich [8] used the presence of a closed fontanelle to identify CMN 8801 as *Kosmoceratops*, a chasmosaurine previously thought to be restricted to southern Laramidia [5], but re-examination of the specimen reveals that the fontanelle is open, but filled with matrix (Fig 20C–20E). The restriction of *Kosmoceratops* to a relatively narrow latitudinal zone (Utah) is consistent with the distribution of other Campanian ceratopsids of Laramidia, which appear to have been highly endemic [5, 63]. Although Campanian sediments of Laramidia have generally been well sampled, further prospecting in these deposits will likely expand the known geographic range of ceratopsid taxa.

### Comments on *Vagaceratops*

*Vagaceratops irvinensis* was originally considered by Holmes *et al*. [19] to be a species of *Chasmosaurus*, as their phylogenetic analysis recovered the former taxon as being nested within the latter. However, the less-resolved, polytomic relationship between *Chasmosaurus* and *Vagaceratops* in our study (Fig 5) does not unequivocally support their stance. Instead, we tentatively consider the frill morphology and ornamentation of *V*. *irvinensis* to be sufficiently distinct from *Chasmosaurus* to merit its own genus as designated by Sampson *et al*. [5]. Future discoveries of chasmosaurine material will test this hypothesis. The inclusion of AMNH 5402 and YPM 2016 in a ‘*Vagaceratops*-like’ clade (AMNH 5402 + (YPM 2016 + (CMN 41357 + TMP 1987.045.0001))), is, however, unique to this study.

**Fig 21:**
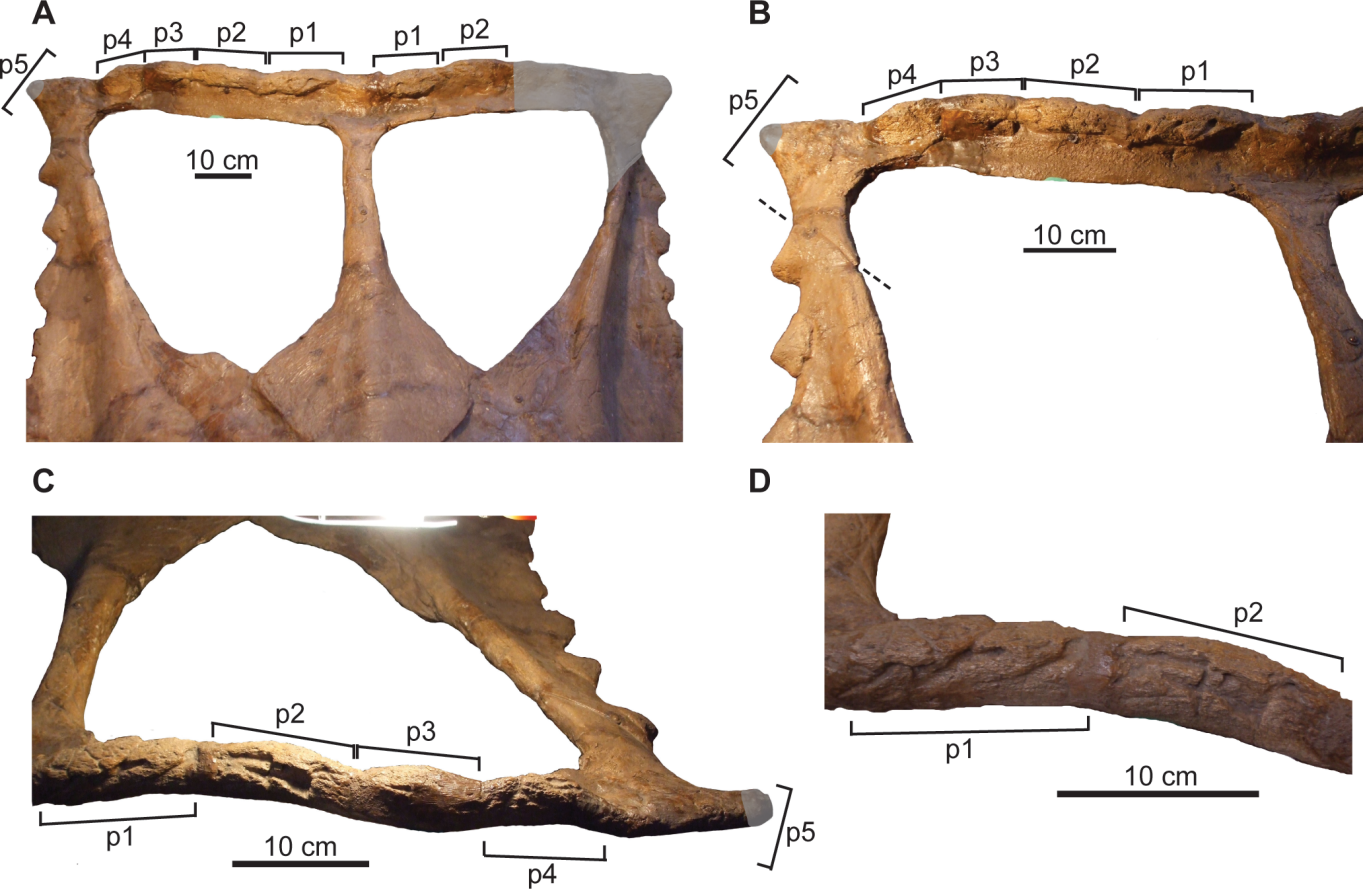
Frill of YPM 2016 (*Chasmosaurus belli*). Frill in: (A) dorsal view, (B) dorsal view of right half, (C) posterior view of right posterior parietal bar, and (D) posterior view of medial portion of right posterior bar. Brackets delimit size of epiossifications; dashed line = parietal-squamosal contact; plaster reconstruction = grey. [planned for page width].

YPM 2016 (Fig 21) is unusual in its possession of five previously unidentified epiparietals on each side of the posterior parietal bar, whereas other *Chasmosaurus* skulls have, or are assumed to have, only three per side (Fig 3). YPM 2016 is similar to *Vagaceratops* (CMN 41357 and TMP 1987.045.0001) in their possession of a straight posterior parietal margin (inferred for TMP 1987.045.0001, Fig 3Q, but coded as “?” in phylogenetic analysis), four anterodorsally oriented epiparietals on each side (P1–4; inferred for TMP 1987.045.0001, Fig 3Q), and a fifth epiossification oriented in the plane of the frill, either articulated with the parietal (P5; CMN 41357, Fig 3R; and YPM 2016, Fig 3M) or straddling the parietal-squamosal contact (epiparietosquamosal; TMP 1987.045.0001, Fig 3Q). However, YPM 2016 is distinct from CMN 41357 and TMP 1987.045.0001 in that its four medialmost epiparietals are greatly reduced in length and are not coalesced at their bases (Fig 21). The epiparietals of the latter specimens are also curved more anteriorly and overlie the posterior parietal bar (Fig 3Q and 3R). YPM 2016 also has a significantly larger parietal fenestra length/width ratio (1.03; Fig 3M) compared with TMP 1987.045.0001 (0.87; Fig 3Q) and CMN 41357 (0.72; Fig 3R).

**Fig 22:**
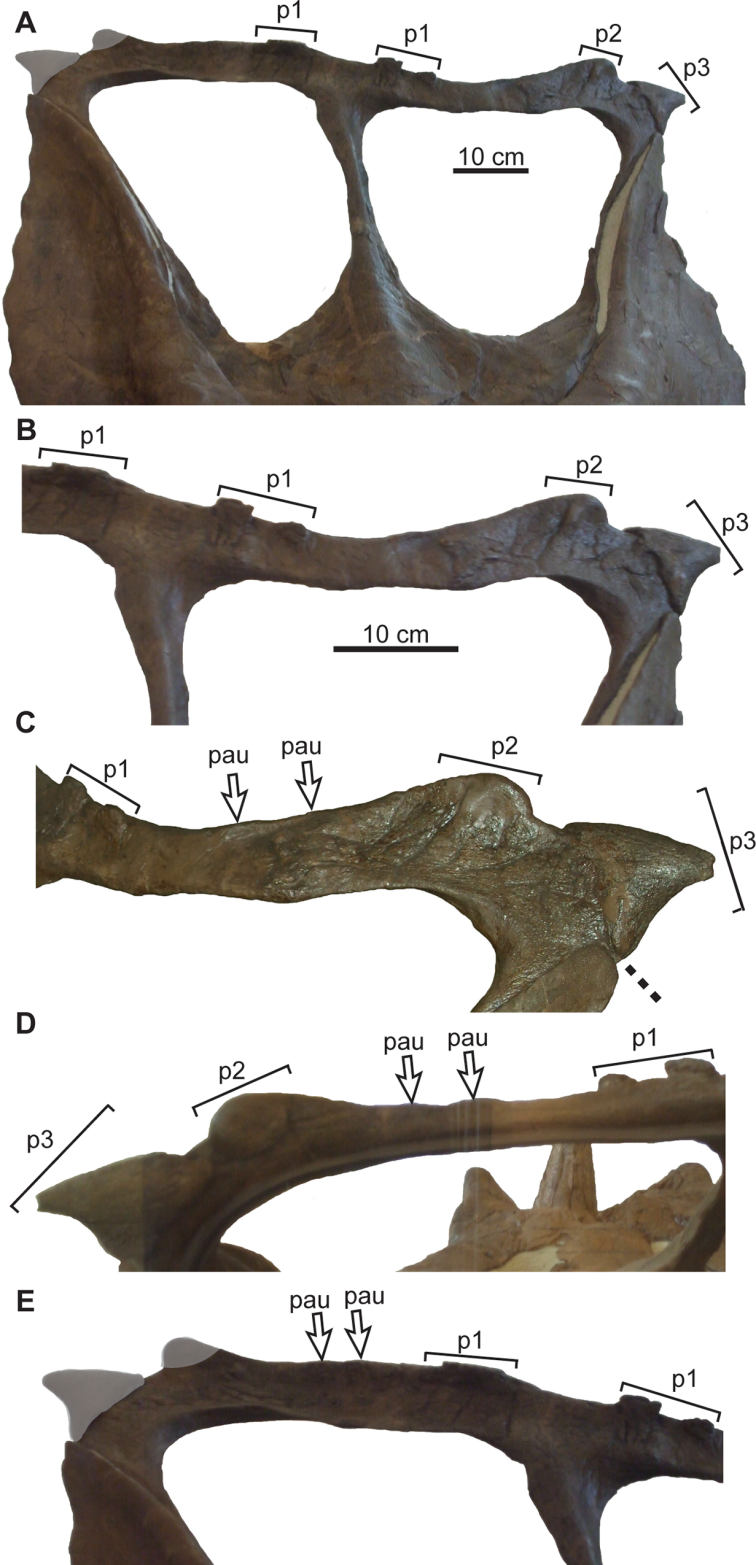
Frill of AMNH 5402 (*Chasmosaurus belli*). Frill in: (A) dorsal, (B) dorsal (left side), (C) oblique dorsal (left side), (D) posterior (left side), and (E) dorsal (right side) views. Brackets delimit size of epiossifications; dashed line = parietal-squamosal contact; arrows = inferred epiparietal loci; plaster reconstruction = grey. [planned for column width].

In AMNH 5402 (Fig 22), the right squamosal has been distorted mediolaterally and pushed posteriorly, distorting the posterior bar such that the right lateral corner extends farther posteriorly than the left corner. The undistorted right posterior bar indicates that, like YPM 2016 and *Vagaceratops*, the posterior bar of AMNH 5402 was straight. AMNH 5402 differs from *Vagaceratops* in that it has only three epiparietals per side (Fig 22), although these epiparietals are unusually arranged in this specimen. The left P1 is missing its central portion, exposing part of the low-relief locus to which it is articulated; the right P1 is complete. The gap between P1 and P2 is significantly larger than between P2 and P3; in other skulls, the spacing between epiparietals is nearly uniform (Fig 3). Two low-relief parietal undulations are separated from each other and from the two neighbouring epiparietals by approximately equal intervals. They possess shallow, transverse grooves in dorsal view, making them dorsoventrally pinched. The preserved left P3 partially overhangs, but is not fused to, the posterior end of the squamosal. The parietal fenestra length/width ratio of AMNH 5402 is also large (1.04) relative to *Vagaceratops*, but small relative to other *Chasmosaurus* specimens (AMNH 5656 = 1.89 to 1.09 = ROM 843). Although AMNH 5402 and YPM 2016 possess some features common to *Vagaceratops*, we will address the nature of these similarities in a separate publication. Until then, however, we tentatively attribute these differences to individual variation within *C*. *belli*.

This study revisited the null hypothesis of one species of *Chasmosaurus*, first rigorously tested by Godfrey & Holmes [3]. Since the time of their study, new chasmosaurine specimens have been collected, prepared, and described, and detailed stratigraphic work for several historic specimens has been conducted. This work has facilitated the analyses performed in this study, which tested the source of morphological variation within *Chasmosaurus*. By placing specimens in ontogenetic and stratigraphic sequences, some of this variation can be accounted for. Amongst the specimens examined in this study, we recognize three valid chasmosaurine taxa: *Chasmosaurus belli*, *Chasmosaurus russelli*, and *Vagaceratops irvinensis* (*Mercuriceratops gemini* [18] and *Pentaceratops aquilonius* [8] were not analyzed in this study). Other previously named chasmosaurine taxa from the DPF are either a junior synonym of one of the above valid taxa (i.e., *Mojoceratops perifania* a junior synonym of *C*. *russelli*), insufficiently preserved to confirm its validity (*Chasmosaurus canadensis*), a junior synonym of the latter (*Chasmosaurus kaiseni*), non-diagnostic (*Chasmosaurus brevirostris*), or not present in the DPF (*Kosmoceratops*).

SYSTEMATIC PALAEONTOLOGY

CERATOPSIA [64]

NEOCERATOPSIA [65]

CERATOPSIDAE [66]

CHASMOSAURINAE [67]

*CHASMOSAURUS* [2]

**Emended diagnosis (modified from Konishi [9])**—*Chasmosaurus* is diagnosed based on the following unique combination of characters: (1) Premaxillary flange along entire anterior margin of external naris; (2) postorbital horncores, when present, curve posteriorly along their length; (3) squamosal dorsal border laterally adjacent to dorsal temporal fenestra straight in profile, anteriorly at level with base of postorbital horncore, and sloping posteroventrally at a shallow angle before ascending farther posteriorly to form lateral border of parietal fenestra; (4) medial margin of squamosal, where it articulates with the lateral bar of the parietal, straight; (5) frill broadens posteriorly to form rectangular to triangular shield with maximum width more than twice the skull width at orbits; (6) parietal fenestrae large, occupying most of the parietal, and being rounded or anteroposteriorly longer than transversely wide; and (7) epiparietals straight and triangular in shape and oriented posteriorly or anterodorsally.

**Type species**—*Chasmosaurus belli* [2]

**Specific diagnosis**—Medial margin of posterior parietal bar straight (right and left halves of the bar meeting at a 180° angle at the midline) or shallowly embayed with the right and left halves meeting at an angle of not less than 136°.

**Distribution**—Lower to middle beds of the Dinosaur Park Formation (DPF; Dinosaur Park faunal zones (DPFZs) 1 to 2 of [15]) of Alberta (Dinosaur Provincial Park (DPP)), Canada.

**Synonymies**—*Monoclonius belli* [1]; *Ceratops belli* [68]; *Protorosaurus belli* [69].

**Type specimen**—CMN 0491, a partial parietal. Although fragmentary, the holotype is diagnostic based on the combination of generic characters 6 and 7, and specific character 1 (shallow posterior embayment), a combination not observed in any other chasmosaurine. The width of the parietal fenestrae (character 6) in this specimen, however, cannot be determined.

**Assigned specimens**—AMNH 5402, CMN 2245, NHMUK R4948, ROM 843, and YPM 2016.

*Chasmosaurus russelli* [70]

**Specific diagnosis—**Medial margin of posterior parietal bar moderately to deeply embayed, with the two halves of the bar forming an angle of between 89° and 128° at the midline.

**Distribution**—Lower to upper beds of the DPF (DPFZs 1 to 3 of [15]) of Alberta (DPP, Hilda, Manyberries, Onefour) and Saskatchewan (Saskatchewan Landing Provincial Park), Canada.

**Synonymies**—*Mojoceratops perifania* [6].

**Type specimen**—CMN 8800, a mostly complete skull lacking the lower jaw and part of the rostral, part of the jugals from both sides, part of the right quadrate, squamosal, and parietal.

**Assigned specimens**—AMNH 5656, CMN 2280, CMN 8800, CMN 8803, CMN 41933, TMP 1983.025.0001, TMP 1997.132.0002, and TMP 1999.055.0292.

*Chasmosaurus* sp.

The following specimens are referable to *Chasmosaurus*, but cannot be assigned to species as they do not preserve the diagnostic medial margin of the posterior parietal bar: AMNH 5401 (holotype: *Chasmosaurus kaiseni* [57]), CMN 1254 (holotype: *Monoclonius canadensis* [1]; *Ceratops canadensis* [68]; *Eoceratops canadensis* [67]; *Chasmosaurus canadensis* [35]), CMN 8801, CMN 8802, CMN 34829, CMN 34832, CMN 41933, ROM 839 (holotype: *Chasmosaurus brevirostris* [59], TMP 1979.011.0147, TMP 1981.019.0175, TMP 1993.082.0001, and UALVP 40.

**Distribution**—*Chasmosaurus* sp. specimens with known stratigraphy were collected from DPFZs 1 (CMN 8801, TMP 1979.011.0147, TMP 1981.019.0175, UALVP 40), 2 (ROM 839), and 3 (TMP 1993.082.0001). CMN 8802 was collected from the uppermost Oldman Formation of southern Alberta (Milk River region), directly below the Lethbridge Coal Zone, and is age-equivalent to DPFZ 3; all other *Chasmosaurus* sp. specimens were collected from DPP.

## Supporting Information

S1 File Dinosaur Park Formation chasmosaurine data. (XLSX)

S2 File Phylogenetic character list. (DOC)

S3 File Phylogenetic character-taxon matrix (TNT file). (TNT)

S4 File Phylogenetic character-taxon matrix (NEXUS file). (NEX)

S5 File Ontogenetically variable phylogenetic characters. (DOC)

S6 File Cranial parameters. (XLSX)

S7 File Ontogenetic character list. (DOC)

S8 File Ontogenetic character-specimen matrix (TNT file). (TNT)

S9 File Ontogenetic character-specimen matrix (NEXUS file). (NEX)

S10 File Supporting cranial morphometric data. (DOC)
