# Supplementary material for: A Re-Evaluation of the Chasmosaurine Ceratopsid Genus Chasmosaurus (Dinosauria: Ornithischia) from the Upper Cretaceous (Campanian) Dinosaur Park Formation of Western Canada
Source: PLoS One. 2016 Jan 4;11(1):e0145805. doi: 10.1371/journal.pone.0145805 (PMC4699738; doi:10.1371/journal.pone.0145805)
Supplement: S2 File — (DOC) [file pone.0145805.s002.doc]

**Supplementary Material 2. List of characters used in phylogenetic analysis.**

**Anatomical terminology modifications**
 For the sake of using consistent terminology, the following descriptive changes were made to the following characters: “supraorbital” changed to “postorbital” (35–41); “laterotemporal fenestra” (42 and 52) changed to “lateral temporal fenestra”; “infratemporal process” changed to “lateral temporal process” (47); “dorsotemporal fenestra” (54, 62, and 74) changed to “dorsal temporal fenestra”; “frontal fontanelle” (55 and 56) changed to “frontoparietal fontanelle”; “transverse parietal bar” (67, 76, and 77) and “caudal ramus of parietal” (68) changed to “posterior parietal bar”; and “rostral or rostro-“ changed to “anterior or antero-“ and “caudal or caudo-“ changed to “posterior or postero-“ (1, 4, 7, 9, 17–19, 24, 25, 29, 32, 34, 37, 38, 40, 41, 46, 52, 57, 62, 66, 68, 70, 72, 76, 77, 86, 98, 103, 111, 118, 119, 128, 142, 152, and 154). The above replaced terms are well established in the literature, but were changed to be more consistent with the terminology used in the rest of this study.
 Epiparietals are numbered sequentially starting at the midline of the posterior parietal and progress towards the squamosal (Sampson *et al.*, 2010). Although episquamosals have been traditionally numbered sequentially starting at the anterior end of the squamosal, we have chosen to follow the methodology of Sampson *et al.* (2010), in which episquamosals are numbered sequentially starting at the posterior end of the squamosal.

**List of characters**
 Taxa scored differently from Campbell (2015) are shown below, organized by characters; rescored codings for *Bravoceratops polyphemus* are also given, modified from Wick & Lehman (2013). Some characters in this study describe the presence or absence of a feature, and are followed by other characters that describe that feature in species or specimens that possess that feature. Species or specimens coded as ‘absent’ for the former characters were coded as ‘?’ in the latter characters. Species or specimens in which a given feature is not preserved were also coded as’?’ for characters relating to that feature. Therefore, species or specimens in which a given feature is either absent or not preserved were coded as “?”.
 It could be argued that characters 18, 22, 84, 114, and 116 are not independent of other characters (19, 21, 82, 113, and 115, respectively); however, these characters were not excluded from the analysis as they have been used in previous phylogenetic studies (e.g. Forster *et al.*, 1993; Sampson *et al*., 2010; Mallon *et al.*, 2011; Wick & Lehman, 2013). For each of the 155 character descriptions, the terms “outgroup”, “centrosaurine”, “chasmosaurine”, “ceratopsid”, and “neoceratopsian” taxa refer strictly to the relevant taxa included in the analysis, and not necessarily to these groups in their entirety.

Dermal skull roof

(1) Rostral, extent of dorsal and ventral processes (Dodson *et al.*, 2004, character 1):
 (0) – triangular in lateral view, with short dorsal and ventral processes.
 (1) – elongate, with deeply concave posterior margin and hypertrophied dorsal and
 ventral processes.
 The dorsal and ventral processes of the rostral (Fig. 13) are elongate in
 *Leptoceratops*, *Zuniceratops*, and chasmosaurines. These processes are short in
 *Protoceratops* and in centrosaurines.

(2) Nares, size and position (Sampson *et al.*, 2010, character 2):
 (0) – small, restricted to dorsal 1/3 of premaxilla, undifferentiated, 10% or less that of
 basal skull length.
 (1) – large, expanded to occupy most of the depth of the premaxilla, 15% or greater than
 basal skull length.
 The naris is relatively large in *Zuniceratops*, and in ceratopsids. The naris is
 relatively small in *Leptoceratops* and *Protoceratops*.

(3) Premaxillary septum (Chinnery & Weishampel, 1998, character 10):
 (0) – absent.
 (1) – present.
 The premaxillary septum is a relatively thin, medial wall of bone formed by both
 premaxillae (Fig. 13). This septum is present in *Zuniceratops* and in ceratopsids. The
 septum is absent in *Leptoceratops* and *Protoceratops*.

(4) Premaxillary septum, shape (Dodson *et al.*, 2004, character 4):
 (0) – anteriorly elongate.
 (1) – hemicircular.
 The premaxillary septum is anteriorly elongate in *Zuniceratops* and in
 chasmosaurines, but hemicircular in centrosaurines.

(5) Premaxillary septum, nasal contribution (Sampson *et al.*, 2010, character 5):
 (0) – septum formed by premaxilla only.
 (1) – septum formed by premaxilla and nasal.
 The premaxillary septum is formed only by the premaxilla in *Zuniceratops* and in
 chasmosaurines. The septum is formed by the premaxilla and nasal in centrosaurines.

(6) Premaxilla, narial strut (Holmes *et al.*, 2001, character 1):
 (0) – absent.
 (1) – present.
 The narial strut is a narrow strut situated towards the posterior margin of the
 premaxillary septum (Fig. 13), and is sub-vertically oriented. This strut is present in
 chasmosaurines, but absent in centrosaurines and outgroup taxa.

(7) Premaxilla, narial strut orientation (Dodson *et al.*, 2004, character 6):
 (0) – anteriorly inclined.
 (1) – posteriorly inclined.
 The narial strut of the premaxilla is anteriorly inclined in most *Chasmosaurus*
 specimens (except for CMN 8801 and YPM 2016) and other chasmosaurines, but is
 posteriorly inclined in *Kosmoceratops*, *Coahuilaceratops*, *Anchiceratops*, and
 *Arrhinoceratops*.

(8) Premaxilla, septal flange (Holmes *et al.*, 2001, character 2):
 (0) – absent.
 (1) – present.
 The septal flange is a thin flange of bone on the posterior margin of the
 premaxillary septum, and projects towards the naris (Fig. 13). This flange is present in
 *Chasmosaurus*, *Vagaceratops*, *Kosmoceratops*, *Agujaceratops*, *Utahceratops*,
 *Pentaceratops*, and dimorphic for *Anchiceratops*. The flange is absent in other
 chasmosaurines and in centrosaurines.
 *Centrosaurus apertus* and *Pachyrhinosaurus lakustai* were both recoded from ? to
 0 because the premaxilla septal flange is absent.

(9) Premaxilla, septal flange length (Forster *et al.*, 1993, character 1):
 (0) – spans entire posterior margin of narial strut.
 (1) – restricted to ventral portion of narial strut.
 The septal flange of the premaxilla spans the entire posterior margin of the narial
 strut in *Chasmosaurus*, *Vagaceratops* and *Agujaceratops*. The flange is restricted to the
 ventral portion of the narial strut in *Kosmoceratops*, *Utahceratops*, *Pentaceratops*, and
 *Anchiceratops*.

(10) Premaxilla, septal fossa (Holmes *et al.*, 2001, character 4):
 (0) – absent.
 (1) – present.
 The septal fossa is a subcircular fossa that indents the premaxillary septum (Fig.
 13). This fossa is present in chasmosaurines, but absent in centrosaurines and outgroup
 taxa.

(11) Premaxilla, subordinate fossa in premaxillary septum (Dodson *et al.*, 2004, character 8,
 modified here)
 (0) – absent.
 (1) – present.
 This character originally read “interpremaxillary fossa”, but was changed to
 “subordinate fossa”. The “interpremaxillary fossa” of Dodson *et al.* (2004) and “septal
 fossa” of Holmes *et al.* (2001; and character 10 in this study) both refer to the same
 feature; the “premaxillary fossa” of Forster (1996) also refers to the same feature.
 Sampson *et al.* (2010) and Mallon *et al.* (2011), however, used the term
 “interpremaxillary fossa” to refer to the smaller fossa situated anterior to the septal fossa
 (Fig. 13). Lehman (1998: 902) refers to this smaller fossa as the “subordinate fossa” of
 the premaxilla. The term “subordinate fossa” was used in this study to avoid confusion,
 as its name indicates that it is subordinate in size compared to the septal fossa. The
 subordinate fossa is present in *Agujaceratops*, *Anchiceratops*, *Pentaceratops*,
 *Eotriceratops*, *Nedoceratops*, *Torosaurus latus*, and both species of *Triceratops*.
 *Agujaceratops mariscalensis* and *Pentaceratops sternbergi* were both recoded
 from 0 to 1 because the subordinate fossa of the premaxilla is present.

(12) Premaxilla, accessory strut in septal fossa (Sampson *et al.*, 2010, character 12):
 (0) – no accessory strut.
 (1) – strut present.
 This accessory strut is situated in the septal fossa of the premaxilla, anterior to the
 narial strut (Fig. 13). This accessory strut is absent in *Chasmosaurus*, *Vagaceratops*,
 *Kosmoceratops*, *Agujaceratops*, *Anchiceratops*, *Arrhinoceratops*, and *Eotriceratops*. The
 accessory strut is present in other chasmosaurines.

(13) Premaxilla, triangular process (Forster, 1990, character 21):
 (0) – absent.
 (1) – present .
 The triangular process is a process on the posteroventral margin of the
 premaxillary septum, and it projects into the naris (Fig. 13). This process is present in
 chasmosaurines, but absent in centrosaurines and outgroup taxa.

(14) Premaxilla, triangular process shape (Sampson *et al.*, 2010, character 14):
 (0) – square.
 (1) – pinched and triangular with concave facets.
 The triangular process of the premaxilla is square in *Chasmosaurus*,
 *Vagaceratops*, *Agujaceratops*, and *Arrhinoceratops*, but triangular in other
 chasmosaurines.

(15) Premaxilla, triangular process recess (Dodson *et al.*, 2004, character 12):
 (0) – absent.
 (1) – present.
 This recess is a pronounced concave surface on the lateral margin of the triangular
 process of the premaxilla (Fig. 13). This recess is absent in chasmosaurines, except for
 *Nedoceratops* and both species of *Triceratops*.

(16) Premaxilla, recess along ventral portion of septum (Dodson *et al.*, 2004, character 9):
 (0) – absent.
 (1) – present.
 The recess along the ventral portion of the premaxillary septum is absent in
 chasmosaurines and outgroup taxa, but present in centrosaurines (Fig. 13).

(17) Premaxilla, posteroventral expansion of oral margin (Forster, 1990, character 6):
 (0) – absent.
 (1) – present.
 The oral margin of the premaxilla is not posteroventrally expanded in
 chasmosaurines, but is expanded in centrosaurines and outgroup taxa (Fig. 13).

(18) Premaxilla, position of posterior tip of posteroventral process (Forster *et al.*, 1993, character
 7):
 (0) – inserts into an embayment in the nasal.
 (1) – intervenes between nasal and maxilla.
 Character 18 does not appear to be independent of 19. The posterior tip of the
 posteroventral process of the premaxilla inserts between the nasal and maxilla in
 ceratopsids. Of the ceratopsids that possess a forked posterior tip, the upper part of the
 fork inserts into an embayment in the nasal. Therefore, 18(0) always pairs with 19(1), and
 18(1) always pairs with 19(0) (Andrew Farke, pers. comm.).

(19) Premaxilla, distal end of posteroventral process forked (Forster, 1990, character 14; and
 Forster *et al.*, 1993, character 7, modified here):
 (0) – absent.
 (1) – present, with upper half of fork inserting into an embayment in the nasal.
 “With upper half of fork inserting into an embayment in the nasal” was
 added to state (1). This was done to accommodate pairing with character 18 (Andrew
 Farke, pers. comm.). The process is forked in *Zuniceratops*, *Chasmosaurus*,
 *Vagaceratops*, *Kosmoceratops*, *Agujaceratops*, *Utahceratops*, and *Pentaceratops* (Fig.
 13). The fork is absent in *Leptoceratops*, *Protoceratops*, centrosaurines, and other
 chasmosaurines.

(20) Premaxilla-nasal contact in dorsal view (Sampson *et al.*, 2010, character 20):
 (0) – premaxillae insert between nasal.
 (1) – nasals insert between premaxillae.
 The nasals insert between the premaxillae in dorsal view in *Agujaceratops*,
 *Utahceratops*, *Pentaceratops*, and *Coahuilaceratops*. In other chasmosaurines,
 centrosaurines and outgroup taxa, the premaxillae insert between the nasals (Fig. 13).

(21) Accessory antorbital fenestra (Forster, 1990, character 15):
 (0) – present.
 (1) – absent.
 The accessory antorbital fenestra is a perforation situated anterior to the antorbital
 fenestra, at the junction between the premaxilla, maxilla and nasal. This feature was
 scored as present only in taxa possessing a large, distinct perforation. Taxa possessing
 either a small and indistinct perforation or no perforation were scored as absent. This was
 done because a small perforation could actually represent breakage, or its apparent
 absence could be attributed to plaster reconstruction. This fenestra is absent in
 *Chasmosaurus*, except for AMNH 5401 and CMN 8800. The fenestra is present in
 *Zuniceratops*, and in the chasmosaurines *Kosmoceratops*, *Utahceratops* and
 *Pentaceratops* (absent in other chasmosaurines); this fenestra is absent in centrosaurines
 (except for *Diabloceratops* – not included in this analysis).
 *Protoceratops andrewsi* was recoded from 0 to 1 because it lacks a large, distinct
 accessory antorbital fenestra.

(22) Accessory antorbital fenestra size (Sampson *et al.*, 2010, character 22):
 (0) – pronounced, penetration of nasal cavity visible in lateral view.
 (1) – slight penetration, nasal cavity not visible in lateral view.
 Character 22 does not appear to be independent of 21. In taxa possessing a large
 and distinct accessory antorbital fenestra (21(0) – *Zuniceratops*, *Kosmoceratops*,
 *Utahceratops*, *Pentaceratops*, and the *Chasmosaurus* specimens AMNH 5401 and CMN
 8800), the nasal cavity is visible in lateral view (22(0)). Therefore, 21(0) always pairs
 with 22(0).
 *Kosmoceratops richardsoni* was recoded from 1 to 0, *Utahceratops gettyi* ? to 0,
 and *Pentaceratops sternbergi* 1 to 0 because the nasal cavity is visible through the
 accessory antorbital fenestra.

(23) External antorbital fossa, size (Forster, 1990, character 44):
 (0) – large, 20% or more length of body of maxilla.
 (1) – greatly reduced or absent, less than 10% length of body of maxilla.
 The external antorbital fossa is a shallow, funnel-like depression on the side of the
 snout that leads into the antorbital fenestra. This fossa is relatively large in outgroup taxa,
 but relatively small in ceratopsids.
 *Turanoceratops tardabilis* was recoded from 0 to ? because the maxilla is
 fragmentary and does not preserve the entire external antorbital fossa.

(24) Maxilla, relation of alveolar margin to anterior edentulous margin (Sampson *et al.*, 2010,
 character 24):
 (0) – edentulous portion maxilla elevated above level of alveoli.
 (1) – at same level.
 The alveolar and edentulous margins of the maxilla are at the same level in
 chasmosaurines, *Centrosaurus* and *Pachyrhinosaurus*. The edentulous margin is above
 the alveolar margin in *Albertaceratops* and outgroup taxa.

(25) Maxilla and premaxilla, diastema on anterior maxilla (Sampson *et al.*, 2010, character 25,
 modified here):
 (0) – present.
 (1) – absent.
 This character was changed from “maxilla” to “maxilla and premaxilla”, as it
 pertains to both elements. The anterior end of the maxilla is edentulous in ceratopsians.
 However, premaxillary teeth are present in some taxa, making the edentulous part of the
 maxilla a diastema (Andrew Farke, pers. comm.). This diastema is present in
 *Protoceratops*, but absent in *Leptoceratops*, *Zuniceratops* and ceratopsids.
 *Leptoceratops gracilis* and *Zuniceratops christopheri* were both recoded from 0 to
 1 because they lack premaxillary teeth, and therefore lack a diastema between the
 premaxilla and maxilla. *Turanoceratops tardabilis* was recoded from 0 to ? because the
 premaxilla is not preserved, and the presence of a diastema cannot be determined.

(26) Maxilla, maxillary cavity (Sampson *et al.*, character 26):
 (0) – absent.
 (1) – present.
 The maxillary cavity is a large cavity on the medial margin of the maxilla. This
 cavity is present in ceratopsids, but absent in outgroup taxa.

(27) Nasal, ornamentation in adult (Sampson *et al.*, 2010, character 27):
 (0) – absent.
 (1) – present.
 Nasal ornamentation is present in *Protoceratops* and ceratopsids, but absent in
 *Leptoceratops* and *Zuniceratops*.

(28) Nasal, ornamentation type in adult (Sampson *et al.*, 2010, character 28, modified here):
 (0) – non-pronounced.
 (1) – distinct and/or forming a horncore.
 (2) – pachyostotic boss.
 State (1) was modified from “distinct horncore” to “distinct and/or forming a
 horncore”, as *Albertaceratops* (and *Medusaceratops* – not included in this analysis) have
 pronounced nasal ornamentation but not quite a horncore. The nasals form a horncore in
 chasmosaurines and in most centrosaurines except for *Pachyrhinosaurus* (pachyostotic
 boss). The nasal ornamentation is non-pronounced in *Protoceratops*.
 *Albertaceratops nesmoi* was recoded from 0 to 1 because it has pronounced nasal
 ornamentation (although it is not technically a horncore).

(29) Nasal, ornamentation position, measured perpendicular to horizontal toothrow (Sampson *et
 al.*, 2010, character 29):
 (0) – centred dorsal to or posterior to centre of endonaris.
 (1) – centred anterior to centre of endonaris.
 Nasal ornamentation is centered dorsal to or posterior to the centre of the
 endonaris in *Protoceratops*, centrosaurines, and chasmosaurines except for
 *Coahuilaceratops*, *Ojoceratops*, *Torosaurus*, *Nedoceratops*, *Triceratops*, and
 *Bravoceratops*.

(30) Nasal, narial spine (Forster, 1990, character 22):
 (0) – absent.
 (1) – present.
 The narial spine of the nasal is a finger-like process on the rim of the naris that
 projects into the narial opening (Fig. 13). This spine of the nasal is present in
 centrosaurines, but absent in chasmosaurines and outgroup taxa.

(31) Posterior end of maxillary alveolar process, visibility in lateral view (Sampson *et al.*, 2010,
 character 31, modified here):
 (0) – deep, alveolar process of maxilla entirely visible.
 (1) – shallow, alveolar process of maxilla obscured by jugal.
 This character phrasing replaces the original “facial skeleton, dorsoventral depth
 in orbital region”, as the former better explains this character. The jugal obscures the
 alveolar process of the maxilla in lateral view in *Chasmosaurus* and *Vagaceratops*. In
 other chasmosaurines, centrosaurines and outgroup taxa, the alveolar process is entirely
 visible in lateral view.

(32) Orbit, orientation (Sampson *et al.*, 2010, character 32):
 (0) – directed anterolaterally.
 (1) – directed laterally.
 The orbit is laterally-oriented in ceratopsids and *Zuniceratops*, but anterolaterally-
 oriented in *Leptoceratops* and *Protoceratops*.

(33) Orbit diameter (Sampson *et al.*, 2010, character 33, modified here):
 (0) – more than 20% of basal skull length.
 (1) – less than 15% of basal skull length.
 In character 33, “skull length” actually refers to basal skull length (Andrew Farke,
 pers. comm.); the change has been made here. Basal skull length is defined as the
 distance between the rostral and occipital condyle, as measured along the midline. The
 orbit diameter is relatively small in ceratopsids and *Zuniceratops* but relatively large in
 *Leptoceratops* and *Protoceratops*.
 *Turanoceratops tardabilis* was recoded from 0 to ? because the orbit is not
 preserved.

(34) Lacrimal size (Chinnery & Weishampel, 1998, character 1):
 (0) – large, forms 50% or more of the anterior orbital margin.
 (1) – small, forms 40% or less of the anterior orbital margin.
 The lacrimal is relatively small in ceratopsids and *Zuniceratops*, but relatively
 large in *Leptoceratops* and *Protoceratops*.

(35) Postorbital, postorbital ornamentation in adult (Sampson *et al.*, 2010, character 35):
 (0) – absent.
 (1) – present.
 Postorbital ornamentation is present in ceratopsids, *Zuniceratops* and
 *Turanoceratops*, but absent in *Leptoceratops* and *Protoceratops*. *Vagaceratops* (CMN
 41357 and TMP 1987.045.0001) were scored as “?”, because they possess a low-relief
 mound above each orbit. These mounds are difficult to interpret, and it cannot be
 determined whether they represent the bases of longer horncores removed by bone
 resorption, or if horncores were never present in life. A well-developed pit is present in
 the preserved right mound of CMN 41357 (*Vagaceratops*), which may suggest that this
 individual had horncores in life.

(36) Postorbital, extent of cornual sinuses in base of postorbital ornamentation (Sampson *et al.*,
 2010, character 36):
 (0) – sinus space invades frontal and parietal.
 (1) – sinus space enters postorbital.
 The cornual sinus is a large cavity in the dorsal roof of the skull, situated between
 the orbits. This sinus enters the postorbital in chasmosaurines and *Pachyrhinosaurus*. The
 sinus enters the frontal and parietal in *Zuniceratops* and *Centrosaurus*.

(37) Postorbital, position of postorbital horncore (Lehman, 1996, character 9):
 (0) – centred anterodorsal or dorsal to orbit, narrow base with posterior margin of
 postorbital horncore extending to or only slightly behind posterior margin of orbit.
 (1) – centred posterodorsal to orbit, broad base with posterior margin of postorbital
 horncore extending well behind posterior orbit.
 The postorbital horncore is centred anterodorsal/dorsal to the orbit in
 *Chasmosaurus*, *Kosmoceratops*, *Agujaceratops*, *Pentaceratops*, *Judiceratops*,
 *Centrosaurus*, *Albertaceratops*, and outgroup taxa. The horncore is centred posterodorsal
 to orbit in other chasmosaurines and in *Pachyrhinosaurus*. *Vagaceratops* (CMN 41357
 and TMP 1987.045.0001) was scored as “?”, because its possible horncores are low-relief
 mounds.

(38) Postorbital, orientation of postorbital horncore base (Sampson *et al.*, 2010, character 38,
 modified here):
 (0) – dorsally directed.
 (1) – anterodorsolaterally directed.
 State (1) was changed from “dorsolaterally” to “anterodorsolaterally”, as laterally
 directed postorbital horncores typically have a slight anterior orientation as well. In
 *Chasmosaurus*, the postorbital horncore is oriented both dorsally (AMNH 5402, CMN
 1254, CMN 2245, CMN 2280, CMN 34829, and ROM 839) and anterodorsolaterally
 (AMNH 5401, CMN 34832, TMP 1979.011.0147, TMP 1981.019.0175, TMP
 1983.025.0001, and UALVP 40). Horncores are dorsally-oriented in centrosaurines (but
 anterodorsolaterally oriented in *Coronosaurus* – not included in analysis) and outgroup
 taxa (but not *Turanoceratops* – anterodorsolaterally oriented), as well as some
 chasmosaurines (*Pentaceratops*, *Torosaurus* *utahensis*, *Nedoceratops*, and *Triceratops*).
 Other chasmosaurines have anterodorsolaterally-oriented horncores, although *Torosaurus*
 *latus* is dimorphic.
 *Turanoceratops tardabilis* was recoded from 0 to 1 because the postorbital
 horncore is anterodorsolaterally oriented. *Bravoceratops polyphemus* was recoded from 0
 to ? because the postorbital horncore is fragmentary and its orientation cannot be
 determined.

(39) Postorbital, length of unmodified postorbital horncore (Sampson *et al.*, 2010, character 39,
 modified here):
 (0) – short, less than 15% basal skull length.
 (1) – present, elongate, greater than 35% basal skull length.
 The term “unmodified” was added to this character description, as this character
 describes the length of a horncore unmodified by subsequent breakage or potential
 resorption of bone – both of which artificially shorten the original horncore. In
 *Chasmosaurus*, postorbital horncores are both relatively short (AMNH 5402, CMN 2245,
 CMN 2280, CMN 8802, CMN 34829, ROM 839, and UALVP 40; inferred for TMP
 1981.019.0175 and UALVP 40) and long (AMNH 5401, CMN 1254, CMN 34832, and
 TMP 1979.011.0147; inferred for TMP 1983.025.0001). Postorbital horncores are
 relatively long in other chasmosaurines (except for *Utahceratops*), and relatively short in
 centrosaurines (except for *Albertaceratops, Diabloceratops* and *Xenoceratops*), and short
 in outgroup taxa except for *Zuniceratops* and *Turanoceratops*.

(40) Postorbital, curvature of postorbital horncore in lateral view (Sampson *et al.*, 2010,
 character 40):
 (0) – posteriorly recurved.
 (1) – anteriorly curved.
 (2) – straight.
 In *Chasmosaurus*, postorbital horncores are both posteriorly recurved (AMNH
 5401, CMN 1254, CMN 2280, ROM 839, and UALVP 40) and straight (CMN 2245,
 CMN 8802, CMN 34832, and TMP 1979.011.0147) in lateral view. Posteriorly recurved
 horncores are also present in *Agujaceratops*, *Judiceratops*, *Zuniceratops*, and
 *Turanoceratops*. Anteriorly curved horncores are present in other chasmosaurines except
 for *Utahceratops* (straight), and in *Albertaceratops*. Straight horncores are present in
 *Centrosaurus*.
 *Bravoceratops polyphemus* was recoded from 1 to ? because the postorbital
 horncore is fragmentary and its orientation in lateral view cannot be determined.

(41) Postorbital, curvature of postorbital horncore in anterior view (Sampson *et al.*, 2010,
 character 41):
 (0) – medially recurved.
 (1) – laterally curved.
 (2) – straight.
 The postorbital horncore is straight in anterior view in chasmosaurines except for
 *Agujaceratops* (medially-recurved), *Anchiceratops*, *Arrhinoceratops*, and *Kosmoceratops*
 (laterally curved), as well as the centrosaurines *Albertaceratops* and *Centrosaurus* (but
 laterally curved in *Coronosaurus* – not included in analysis), and in the outgroup taxa
 *Zuniceratops* and *Turanoceratops*.
 *Turanoceratops tardabilis* and *Centrosaurus apertus* were both recoded from 1 to
 2 because their postorbital horncores are straight in anterior view. *Kosmoceratops
 richardsoni* was recoded from 2 to 1 because the postorbital horncores are laterally
 curved in anterior view.

(42) Postorbital, separation from lateral temporal fenestra (Sampson *et al.*, 2010, character 42):
 (0) – narrowly excluded from fenestra by narrow strip of jugal.
 (1) – broadly excluded from fenestra by a substantial jugal-squamosal contact.
 The postorbital is broadly excluded from the lateral temporal fenestra in
 ceratopsids and *Zuniceratops*, but narrowly excluded in *Leptoceratops* and
 *Protoceratops*.
 *Bravoceratops polyphemus* was recoded from 1 to ? because the portion of the
 jugal dorsal to the lateral temporal fenestra is not preserved, and the degree to which the
 jugal separates the postorbital from the lateral temporal fenestra cannot be determined.

(43) Palpebral, shape (Sampson *et al.*, 2010, character 43):
 (0) – rod-like, articulates with prefrontal only at its base and projects across dorsal orbit,
 ligamentous attachment.
 (1) – blocky, fully fused into dorsal orbital margin, sutural articulation with prefrontal
 and frontal.
 The palpebral is blocky in ceratopsids and *Zuniceratops*, but rod-like in
 *Leptoceratops* and *Protoceratops*.
 *Turanoceratops tardabilis* was recoded from 1 to ? because the palpebral is
 incompletely preserved.

(44) Palpebral, antorbital buttress (Sampson *et al.*, 2010, character 44):
 (0) – absent.
 (1) – present.
 The palpebral occasionally forms a thick, antorbital buttress. This buttress is
 present in chasmosaurines. In centrosaurines, the buttress is either present
 (*Pachyrhinosaurus*; and *Diabloceratops* – not included in this analysis), or absent
 (*Centrosaurus* and *Albertaceratops*; *Coronosaurus* and *Styracosaurus* as well – not
 included in this analysis). In outgroup taxa, the buttress is either present (*Zuniceratops*)
 or absent (*Leptoceratops* and *Protoceratops*).
 *Centrosaurus apertus* and *Albertaceratops nesmoi* were both recoded from 1 to 0
 because the palpebral does not form an antorbital buttress. *Pachyrhinosaurus lakustai*
 was recoded from 0 to 1 because the palpebral does form an antorbital buttress.

(45) Palpebral, extent of antorbital buttress (Sampson *et al.*, 2010, character 45, modified here):
 (0) – present along only anterodorsal portion of orbit.
 (1) – present along entire or nearly entire anterior portion of orbit.
 State (1) was changed from “entire” to “entire or nearly entire”, as the ventral
 extent of the buttress differs subtly. The buttress, where present, forms the entire/nearly
 anterior portion of the orbit in ceratopsids. The buttress forms only the anterodorsal
 portion of the orbit in *Zuniceratops*.
 *Centrosaurus apertus* and *Albertaceratops nesmoi* were both recoded from 1 to ?
 because the palpebral does not form an antorbital buttress. *Pachyrhinosaurus lakustai*
 was recoded from ? to 1 because the palpebral does form an antorbital buttress, which
 forms most of the anterior margin of the orbit.

(46) Jugal, size and orientation of jugal body (Makovicky, 2001, character 22):
 (0) – projects strongly posteroventrally, does not extend below the level of the maxillary
 tooth row.
 (1) – projects nearly ventrally, elongated to extend below the level of the maxillary tooth
 row.
 The jugal projects nearly ventrally in ceratopsids and *Zuniceratops*. The jugal
 projects strongly posteroventrally in *Leptoceratops* and *Protoceratops*.
 *Bravoceratops polyphemus* was recoded from 1 to ? because the maxillary tooth
 row is not preserved and its ventral extent cannot be compared with that of the jugal.

(47) Jugal lateral temporal process (Sampson *et al.*, 2010, character 47):
 (0) – absent.
 (1) – present, contacts or nearly contacts lateral temporal process of squamosal.
 The jugal lateral temporal process on the posterior margin of the jugal projects
 posteriorly to contact or nearly contact the lateral temporal process of the squamosal, and
 forms the anteroventral margin of the lateral temporal fenestra. The jugal lateral temporal
 process is present in *Chasmosaurus*, *Vagaceratops*, and *Judiceratops*, dimorphic in
 *Pentaceratops* (compare AMNH 1624 and AMNH 6325 – Lehman, 1998: fig. 1), but
 absent in other chasmosaurines, *Leptoceratops* and *Protoceratops*. The process is present
 in the *Albertaceratops*, *Centrosaurus*, *Pachyrhinosaurus*, and *Zuniceratops*.
 *Pachyrhinosaurus lakustai* was recoded from ? to 1 because the jugal lateral
 temporal process is present. *Pentaceratops sternbergi* was recoded from 0 to 0&1)
 because the jugal lateral temporal process is present in at least one specimen (AMNH
 1624 – Lehman, 1998: fig. 1).

(48) Jugal-lacrimal contact (Makovicky & Norell, 2006, character 26):
 (0) – reduced.
 (1) – expanded.
 The jugal-lacrimal contact is expanded in ceratopsids, but reduced in outgroup
 taxa.

(49) Epijugal attachment scar, shape (Sampson *et al.*, 2010, character 49, modified here):
 (0) – large, obtuse isosceles triangle with obtuse angle oriented towards quadratojugal.
 (1) – scar roughly equilateral in shape.
 The word “shape” was added to the character description. State (0) was changed
 from “blade like triangle” to “obtuse isosceles triangle”, as the latter better describes the
 shape of the epijugal attachment scar. The epijugal attachment scar is roughly equilateral
 in shape in ceratopsids, but obtuse isosceles shaped in outgroup taxa.

(50) Epijugal length (Sampson *et al.*, 2010, character 50, quantified):
 (0) – long, proximodistal length approximately equal to anteroposterior basal width.
 (1) – hyperlong, proximodistal length >1.25 anteroposterior basal width
 (2) – short, proximodistal length <0.75 anteroposterior basal width.
 States (0), (1) and (2) originally read “long”, “hyperlong” and “short”, but were
 changed to the above; these changes were made to better quantify the relative elongation
 of the epijugal. In *Chasmosaurus*, the epijugal is either long (AMNH 5401, ROM 839,
 and ROM 843) or short (all other specimens). The epijugal is hyperlong in *Pentaceratops*
 and *Bravoceratops*, long in *Utahceratops*, *Agujaceratops*, *Kosmoceratops*,
 *Anchiceratops*, *Arrhinoceratops*, and *Eotriceratops*, but short in other chasmosaurines.
 The epijugal is long in *Leptoceratops* and *Protoceratops*.
 *Kosmoceratops richardsoni*, *Agujaceratops mariscalensis*, *Utahceratops gettyi*,
 *Anchiceratops ornatus*, and *Arrhinoceratops brachyops* were all recoded from 1 to 0, and
 *Eotriceratops xerinsularis* was recoded from 2 to 0 because its epijugals are long
 (proximodistal length approximately equal to anteroposterior basal width).

(51) Quadratojugal-squamosal contact (Sampson *et al.*, 2010, character 51):
 (0) – absent.
 (1) – present.
 The quadratojugal-squamosal contact is present in ceratopsids, but absent in
 outgroup taxa.

(52) Lateral temporal fenestra, size and position (Chinnery & Weishampel, 1998, character 7):
 (0) – relatively large, diameter 20% or greater that of basal skull length, positioned
 posterior to orbit.
 (1) – greatly reduced, diameter 10% or less that of basal skull length, positioned entirely
 below ventral limit of orbit.
 The lateral temporal fenestra is relatively small in ceratopsids and *Zuniceratops*.
 The fenestra is relatively large in *Leptoceratops* and *Protoceratops*.

(53) Frontal, contribution to orbital margin (Forster, 1990, character 51):
 (0) – present.
 (1) – absent.
 The frontal is excluded from the orbital margin in ceratopsids, *Zuniceratops* and
 *Turanoceratops*. The frontal forms part of the orbital margin in *Leptoceratops* and
 *Protoceratops*.

(54) Frontal, contribution to dorsal temporal fenestra (Sampson *et al.*, 2010, character 54):
 (0) – present.
 (1) – absent.
 The frontal is excluded from the margin of the dorsal temporal fenestra in ceratopsids. The frontal forms part of this margin in outgroup taxa.

(55) Frontoparietal fontanelle leading into supracranial cavity complex (Sampson *et al.*, 2010,
 character 55):
 (0) – absent.
 (1) – present.
 The frontoparietal fontanelle, which leads into the supracranial cavity complex, is
 present in most chasmosaurines (except for *Kosmoceratops* and *Triceratops* *prorsus*;
 *Arrhinoceratops brachyops* and *Torosaurus* *latus* are dimorphic), and centrosaurines. The
 above chasmosaurines without a frontoparietal fontanelle do possess a supracranial sinus,
 but is roofed over by bone over ontogeny. Outgroup taxa were coded as absent, as they
 do not possess a supracranial cavity, but a frontoparietal depression instead. This latter
 feature is a depression and not a sinus, as it is not partially enclosed by bone, and is not as
 deeply excavated as the supracranial sinus; however, the frontoparietal depression is
 interpreted as being the evolutionary precursor to the supracranial sinus (Farke, 2010).
 *Kosmoceratops richardsoni* was recoded from 1 to 0 because it does not possess a
 frontoparietal fontanelle.

(56) Frontoparietal fontanelle, shape (Sampson *et al.*, 2010, character 56, modified here):
 (0) – parallel-sided.
 (1) – non-parallel-sided.
 State (0) was changed from “transversely narrow, slit-like” to “parallel-sided”,
 and state (1) was changed from “key-hole shaped, circular or elongate oval” to “non-
 parallel-sided”. The frontoparietal fontanelle is non-parallel-sided in chasmosaurines, and
 parallel-sided in centrosaurines.

(57) Parietal, anterior extent on dorsum of skull relative to occipital condyle (Sampson *et al.*,
 2010, character 57):
 (0) – anterior end of parietal located well in front of occipital condyle.
 (1) – anterior end of parietal lies directly over occipital condyle.
 The anterior end of the parietal lies directly over the occipital condyle in
 ceratopsids. This end is located well in front of the condyle in outgroup taxa.

(58) Squamosal, posterior expansion (Sampson *et al.*, 2010, character 58, operationalized:
 (0) – absent or very slight.
 (1) – present, anteroposterior distance from the posterior margin (squamosal) of the
 lateral temporal fenestra to the posterior end of the squamosal equal to or greater
 than the anteroposterior width of the lateral temporal fenestra.
 State (1) originally read “present”, but was quantified here as shown above. The
 squamosal is posteriorly expanded (1) in ceratopsids and *Protoceratops*. The squamosal
 exhibits little posterior expansion (0) in *Leptoceratops*.

(59) Squamosal, shape of expanded blade (Sampson *et al.*, 2010, character 59):
 (0) – sub-rectangular in outline.
 (1) – triangular in outline, posteriorly narrowed.
 The squamosal posterior blade is triangular in chasmosaurines, but sub-
 rectangular in centrosaurines and outgroup taxa.
 *Bravoceratops polyphemus* was recoded from 2 to 1 because the squamosal is an
 elongate triangle (also, there is no 2 state described for this character).

(60) Squamosal, anteroposterior proportion of parietosquamosal frill as measured from posterior
 margin of lateral temporal fenestra to posterior end of parietal (Sampson *et al.*, 2010,
 character 60, modified and quantified):
 (0) – squamosal less than 50% of frill length.
 (1) – squamosal greater than 70% of frill length, but less than total frill length.
 (2) – squamosal length equal to total frill length.
 “Squamosal, length relative to parietal”, was changed to the above description to
 better quantify the length of the squamosal in relation to the frill. The anterior margin of
 the frill was here defined as the posterior margin of the lateral temporal fenestra and not
 the jugal notch, as outgroup taxa do not possess a distinct jugal notch. State (0) originally
 read “squamosal much shorter than parietal, large portion of posterolateral frill made up
 of parietal” to the above. State (1) originally read “squamosal slightly shorter than
 parietal, only posterolateralmost margin of frill formed by the parietal” to the above.
 State (2) originally read “squamosal and parietal equal in length” to the above. The
 squamosal is slightly shorter (>70% to <100% ) than the total frill length in
 chasmosaurines except for *Kosmoceratops*, *Anchiceratops* and *Ojoceratops* (squamosal
 equal to total frill length). The squamosal is much shorter (<50%) than the total frill
 length in centrosaurines and outgroup taxa.

(61) Squamosal forms part of posterior margin of frill (Sampson *et al.*, 2010, character 61):
 (0) – present.
 (1) – absent.
 The squamosal is excluded from the posterior margin of the frill in
 chasmosaurines (except *Kosmoceratops* and *Ojoceratops*), centrosaurines and
 *Protoceratops*. The squamosal forms part of the posterior margin of the frill in
 *Leptoceratops*.

(62) Squamosal, anteromedial lamina forming the posterolateral floor of dorsal temporal fossa
 (Sampson *et al.*, 2010, character 62):
 (0) – absent.
 (1) – present.
 The anteromedial lamina of the squamosal is situated along the parietal-
 squamosal contact, laterally adjacent to the dorsal temporal fenestra. This lamina is
 excluded from the posterolateral floor of the dorsal temporal fossa in chasmosaurines and
 *Leptoceratops*. The lamina forms part of the posterolateral floor in centrosaurines and
 *Protoceratops*.

(63) Squamosal-quadrate contact, shape (Sampson *et al.*, 2010, character 63):
 (0) – socket-like cotylus on ventrolateral squamosal for ball-like quadrate head.
 (1) – elongate groove on medial surface of squamosal to receive lamina of quadrate.
 The squamosal-quadrate contact is elongate in ceratopsids, but socket-like in
 outgroup taxa.

(64) Squamosal, thickened, rounded swelling along medial margin (Forster, 1990, character 90,
 modified here):
 (0) – absent, dorsal surface of squamosal flat to slightly convex.
 (1) – present, dorsal surface of squamosal slightly concave.
 The presence of a raised ridge along the medial margin of the squamosal results in
 the element having a concave dorsal surface. In states (0) and (1), “lateral” was changed
 to “dorsal”, as the concavity is present on the dorsal, and not lateral, surface. This
 swelling is present in chasmosaurines except for *Kosmoceratops*, *Ojoceratops*,
 *Nedoceratops*, and *Triceratops*. The swelling is absent in centrosaurines and outgroup
 taxa.

(65) Parietosquamosal contact, shape in lateral view (Forster, 1990, character 119, modified
 here):
 (0) – straight.
 (1) – curved, laterally concave.
 In state (1), “medially” was changed to “laterally”, because this character
 describes the shape of the contact in lateral view (Andrew Farke, pers. comm.); the
 change has been made here. The parietosquamosal contact is curved in lateral view in
 chasmosaurines, but straight in centrosaurines and outgroup taxa.

(66) Parietal, concave median embayment on posterior margin (Sampson *et al.*, 2010, character
 66):
 (0) – absent.
 (1) – present.
 The term “embayment” refers to the concave medial portion of the posterior
 parietal bar, as seen in dorsal view. The existence, shape and extent of such an
 embayment pertains to the posterior margin of the parietal itself and not to the projecting
 epiparietals; epiparietals are ignored here and in characters 67 and 68, as they can
 exaggerate the parietal concavity. In *Chasmosaurus*, the concave median embayment of
 the parietal is both absent (i.e. YPM 2016 and AMNH 5402) and present (all other
 specimens). The embayment is absent in *Vagaceratops* (CMN 41357) and other
 chasmosaurines (except for *Kosmoceratops*, *Agujaceratops*, *Utahceratops*, and
 *Pentaceratops*), *Leptoceratops* and *Protoceratops*; dimorphic for *Arrhinoceratops
 brachyops*. The embayment is present in centrosaurines.
 *Bravoceratops polyphemus* was recoded from 0 to ? because the posterior parietal
 bar is insufficiently preserved to determine whether an embayment existed or not.

(67) Parietal, shape of concave median embayment (Sampson *et al.*, 2010, character 67,
 renumbered and quantified here):
 (0) – shallow, restricted to centre of margin.
 (1) – shallow, entire posterior bar is a V-shaped embayment, with the two sides of the
 embayment diverging from each other at an angle greater than 100°.
 (2) – notch-like, restricted to centre of margin, with the two sides of the notch diverging
 from each other at an angle less than 100°.
 The states were originally misnumbered as “(0)”, “(2)” and “(3)”, with no
 “(1)”; they have been changed to “(0)”, “(1)” and “(2)”, respectively (Andrew Farke,
 pers. comm.), and character codings were changed accordingly. In *Chasmosaurus*, both
 V-shaped (AMNH 5656, CMN 0491, CMN 2245, CMN 2280, NHMUK R4948, ROM
 843, and TMP 1983.025.0001) and notch-like (CMN 8800 and CMN 8803) embayments
 are present. Other chasmosaurines that possess an embayment are either V-shaped
 (*Kosmoceratops*) or notch-like (*Agujaceratops*, *Utahceratops* and *Pentaceratops*). The
 embayment is shallow in *Arrhinoceratops brachyops* and centrosaurines.

(68) Parietal, location of posteriormost point of posterior bar (Sampson *et al.*, 2010, character
 68):
 (0) – on midline.
 (1) – between midline and lateralmost corner.
 (2) – at lateralmost corner adjacent to squamosal.
 In *Chasmosaurus*, the posteriormost point of the posterior bar of the parietal is
 either between the midline and lateralmost corner (AMNH 5656, CMN 2245, CMN
 2280, CMN 8800, CMN 8803, NHMUK R4948, and TMP 1983.025.0001) or the
 lateralmost corner adjacent to the squamosal (ROM 843). In other chasmosaurines, the
 posteriormost point is on the midline (*Torosaurus* *utahensis*), between the midline and
 lateralmost corner (*Utahceratops*, *Anchiceratops* and *Arrhinoceratops*), and at the
 lateralmost corner (*Kosmoceratops* and *Pentaceratops*); *Torosaurus latus* is dimorphic
 (midline, between midline and lateralmost corner). The posteriormost point is between
 the midline and lateralmost corner in centrosaurines, and on the midline in outgroup taxa.
 *Bravoceratops polyphemus* was recoded from 0 to ? because the posterior parietal
 bar is insufficiently preserved.

(69) Parietosquamosal frill, midline length relative to basal skull length (Sampson *et al.*, 2010,
 character 69, modified here):
 (0) – short, 0.70 or less.
 (1) – elongate, 0.85 or more.
 The anterior extent of the frill – anterior edge of parietal at midline – was better
 defined here by changing “length” to “midline length”; the posterior extent of the frill is
 the posteriormost extent of the parietal, and not of the epiparietals. The parietosquamosal
 frill is relatively elongate in chasmosaurines (except for *Nedoceratops* and *Triceratops*)
 and centrosaurines, but relatively short in outgroup taxa.

(70) Parietosquamosal frill, location of maximum transverse width (Sampson *et al.*, 2010,
 character 70):
 (0) – posteriorly, at rear margin of frill.
 (1) – anteriorly, in association with proximal half of frill.
 (2) – frill relatively equal in width.
 In *Chasmosaurus*, the parietosquamosal frill is widest posteriorly (CMN 2280,
 ROM 843, and TMP 1983.025.0001) or approximately equal in width (AMNH 5401,
 AMNH 5402, AMNH 5656, CMN 2245, CMN 8800, NHMUK R4948, ROM 839, TMP
 1993.082.0001, and YPM 2016). In *Vagaceratops* (CMN 41357 and TMP
 1987.045.0001), the frill is approximately equal in width between both margins. In other
 chasmosaurines, the frill is widest anteriorly (*Kosmoceratops*, *Agujaceratops*,
 *Utahceratops*, *Pentaceratops*, *Anchiceratops*, and *Arrhinoceratops*) or approximately
 equal in width (*Torosaurus*, *Nedoceratops*, and *Triceratops*). The frill is widest
 posteriorly in *Centrosaurus* and *Pachyrhinosaurus*. The frill is widest posteriorly in
 *Protoceratops* and *Leptoceratops*.
 *Albertaceratops nesmoi* was recoded from 1 to ? because the frill is transversely
 compressed due to taphonomic compaction, and the widest part of the frill cannot be
 determined with certainty.

(71) Parietal, parietal sulci (Sampson *et al.*, 2010, character 71):
 (0) – absent.
 (1) – present.
 Refers to the sulci than connect the dorsal temporal fenestra and supracranial
 sinus complex. These sulci are present in ceratopsids, but absent in outgroup taxa.

(72) Parietal, overall lateral shape (Sampson *et al.*, 2010, character 72):
 (0) – nearly straight along midline in lateral view and gently arched from side to side.
 (1) – ``saddle-shaped``, dorsally concave in lateral view with upturned posterior margin,
 and arched strongly from side to side.
 The parietal is saddle-shaped in *Triceratops*; in other neoceratopsians, the parietal
 is nearly straight along the midline.

(73) Parietal fenestrae (Forster, 1990, character 84):
 (0) – absent.
 (1) – present.
 Parietal fenestrae are present in chasmosaurines (except for *Triceratops*),
 centrosaurines, *Protoceratops*, and *Zuniceratops*. Parietal fenestrae are absent in
 *Leptoceratops*.

(74) Parietal, rim on medial margin of dorsal temporal fenestra (Forster, 1990, character 86):
 (0) – absent.
 (1) – present, well-defined, laterally projecting rim defines medial margin of fenestra.
 “Fenstra” (spelling error) was changed to “fenestra”. This rim is a ridge
 that separates the raised anteromedial platform of the parietal from the relatively low
 dorsal temporal fenestra. This rim is present in chasmosaurines (except for *Torosaurus*
 and *Triceratops*) and centrosaurines, but absent in outgroup taxa.

(75) Parietal, sharp median crest (Sampson *et al.*, 2010, character 75):
 (0) – present.
 (1) –absent.
 This crest is a narrow ridge that occurs along the midline of the parietal. This crest
 is absent in ceratopsids, but present in outgroup taxa.

(76) Parietal, anteroposterior width of posterior bar at narrowest point (Holmes *et al.*, 2001,
 character 22, modified here):
 (0) – narrow and strap-like, less than 10% total parietal length.
 (1) – broad, 20% or more of total parietal length.
 Anteroposterior “thickness” was replaced with “width”, as the term thickness
 implies a dorsoventral measurement. The posterior parietal bar is narrow and strap-like in
 chasmosaurines with parietal fenestrae, except for *Anchiceratops*, *Arrhinoceratops*,
 *Ojoceratops*, *Torosaurus*, and *Nedoceratops*. The bar is also strap-like in *Protoceratops*.
 The bar is broad in centrosaurines and *Zuniceratops*.
 *Bravoceratops polyphemus* was recoded from 1 to ? because most of the posterior
 parietal bar is missing and the narrowest point of this bar therefore cannot be determined.

(77) Parietal, relative anteroposterior width of broad posterior bar (Sampson *et al.*, 2010,
 character 77, modified here):
 (0) – subequal medial to lateral.
 (1) – tapering so that the narrowest point occurs medially.
 Anteroposterior “depth” was replaced with “width”, as the term depth implies a
 dorsoventral measurement. In neoceratopsians which possess parietal fenestrae and
 therefore a posterior parietal bar, this bar is subequal medial to lateral except for
 *Utahceratops* and *Pentaceratops*.

(78) Parietal, cross-sectional shape of median bar (Sampson *et al.*, 2010, character 78; modified
 by Campbell (2015)):
 (0) – triangular- to diamond-shaped, with apex oriented dorsally, and ventral surface flat
 to convex.
 (1) – rectangular or subrectangular, margin facing parietal fenestrae thick and oriented
 sub-perpendicular to parietal surface.
 (2) – round to lenticular.
 (3) – v-shaped, opening ventrally.
 (4) – meniscus-shaped, with concave surface facing ventrally.
 In *Chasmosaurus*, the medial parietal bar is triangular- to diamond-shaped
 (AMNH 5401, AMNH 5402, ROM 839, ROM 843), rectangular to subrectangular
 (AMNH 5656, CMN 0491, CMN 2245, CMN 2280, CMN 8800, NHUK R4948, TMP
 1981.019.0175, and YPM 2016), or round to lenticular (TMP 1983.025.0001). In
 *Vagaceratops*, the bar is triangular- to diamond-shaped (CMN 41357). In other
 chasmosaurines, the bar is triangular- to diamond-shaped (*Kosmoceratops*,
 *Anchiceratops*, *Arrhinoceratops*, and *Ojoceratops*), round to lenticular (*Agujaceratops*,
 *Utahceratops*, *Pentaceratops*, and *Coahuilaceratops*), and v-shaped (*Torosaurus*,
 *Nedoceratops*, and *Bravoceratops*). In centrosaurines, the bar is either triangular- to
 diamond-shaped (*Albertaceratops*) or v-shaped (*Centrosaurus* and *Pachyrhinosaurus*).
 The bar is triangular- to diamond shaped in the outgroup taxa *Protoceratops* and
 *Zuniceratops*.

(79) Parietal, median bar, transverse width (Holmes *et al.*, 2001, character 23):
 (0) – narrow and strap-like, transverse width less than 10% total parietal length.
 (1) – relatively wide, transverse width, transverse width 15% or more of total parietal
 length.
 The medial parietal bar is strap-like in chasmosaurines except for *Kosmoceratops*,
 *Anchiceratops*, *Arrhinoceratops*, *Torosaurus*, and *Nedoceratops*; *Anchiceratops* is
 dimorphic. The bar is strap-like in *Leptoceratops* and *Protoceratops*. The bar is relatively
 wide in centrosaurines.

(80) Parietal fenestrae, orientation (Sampson *et al.*, 2010, character 80):
 (0) – long axis directed transversely.
 (1) – long axis directed axially.
 (2) – axial and transverse axes equal.
 In *Chasmosaurus*, the parietal fenestrae are axially elongate (AMNH 5401,
 AMNH 5656, CMN 2245, CMN 2280, CMN 8800, NHMUK R4948, ROM 839, TMP
 1983.025.0001, and TMP 1993.082.0001) and equal both axially and transversely
 (AMNH 5402, ROM 843, and YPM 2016). In *Vagaceratops* and *Kosmoceratops*, the
 fenestrae are transversely elongate. The fenestrae are axially elongate in other
 chasmosaurines (except for *Torosaurus* – equal axially and transversely) and
 centrosaurines, but transversely elongate in outgroup taxa.

(81) Parietal fenestrae, maximum proximodistal length (Sampson *et al.*, 2010, character 81):
 (0) – 35% or less total parietal length.
 (1) – 45% or more total parietal length.
 The parietal fenestrae are relatively long in chasmosaurines (except for
 *Kosmoceratops*, *Anchiceratops*, *Arrhinoceratops*, and *Torosaurus*) and centrosaurines,
 but relatively short in *Protoceratops*.

(82) Parietosquamosal frill, marginal undulations (Sampson *et al.*, 2010, character 82):
 (0) – absent.
 (1) – present.
 These undulations refer to the scalloped free margin of the parietosquamosal frill,
 and correspond to epiossification (episquamosal and epiparietal) attachment sites. These
 undulations are present in ceratopsids, but absent in *Leptoceratops* and *Protoceratops*.
 *Turanoceratops tardabilis* was recoded from 1 to ? because none of the frill is
 preserved.

(83) Parietosquamosal frill, imbrications of undulations (Dodson *et al.*, 2004, character 34):
 (0) – absent.
 (1) – present.
 Imbrication of parietosquamosal frill marginal undulations is absent in
 chasmosaurines (but is dimorphic in *Kosmoceratops*), *Leptoceratops* and *Protoceratops*.
 Imbricated undulations are present in centrosaurines.

Epiossifications on parietosquamosal frill

(84) Marginal dermal ossifications on parietosquamosal frill (Sampson *et al.*, 2010, character
 84):
 (0) – absent.
 (1) – present.
 Character 84 does not appear to be independent of 82 – in taxa possessing
 marginal undulations on the frill, these undulations are adorned by epiossifications.

(85) Episquamosals on midlateral squamosal margin, basal width (Sampson *et al.*, 2010,
 character 85, modified here):
 (0) – small, less than 50 mm wide in adults.
 (1) – large and wide, greater than 90 mm wide in adults.
 This character refers to the basal width of episquamosals, but was originally
 written as “length” – the term “width” is used here instead. The midlateral squamosals
 are here defined as the episquamosals occupying the medial third of the lateral margin of
 the squamosal. In *Chasmosaurus*, episquamosals on the midlateral margin of the
 squamosal are both relatively small (AMNH 5402, CMN 2245, TMP 1981.019.0175,
 TMP 1983.025.0001, and UALVP 40) and large (AMNH 5401, AMNH 5656, CMN
 2280, CMN 8800, CMN 8802, CMN 34829, NHMUK R4948, ROM 839, ROM 843, and
 YPM 2016). Such episquamosals are large in other chasmosaurines, but small in
 centrosaurines.
 *Agujaceratops mariscalensis* and *Kosmoceratops richardsoni* were both recoded
 from 0 to 1 because the episquamosals on the midlateral margin of the squamosal are
 large and wide (greater than 90 mm).

(86) Episquamosal, location of largest/longest episquamosal (Sampson *et al.*, 2010, character 86,
 modified here):
 (0) – anteriormost episquamosal by far the largest.
 (1) – posteriormost episquamosal by far the largest.
 This character was modified from Sampson *et al.* (2010), which originally had
 three states. The original (0) state, “episquamosals subequal in size”, was removed
 because it does not pertain to the location of the largest episquamosal; the original (1) and
 (2) were recoded as (0) and (1), respectively. Taxa originally coded as “episquamosals
 subequal in size” are coded here as “?”. Episquamosals are subequal in size in
 chasmosaurines, except for *Kosmoceratops*, *Utahceratops*, *Pentaceratops*, and
 *Anchiceratops* (posteriormost episquamosal the largest). *Bravoceratops* was coded as
 “?”, because the posterior end of the squamosal and episquamosal(s) is not preserved.
 Episquamosals are subequal in size in the centrosaurines *Centrosaurus*,
 *Pachyrhinosaurus*, and *Albertaceratops*.
 *Albertaceratops nesmoi*, *Centrosaurus apertus*, *Arrhinoceratops brachyops*,
 *Ojoceratops fowleri*, *Torosaurus utahensis*, *Nedoceratops hatcheri*, *Triceratops horridus*,
 and *Triceratops prorsus* were all recoded from 0 to ? because the episquamosals are
 subequal in size. *Kosmoceratops richardsoni*, *Utahceratops gettyi*, *Pentaceratops
 sternbergi*, and *Anchiceratops ornatus* were all recoded from 2 to 1 because the largest
 episquamosal occurs on the posterior end of the squamosal. *Bravoceratops polyphemus*
 was recoded from 1 to ? because the posterior end of the squamosal is not preserved and
 the size of the posteriormost episquamosals cannot be determined.

(87) Episquamosal, midlateral, shape (Sampson *et al.*, 2010, character 87):
 (0) – crescentic or ellipsoidal.
 (1) – triangular or elongate.
 Episquamosals on the midlateral margin of the squamosal are triangular in
 chasmosaurines, but crescentic or ellipsoidal in centrosaurines.

(88) Episquamosal locus ES1 shape (Sampson *et al.*, 2010, character 88):
 (0) – small and crescentic.
 (1) – low, raised D-shaped process.
 (2) – large triangular process.
 (3) – elongate hook.
 Here, episquamosals are numbered sequentially posterior to anterior on the
 squamosal. The posteriormost episquamosal is a low raised D-shaped process in
 chasmosaurines, except for *Kosmoceratops* (elongate hook), and *Utahceratops*,
 *Pentaceratops* and *Anchiceratops* (well-developed larger triangular process). The
 posteriormost episquamosal is small and crescentic-shaped in *Centrosaurus*.

(89) Episquamosal ES2 shape (Sampson *et al.*, 2010, character 89, modified here):
 (0) – small and crescentic.
 (1) – low, raised D-shaped process.
 (2) – large triangular process.
 For this character, “episquamosal locus S2 shape” was changed to “episquamosal
 ES2 shape”, as this character describes the shape of episquamosal 2, and not of the
 underlying locus. Episquamosal 2 is a low raised D-shaped process in chasmosaurines,
 except for *Kosmoceratops*, *Utahceratops*, *Pentaceratops*, and *Anchiceratops* (well-
 developed large triangular process). Episquamosal 2 is small and crescentic in
 *Centrosaurus*.
 *Bravoceratops polyphemus* was recoded from 1 to ? because episquamosal 2 is
 not preserved and its shape cannot be determined.

(90) Episquamosal ES2 size relative to other episquamosals (Sampson *et al.*, 2010, character 90,
 modified here):
 (0) – subequal.
 (1) – second only to ES1 in size, larger than ES3.
 Episquamosal locus S2” was changed to “episquamosal ES2”, because a locus is
 not the same as an epiossification and should not be compared. Episquamosal 2 is
 subequal relative to other episquamosals in chasmosaurines, except for *Kosmoceratops*,
 *Utahceratops*, *Pentaceratops*, and *Torosaurus* *utahensis*. Episquamosal 2 is smaller than
 1 and larger than 3 in *Centrosaurus*.
 *Bravoceratops polyphemus* was recoded from 0 to ? because episquamosal 2 is
 not preserved and its size relative to other episquamosals cannot be determined.

(91) Epiparietosquamosal, marginal ossification crossing squamosal-parietal contact (Dodson *et
 al.*, 2004, character 43):
 (0) – absent.
 (1) – present.
 A marginal ossification crossing the squamosal-parietal contact is absent in
 *Chasmosaurus*. In *Vagaceratops*, such an ossification is also both present (TMP
 1987.045.0001) and absent (CMN 41357). Such an ossification is also present in
 *Arrhinoceratops*, *Kosmoceratops*, *Torosaurus* *utahensis*, *Eotriceratops*, *Nedoceratops*,
 and *Triceratops*, and dimorphic in *Anchiceratops*, and absent in other chasmosaurines. Such an ossification is also present in *Centrosaurus*, *Albertaceratops* and
 *Pachyrhinosaurus*.
 *Albertaceratops nesmoi*, *Centrosaurus apertus*, and *Pachyrhinosaurus lakustai*
 were all recoded from 0 to 1 because they all possess a marginal epiossification crossing
 the squamosal-parietal contact (epiparietosquamosal).

(92) Epiparietosquamosal, shape (Sampson *et al.*, 2010, character 92):
 (0) – small and crescentic.
 (1) –strongly recurved process.
 (2) – well-developed triangular process.
 Chasmosaurines have a well-developed triangular process, except for
 *Kosmoceratops* (strongly recurved process). In centrosaurines, this ossification is small
 and crescentic.

(93) Epiparietals, number per side (Holmes *et al.*, 2001, character 28, modified here):
 (0) – three.
 (1) – four or more.
 State (1) was changed from “five” to “four”. In *Vagaceratops* (TMP
 1987.045.0001 and TMP 1998.102.0008), the fifth epiossification from the midline of the
 parietal is variably co-ossified with the squamosal, making this epiossification an
 epiparietosquamosal. In these cases, only four epiparietals are entirely articulated with
 each side of the parietal, necessitating the above change to (1). In *Chasmosaurus*,
 epiparietal count is either five (YPM 2016) or three (all other specimens). Three
 epiparietals are present in other chasmosaurines, except for *Vagaceratops* (four to five)
 and *Torosaurus* (five to six); *Anchiceratops* is dimorphic (three to four). Centrosaurines
 have at least five epiparietals.
 *Anchiceratops ornatus* was recoded from 0 to 0&1 because it possesses either
 three (e.g. UALVP 1618 and AMNH 5273) or four epiparietals (e.g. CMN 8535 and
 UW 2419) on each side of the posterior parietal bar (Mallon *et al.*, 2011).

(94) Epiparietals, fused to adjacent epiparietal at base (Holmes *et al.*, 2001, character 29):
 (0) – absent.
 (1) – present.
 Basal fusion between adjacent epiparietals is absent in neoceratopsians, except for
 *Vagaceratops* and *Kosmoceratops* (and in *Coronosaurus* – not included in analysis).

(95) Epiparietal P0 (Sampson *et al.*, 2010, character 95):
 (0) – absent.
 (1) – present.
 Epiparietals are numbered in sequential order from the midline of the parietal and
 progressing anteriorly. An epiparietal on the midline of the parietal is present in
 *Ojoceratops*, *Torosaurus utahensis*, and *Triceratops*, but absent in other ceratopsids
 (*Avaceratops* – not included in analysis – has a locus for P0, but the epiparietal is not
 present).

(96) Epiparietal at P1 locus (Sampson *et al.*, 2010, character 96,
 modified here):
 (0) – absent.
 (1) – present.
 This character was changed from “epiparietal locus DPP1” to “epiparietal at P1
 locus”, because the first parietal process on the posterior surface of the parietal (dorsal
 parietal process = DPP) adjacent to the midline in chasmosaurines is interpreted here as
 epiparietal 1. All ceratopsids possess a P1 locus that is adorned with an epiparietal; the
 two exceptions in this analysis are *P. lakustai* and *Albertaceratops*, which do not have a
 P1 epiparietal. The *Chasmosaurus* skull CMN 2245 does not have a P1 epiparietal as
 preserved, but is inferred as would having one later in life, based on its well-developed
 P1 locus that is similar in shape and size to that of its P3 locus which is adorned by an
 epiparietal.
 *Pachyrhinosaurus lakustai* was recoded from 1 to 0 because it does not possess an
 epiparietal at its P1 locus. *Kosmoceratops richardsoni*, *Anchiceratops ornatus*,
 *Arrhinoceratops brachyops*, *Ojoceratops fowleri*, *Torosaurus latus*, *Torosaurus
 utahensis*, *Triceratops horridus*, and *Triceratops prorsus* were all recoded from 0 to 1,
 and *Agujaceratops mariscalensis* and *Nedoceratops hatcheri* were both recoded from ? to
 1 because they all possess an epiparietal at their P1 locus. *Bravoceratops polyphemus* was
 recoded from 0 to ? because the posterior parietal bar is insufficiently complete to
 determine whether an epiparietal 1 locus was present.

(97) Epiparietal, P1 shape (Sampson *et al.*, 2010, character 97, modified here):
 (0) – low D-shaped process.
 (1) – elongate, flattened process or spike.
 (2) – strongly recurved triangular or recurved low rugose triangular process.
 (3) – well-developed triangular process.
 (4) – elongate low process.
 “Locus P1 shape” was changed to “P1 shape”, as the states refer to the shape of
 the adorning epiparietal. In state (2), “gnarled” was changed to “rugose”, as the latter
 better describes the irregular shape of such epiparietals. In *Chasmosaurus*, epiparietal 1 is
 either low D-shaped (AMNH 5402, CMN 0491, CMN 8800, CMN 8803, ROM 843, and
 YPM 2016) or a well-developed triangular process (AMNH 5656, CMN 2280, and TMP
 1983.025.0001). In *Vagaceratops* and *Kosmoceratops*, epiparietal 1 is a strongly recurved
 process. In other chasmosaurines, epiparietal 1 is an elongate low process, except for
 *Utahceratops*, *Pentaceratops* and *Anchiceratops* (well-developed triangular process). In
 *Centrosaurus*, epiparietal 1 is an elongate spike.
 *Albertaceratops nesmoi* and *Pachyrhinosaurus lakustai* were both recoded from 1
 to ? because neither of them have epiparietal 1 (the medialmost epiparietal is interpreted
 here as 3 in *A*. *nesmoi* and 2 in *P*. *lakustai*).

(98) Epiparietal, P1 orientation (Sampson *et al.*, 2010, character 98, modified by Wick &
 Lehman (2013) and here):
 (0) – epiparietal oriented in the plane of the frill.
 (1) – directed anterodorsally.
 (2) – P1 occurs on dorsal surface of parietal.
 “Locus P1 orientation” was changed to “P1 orientation”, as the states refer to the
 orientation of the adorning epiparietal. In state (0), “posteriorly” was removed, as not all
 P1s that are oriented in the plane of the frill are directed posteriorly (some are oriented
 posteromedially). In *Chasmosaurus*, epiparietal 1 is oriented either posteriorly (AMNH
 5402, AMNH 5656, CMN 8803, and TMP 1983.025.0001) or anterodorsally (all other
 specimens). In other chasmosaurines, epiparietal 1 is oriented in the plane of the frill,
 except for *Vagaceratops*, *Kosmoceratops*, *Utahceratops*, and *Pentaceratops*, which are
 anterodorsally directed; *Anchiceratops* and *Bravoceratops* have a P1 on the dorsal
 surface of the parietal. Epiparietal 1 is anterodorsally-oriented in *Centrosaurus*.
 *Albertaceratops nesmoi* and *Pachyrhinosaurus lakustai* were both recoded from 0
 to ? because neither have epiparietal 1.

(99) Epiparietal, P1 curvature (Sampson *et al.*, 2010, character 99, modified here):
 (0) – straight
 (1) – laterally curved.
 (2) – medially curved.
 (3) – anterodorsally curved.
 “Locus P1 curvature” was changed to “P1 curvature”, as the states refer to the
 curvature of the body of the epiparietal; state (3) was also changed from “dorsally”
 curved to “anterodorsally curved”, because there are no dorsally curved P1s in this
 analysis. In *Chasmosaurus*, epiparietal 1 is straight. Epiparietal 1 is anterodorsally curved
 in *Vagaceratops*, *Kosmoceratops*, *Utahceratops*, *Pentaceratops*, and *Anchiceratops*, but
 straight in other chasmosaurines. Epiparietal 1 is anterodorsally curved in *Centrosaurus*.
 *Centrosaurus apertus* was recoded from 2 to 3 because epiparietal 1 is
 anterodorsally curved. *Albertaceratops nesmoi* was recoded from 1 to ?, and
 *Pachyrhinosaurus lakustai* from 2 to ? because neither possess epiparietal 1.
 *Bravoceratops polyphemus* was recoded from 3 to ? because the existence of epiparietal 1
 is unknown.

(100) Epiparietal, P2 shape (Sampson *et al.*, 2010, character 100, modified here):
 (0) – low D-shaped process.
 (1) – elongate, flattened process or spike.
 (2) – strongly recurved triangular or recurved, low rugose triangular process.
 (3) – well-developed triangular process.
 (4) – elongate low process.
 “Locus P2 shape” was changed to “P2 shape”, as the states refer to the shape of
 the adorning epiparietal. In state (2), “gnarled” was changed to “rugose”, as the latter
 better describes the irregular shape of such epiparietals. In *Chasmosaurus*, epiparietal 2 is
 either low D-shaped (CMN 8800 and YPM 2016) or a well-developed triangular process
 (all other specimens). In other chasmosaurines, epiparietal 2 is variably a strongly
 recurved triangular (*Vagaceratops* and *Kosmoceratops*), a well-developed (*Utahceratops*,
 *Pentaceratops*, and *Anchiceratops*) or an elongate low process (*Arrhinoceratops*,
 *Torosaurus*, and *Triceratops*). Epiparietal 2 is a strongly recurved triangular process in
 *Centrosaurus* and *Pachyrhinosaurus*.
 *Albertaceratops nesmoi* was recoded from 3 to ? because it does not possess
 epiparietal 2. *Centrosaurus apertus* and *Pachyrhinosaurus lakustai* were both recoded
 from 1 to 2 because epiparietal 2 is a recurved triangular process. *Ojoceratops fowleri*
 was recoded from 4 to ? because epiparietal 2 is not preserved (but the locus for it is).

(101) Epiparietal P2 curvature (Sampson *et al.*, 2010, character 101, modified here):
 (0) – straight.
 (1) – medially or laterally curved in the plane of the frill.
 (2) – recurved onto dorsal surface of frill.
 In this character, “locus P2 curvature” was changed to “P2 curvature”, as the
 states refer to the curvature of the adorning epiparietal. Epiparietal 2 is straight in
 *Chasmosaurus* and other chasmosaurines, except for *Vagaceratops* and *Kosmoceratops*
 (dorsally recurved). Epiparietal 2 is medially curved in *Centrosaurus* and
 *Pachyrhinosaurus*.
 *Centrosaurus apertus* was recoded from 0 to 1 because epiparietal 2 is medially
 curved in the plane of the frill. *Albertaceratops nesmoi* was recoded from 0 to ? because
 it does not possess epiparietal 2. *Ojoceratops fowleri* was recoded from 0 to ? because
 epiparietal 2 is not preserved (but the locus for it is).

(102) Epiparietal P3 shape (Sampson *et al.*, 2010, character 102, modified here):
 (0) – low raised D-shaped process.
 (1) – elongate spike.
 (2) – strongly recurved triangular or recurved, low rugose triangular process.
 (3) – well-developed triangular process.
 (4) – elongate, low process.
 “Locus P3 shape” was changed to “P3 shape”, as the states refer to the shape of
 the adorning epiparietal. In state (2), “gnarled” was changed to “rugose”, as the latter
 better describes the irregular shape of such epiparietals. In *Chasmosaurus*, epiparietal 3 is
 either a low, raised D-shaped (YPM 2016) or a well-developed triangular (all other
 specimens) process. In other chasmosaurines, epiparietal 3 is a strongly recurved
 triangular (*Vagaceratops* and *Kosmoceratops*), well-developed triangular (*Utahceratops*,
 *Pentaceratops* and *Anchiceratops*) or an elongate, low process (*Arrhinoceratops*
 *Ojoceratops*, *Torosaurus*, and *Triceratops*). Epiparietal 3 is an elongate (modified) spike
 in *Albertaceratops*, elongate spike in *Pachyrhinosaurus* and a low raised D-shaped
 process in *Centrosaurus*.
 *Albertaceratops nesmoi* was recoded from 3 to 1, and *Pachyrhinosaurus lakustai*
 from 0 to 1 because epiparietal 3 is an elongate spike. *Centrosaurus apertus* was recoded
 from 3 to 0 because epiparietal 4 is a low D-shaped process.

(103) Epiparietal, P3 orientation (Sampson *et al.*, 2010, character 103, modified here):
 (0) – epiparietal oriented in the plane of the frill.
 (1) – directed anterodorsally.
 “Locus P3 orientation” was changed to “P3 orientation”, as the character refers to
 the orientation of the adorning epiparietal. In state (0), “posteriorly” was removed, as not
 all P3s that are oriented in the plane of the frill are oriented posteriorly (some are oriented
 posterolaterally). In *Chasmosaurus*, epiparietal 3 is either anterodorsally oriented (YPM
 2016) or oriented in the plane of the frill (all other specimens). In other chasmosaurines,
 epiparietal 3 is oriented in the plane of the frill, except for *Vagaceratops* and
 *Kosmoceratops*. Epiparietal 3 is oriented in the plane of the frill in *Centrosaurus*,
 *Albertaceratops* and *Pachyrhinosaurus*; the distal end of epiparietal 3 is twisted dorsally
 in some specimens of *Pachyrhinosaurus*, although this epiparietal is largely oriented in
 the plane of the frill.

Braincase

(104) Basioccipital, contribution to occipital condyle (Forster, 1990, character 71):
 (0) – forms approximately 2/3 of occipital condyle.
 (1) – forms 1/3 of the occipital condyle.
 The basioccipital forms 1/3 of the occipital condyle in ceratopsids, and 2/3 of the
 occipital condyle in *Protoceratops* and *Leptoceratops*.
 *Leptoceratops gracilis* was recoded from ? to 0 because the basioccipital forms
 2/3 of the occipital condyle.

(105) Supraoccipital, contribution to foramen magnum (Forster, 1990, character 63):
 (0) – forms dorsal margin of foramen magnum.
 (1) – eliminated from margin by exoccipital-exoccipital contact on midline.
 The supraoccipital is eliminated from the margin of the foramen magnum in
 neoceratopsians, except for *Pachyrhinosaurus*, *Leptoceratops*, and *Protoceratops*.

(106) Supraoccipital, ventrolateral processes (Sereno, 1999, character 131):
 (0) – absent.
 (1) – present.
 These processes extend ventrolaterally and overlap the exoccipitals. These
 processes are present in neoceratopsians, except for *Leptoceratops* and *Protoceratops*.

(107) Exoccipital, exits for cranial nerves in exoccipital (Forster, 1990, character 68):
 (0) – three foramina.
 (1) – two foramina.
 There are two foramina for cranial nerve exits in the exoccipital in
 neoceratopsians, except for *Protoceratops*.

(108) Paroccipital process, dorsoventral distal expansion (Forster, 1990, character 66):
 (0) – distal process only slightly expanded.
 (1) – distal process expanded to at least 0.8 two times the depth at its narrowest point.
 State (0) is not quantified here, but it should be; this quantification was not done
 here, as it does not affect the outcome of the analysis. The paroccipital process, a lateral
 projection of the exoccipital, is greatly expanded distally in neoceratopsians, except for
 *Leptoceratops* and *Protoceratops*.

Palate

(109) Ectopterygoid, contributes to palate and contacts the jugal (Forster, 1990, character 32):
 (0) – present.
 (1) – absent.
 The ectopterygoid contributes to the palate and contacts the jugal in
 *Leptoceratops* and *Protoceratops*, but does not in other neoceratopsians.

(110) Secondary palate, relative contribution of maxilla (Sampson *et al.*, 2010, character 110):
 (0) – maxilla forms at least 45% of the secondary palate.
 (1) – maxilla contributes only to the posterior portion, forms 30% or less of secondary
 palate.
 The maxilla forms a relatively small portion of the secondary palate in
 neoceratopsians, except for *Leptoceratops* and *Protoceratops*.

(111) Palatine, shape and relationship to maxilla (Sampson *et al.*, 2010, character 111):
 (0) – palatine contacts nearly the entire medial surface of the maxilla, restricting size of
 choanae, anterodorsal process embraces posterior end of vomer.
 (1) – palatine contacts only the posterior one-third of medial surface of maxilla, contact
 with vomer lost, choanae enlarged.
 The palatine contacts only the posterior one-third of the medial surface of the
 maxilla in neoceratopsians, except for *Leptoceratops* and *Protoceratops*.

Lower jaw

(112) Lower jaw, level of mandibular articulation (Forster, 1990, character 74):
 (0) – at or slightly below occlusal surface of tooth row.
 (1) – depressed well below level of occlusal surface of tooth row.
 The mandibular articulation of the lower jaw is depressed well below the tooth
 row occlusal surface in neoceratopsians, except for *Leptoceratops* and *Protoceratops*.

(113) Predentary, length relative to dentary (Sampson *et al.*, 2010, character 113):
 (0) – equal to or more than two-thirds of dentary length.
 (1) – less than two-thirds of dentary length.
 The predentary is less than two-thirds the length of the dentary in
 neoceratopsians, except for *Leptoceratops* and *Protoceratops*.

(114) Predentary, dentary processes (Sampson *et al.*, 2010, character 114):
 (0) – ventral processes much longer than abbreviated dorsal processes.
 (1) – dorsal and ventral processes elongate and subequal in length.
 Character 114 does not appear to be independent of 113. The predentary of
 protoceratopsids is 2/3 the length of the dentary because the ventral process of the
 predentary is long; therefore, 113(0) always pairs with 114(0), and 113(1) always pairs
 with 114(1) (Andrew Farke, pers. comm.).

(115) Predentary, orientation of triturating surface (Dodson, 2004, character 57):
 (0) – nearly horizontal.
 (1) – inclined steeply laterally.
 The triturating surface of the predentary is inclined steeply laterally in
 centrosaurines, but is nearly horizontal in chasmosaurines and outgroup taxa.

(116) Dentary lateral ridge confluent with cutting surface of predentary (Sampson *et al.*, 2010,
 character 116):
 (0) – present.
 (1) – absent.
 Character 116 does not appear to be independent of 115. In centrosaurines, the
 lateral edge of the predentary cutting surface lies ventral to its medial edge, with the
 lateral edge confluent with the lateral ridge of the dentary, and resulting in a
 dorsolaterally-facing cutting surface. In chasmosaurines, the lateral and medial edges of
 the cutting surface are both horizontal, forming a dorsally-facing cutting surface that is
 above the lateral ridge of the dentary. For these reasons, 115(1) pairs with 116(0), and
 115(0) pairs with 116(1), respectively, in ceratopsids (Andrew Farke, pers. comm.). The
 only exceptions to this pairing pattern in this analysis are the outgroup taxa
 *Leptoceratops* and *Protoceratops*, which both code as 115(0) and 116(0).
 *Protoceratops andrewsi* was recoded from ? to 0 because the lateral edge of the
 dentary is confluent with the cutting surface of the predentary.

(117) Dentary, shape of ventral margin in adults (Forster, 1990, character 73):
 (0) – strongly convexly bowed.
 (1) – straight.
 The ventral margin of the dentary is straight in neoceratopsians, except for
 *Leptoceratops* and *Protoceratops*; most of the other leptoceratopsids, and psittacosaurs
 are also variably bowed – not included in analysis.

(118) Dentary, posterior extent of tooth row (Chinnery & Weishampel, 1998, character 18):
 (0) – terminates at the centre of the coronoid process.
 (1) – terminates posterior to the coronoid process.
 The posterior extent of the tooth row on the dentary terminates posterior to the
 coronoid process in neoceratopsians, except for *Leptoceratops*, *Protoceratops*, and
 *Zuniceratops*.

(119) Dentary, shape of coronoid process (Sampson *et al.*, 2010, character 119):
 (0) – short, with gently convex apex, base of ascending ramus anteroposteriorly
 expanded.
 (1) – tall, expanded at apex into anteriorly projecting hook, base of ascending ramus
 anteroposteriorly restricted.
 The coronoid process of the dentary is tall in neoceratopsians, except for
 *Leptoceratops* and *Protoceratops*.

(120) Dentary, separation of body from ascending ramus of coronoid process (Sampson *et al.*,
 2010, character 120):
 (0) – absent.
 (1) – present.
 The coronoid process is separated from the body of the dentary in
 neoceratopsians, except for *Leptoceratops* and *Protoceratops*.

(121) Splenial, shape (Sampson *et al.*, 2010, character 121):
 (0) – nearly as deep as the body of the dentary, does not contact articular, angular
 exposed in medial view.
 (1) – shallow, contacts articular, covers angular in medial view.
 The splenial is shallow in neoceratopsians, except for *Leptoceratops* and
 *Protoceratops*.

(122) Prearticular-dentary contact (Sampson *et al.*, 2010, character 122):
 (0) – absent.
 (1) – present.
 The prearticular-dentary contact is present in neoceratopsians, except for
 *Leptoceratops* and *Protoceratops*.

Dentition

(123) Tooth, number of roots (Forster, 1990, character 34):
 (0) – one.
 (1) – two.
 All ceratopsids have double-rooted teeth; non-ceratopsid teeth are single-rooted.

(124) Tooth, number of replacements per alveolus (Sereno, 1999, character 137):
 (0) – one or two replacement teeth.
 (1) – three or more replacement teeth.
 Alveoli have three or more replacement teeth in ceratopsids, but only one or two
 in outgroup taxa. *Turanoceratops* has at least two, possibly three replacement teeth per
 alveolus, and was scored as dimorphic.
 *Turanoceratops tardabilis* was recoded from 0 to 0&1 because it has at least two
 (0), possibly three (1) replacement teeth per alveolus.

(125) Tooth magazine, case-like alveolar slots for vertical tooth families formed by spongy bone
 (Sampson *et al.*, 2010, character 125):
 (0) – absent.
 (1) – present.
 The alveolar slots for vertical tooth families is formed by spongy bone in
 neoceratopsians, except for *Leptoceratops* and *Protoceratops*.

(126) Cheek teeth (Forster, 1990, character 37):
 (0) – spaced.
 (1) – closely packed, roots abut.
 All taxa used in this analysis have closely packed teeth; *Psittacosaurus* has spaced
 teeth, but this taxon was not included in this analysis. This character is therefore
 parsimony uninformative.
 *Leptoceratops gracilis* and *Protoceratops andrewsi* were both recoded from 0 to 1
 because the cheek teeth are closely packed.

Axial skeleton

(127) Cervical vertebrae, formation of syncervical (Forster, 1990, character 122):
 (0) – C1-3 fused or tightly articulated, atlantal hypocentrum present as a ventrally placed,
 wedge-like bone.
 (1) – C1-3 firmly fused, atlantal hypocentrum forms a complete ring.
 Cervical vertebrae 1–3 are firmly fused in ceratopsids, but not in *Protoceratops*.

(128) Axis, neural spine shape and orientation (Sereno, 1999, character 141):
 (0) – blade-like and nearly vertical, overhangs only anteriormost portion of C3.
 (1) – blade-like morphology lost, spine steeply angled to reach posterior margin of C3.
 The axis neural spine is blade-like in *Leptoceratops* and *Protoceratops*, but not in
 ceratopsids.

(129) Atlantal rib (Sampson *et al.*, 2010, character 129):
 (0) – present.
 (1) – absent.
 The atlantal rib for the atlas is present in *Leptoceratops* and *Protoceratops*, but
 absent in ceratopsids.

(130) Dorsal vertebrae, shape of centra (Sampson *et al.*, 2010, character 130):
 (0) – relatively axially elongate.
 (1) – axially shortened.
 The dorsal vertebral centra are axially shortened in neoceratopsians, except for
 *Leptoceratops* and *Protoceratops*.

(131) Sacrum, longitudinal sulcus on ventral surface (Lehman, 1989; Sereno, 1999, character
 144):
 (0) – absent.
 (1) – present.
 A longitudinal sulcus is present on the ventral surface of the sacrum in
 neoceratopsians, except for *Leptoceratops* and *Protoceratops*.

Pectoral girdle and forelimb

(132) Scapula, relative contribution to glenoid fossa (Sereno, 1999, character 145):
 (0) – scapula and coracoid contribute equally.
 (1) – scapula contributes well over half of the glenoid.
 The scapula contributes well over half of the glenoid fossa in ceratopsids, but only
 half in outgroup taxa.

(133) Scapula, orientation of scapular spine (Sampson *et al.*, 2010, character 133):
 (0) – obliquely across blade.
 (1) – longitudinally along blade.
 The scapular spine is oriented obliquely across the blade of the scapula in
 neoceratopsians, except for *Triceratops*.

(134) Olecranon process (Forster, 1990, character 104, modified)
 (0) – relatively small.
 (1) – enlarged (>one-third of ulnar length).
 In chasmosaurines, the olecranon process is either relatively small
 (*Chasmosaurus*) or large (*Triceratops*). This process is large in centrosaurines
 (*Centrosaurus*), but small in outgroup taxa.

(135) Clavicle (Sereno, 1999, character 147):
 (0) – present.
 (1) – absent.
 The clavicle is absent in ceratopsids, but present in the outgroup taxa
 *Protoceratops* and *Leptoceratops* (also present in *Psittacosaurus* – not included in
 analysis).

(136) Manual and pedal unguals, shape (Chinnery & Weishampel, 1998, character 64):
 (0) – taper to distal tip.
 (1) – dorsoventrally flattened with blunt and rounded distal tips.
 The manual and pedal unguals have blunt and rounded tips in ceratopsids, but
 taper to the distal tip in the outgroup taxa *Leptoceratops* and *Protoceratops*.

(137) Manual and pedal penultimate phalanges, shape (Sampson *et al.*, 2010, character 137):
 (0) – length exceeds width.
 (1) – width exceeds length.
 The manual and pedal penultimate phalanges are wider than long in ceratopsids,
 but are longer than wide in outgroup taxa.

Pelvic girdle and hind limb

(138) Ilium, lateral eversion of dorsal margin (Sampson *et al.*, 2010, character 138):
 (0) – absent.
 (1) – present.
 The lateral eversion of the dorsal margin of the ilium is present in ceratopsids, but
 absent in outgroup taxa.

(139) Ilium, relative lengths of pubic and ischial peduncles (Sampson *et al.*, 2010, character
 139):
 (0) – pubic and ischial peduncles long, extend well below body of ilium approximately
 the same distance.
 (1) – ischial peduncle reduced along ventral aspect, pubic peduncle projects further
 ventrally than ischial peduncle.
 The pubic peduncle projects further ventrally than the ischial peduncle in
 ceratopsids, but both peduncles are equal in length in outgroup taxa.

(140) Pubis, prepubic process (Forster, 1990, character 111):
 (0) – short and unexpanded distally.
 (1) – elongate, distal end greatly expanded dorsoventrally.
 The prepubic process is elongate in ceratopsids, but short in outgroup taxa. This
 character was left unquantified, as it does not affect the outcome of the analysis.

(141) Pubis, position and length of postpubic rod (Forster, 1990, character 110):
 (0) – relatively short, but extends past ischial peduncle of ilium, arises ventral to
 acetabulum and lies along ventral and ventromedial margin of ischium.
 (1) – very abbreviated, terminates at level of ischial peduncle, arises medial to
 acetabulum and passes entirely medial to ischium.
 The postpubic rod terminates at the level of the ischial peduncle in ceratopsids,
 but extends past the ischial peduncle in outgroup taxa. This character was left
 unquantified, as it does not affect the outcome of the analysis.

(142) Pubis and ischium, morphology of contributions to acetabulum (Sampson *et al.*, 2010,
 character 142):
 (0) – pubic acetabular surface faces posterolaterally, pubis and pubic process of ischium
 contribute equally to ventral margin of acetabulum.
 (1) – pubic acetabular surface faces laterally and forms a partial medial wall to the
 acetabulum, pubic process of ischium elongate and meets pubis close to anterior
 margin of acetabulum, ventral portion of pubic acetabular surface lies medial to
 pubic ramus of ischium.
 The pubic acetabular surface faces laterally in ceratopsids, but posterolaterally in
 outgroup taxa.

(143) Ischium, cross-sectional shape of shaft (Forster, 1990, character 112):
 (0) – thick and ovoid.
 (1) – laterally compressed and bladelike, tapered dorsally.
 The cross-sectional shape of the ilium shaft is laterally compressed in
 chasmosaurines, but thick and ovoid in centrosaurines and outgroup taxa.

(144) Ischium, orientation of shaft (Forster, 1990, character 113):
 (0) – nearly straight or slightly decurved.
 (1) – broadly and continuously curved.
 The ischium shaft is broadly curved in neoceratopsians, except for *Leptoceratops*
 and *Protoceratops*.

(145) Femur, morphology of greater and lesser trochanters (Dodson *et al.*, 2004, character 72):
 (0) – trochanters distinct and located below the level of the femoral head.
 (1) – trochanters coalesced and level with the femoral head.
 The greater and lesser trochanters of the femur are coalesced in ceratopsids, but
 distinct in outgroup taxa.

(146) Femur, size of fourth trochanter (Sereno, 1999, character 154):
 (0) – large and pendant.
 (1) – small, reduced to low prominence.
 The fourth trochanter of the femur is small in ceratopsids, but large in outgroup
 taxa.

(147) Femur-tibia proportion (Forster, 1990, character 103):
 (0) – tibia longer than femur.
 (1) – femur longer than tibia.
 The femur is longer than the tibia in ceratopsids, but the tibia is longer than the
 femur in outgroup taxa.

(148) Pes, metatarsal proportions (Sampson *et al.*, 2010, character 148):
 (0) – length of MT I two-thirds the length of MT II.
 (1) – MT I reduced to one half or less the length of MT II.
 Metatarsal I is half or less the length is metatarsal II in ceratopsids, but metatarsal
 I is two-thirds the length of metatarsal II in outgroup taxa.

Lower jaw

(149) Lateral ridge of dentary (Mallon *et al.*, 2011, character 149):
 (0) – present.
 (1) – absent.
 The lateral ridge of the dentary is absent in chasmosaurines, except for
 *Anchiceratops* and *Arrhinoceratops*. This feature is dimorphic in *Centrosaurus* and
 *Pachyrhinosaurus*. In outgroup taxa, it is dimorphic in *Leptoceratops*, present in
 *Protoceratops*, and absent in *Zuniceratops*.

Dermal Skull Roof
(150) Quadratojugal-squamosal contact in lateral view (Wick & Lehman, 2013, character 150):
 (0) – quadratojugal overlapped by squamosal dorsally.
 (1) – quadratojugal bifurcated with processes dorsal and ventral to squamosal.
 The quadratojugal is overlapped by the squamosal in most chasmosaurines (but
 bifurcated in *Bravoceratops* and dimorphic in *Torosaurus* *utahensis*, *Triceratops
 horridus* and *Triceratops prorsus*) and centrosaurines. Taxa either missing (not
 preserved) the quadratojugal or without a contact between the quadratojugal and
 squamosal were coded here as “?”.
 *Leptoceratops* and *Protoceratops* were recoded from 0 to ? because the
 quadratojugal and squamosal do not contact each other in these taxa.

(151) Shape of the nasal bridge in dorsal view (Wick & Lehman, 2013, character 151):
 (0) – wide posterior to the nasal horncore.
 (1) – constricted posterior to the nasal horncore.
 The nasal bridge is wide posterior to the nasal horncore in all ceratopsids, except
 for *Bravoceratops*.

(152) Nasal horncore horizontal cross-section (Mallon *et al.*, 2014, character 152):
 (0) – ellipse- or teardrop-shaped.
 (1) – triangular (horncore with flattened anterior face and distinct posterior keel)
 The horizontal cross-sectional shape of the nasal horncore is ellipse- or teardrop-
 shaped in all ceratopsids, except for *Arrhinoceratops* and *Eotriceratops*.

Epiossifications on parietosquamosal frill

(153) Epiparietal P4 shape (new):
 (0) – low raised D-shaped process.
 (1) – strongly recurved triangular or recurved low gnarled triangular process.
 (2) – well-developed triangular process.
 (3) – elongate, low process.
 In chasmosaurines, epiparietal 4 is low D-shaped (*Chasmosaurus* – YPM 2016),
 strongly recurved triangular (*Vagaceratops* – CMN 41357 and TMP 1987.045.0001),
 well-developed triangular (*Anchiceratops*), or an elongate low process (*Torosaurus*).
 Epiparietal 4 is a low raised D-shaped process in *Centrosaurus*, *Albertaceratops* and
 *Pachyrhinosaurus*.

(154) Epiparietal, P4 orientation (new):
 (0) – epiparietal oriented in the plane of the frill.
 (1) – directed anterodorsally.
 In *Chasmosaurus*, epiparietal 4 is anterodorsally oriented (e.g., YPM 2016). In
 other chasmosaurines, epiparietal 4 is oriented in the plane of the frill (*Torosaurus* and
 *Anchiceratops*) or anterodorsally oriented (CMN 41357 and TMP 1987.045.0001,
 *Vagaceratops*). Epiparietal 4 is posterolaterally oriented in *Centrosaurus*,
 *Albertaceratops* and *Pachyrhinosaurus*.

(155) Epiossification in P5 position, shape (new):
 (0) – low raised D-shaped process.
 (1) – well-developed triangular process.
 (2) – elongate low process.
 In chasmosaurines, epiparietal 5 is a well-developed triangular process in
 *Chasmosaurus* (YPM 2016) and *Vagaceratops* (CMN 41357), and an elongate low
 process in *Torosaurus*. Epiparietal 5 is a low raised D-shaped process in *Centrosaurus*,
 *Albertaceratops* and *Pachyrhinosaurus*. An epiparietosquamosal situated lateral to
 epiparietal 4 occurs in *Vagaceratops* (TMP 1987.045.0001). Mallon *et al.* (2011) noted
 that the position of the fourth epiossification from the parietal midline was variable in
 *Anchiceratops ornatus*, either bridging the parietal-squamosal contact
 (epiparietosquamosal = 3 epiparietals per side; e.g., AMNH 5273 and UALVP 1618) or
 articulated only with the parietal (epiparietal = 4 epiparietals per side; e.g., CMN 8535
 and UW 2419). They noted that *A. ornatus* skulls possessing an epiparietosquamosal (3
 epiparietals per side) are generally smaller and less mature than *A. ornatus* skulls with an
 epiparietal (4 epiparietals per side), which are generally larger and more mature. They
 suggested that the epiparietosquamosal migrates medially over ontogeny, becoming
 articulated entirely with the parietal. The epiparietosquamosal and epiparietal 4 therefore
 appear to be homologous in *A. ornatus*. The epiparietosquamosal and epiparietal 5 are
 likewise interpreted here as being homologous in *Chasmosaurus* (YPM 2016, epiparietal)
 and *Vagaceratops* (TMP 1987.045.0001 and CMN 41357, respectively), that may or may
 not be related to ontogeny.

Table A. Character-taxon matrix used for phylogenetic analysis (modified after Campbell (2015)).

Taxon 10 20 30 40

*Leptoceratops gracilis* 100??0???0 ??0??01000 1?001?0??0 00000?????
*Protoceratops andrewsi* 000??0???0 ??0??01000 1100001000 00000?????
*Zuniceratops christopheri* 111000???0 ??0??0?010 000?100??0 ?111100010
*Turanoceratops tardabilis* ?????????? ?????????? ???0?0???? ????1?0110
*Albertaceratops nesmoi* ?111?0???0 ??0??????0 1?1011110? ?11?1?0011
*Centrosaurus apertus* 011110?0?0 ??0??11100 1?11111101 0111100002
*Pachyrhinosaurus lakustai* 011110?0?0 ??0??11100 1?11111201 0111111???
*Kosmoceratops richardsoni* 1110011111 0011000010 0011111100 0111110111
*Agujaceratops mariscalensis* 1110010101 1010000011 1?11111100 0111110110
*Utahceratops gettyi* 1110010111 0?11000011 0011111100 0111111102
*Pentaceratops sternbergi* 1110010111 1111000011 0011111100 0111110011
*Coahuilaceratops magnacuerna* 11100?1??1 0????00??1 ??11111110 ??1?11??11
*Anchiceratops ornatus* 1110011[01]11 1011000100 1?11111100 0111111111
*Arrhinoceratops brachyops* 1110011??1 ?0100001?0 1?11111100 01111?1111
*Ojoceratops fowleri* 11??0????? ?????????0 ??????111? ?1????????
*Torosaurus latus* 1110010??1 11???0010? 1?11111110 0111111[01]11
*Torosaurus utahensis* ?????????? ?????????? ?????????? ???11?1011
*Eotriceratops xerinsularis* 11100100?1 1011000??0 ??111111?? 011?111?11
*Nedoceratops hatcheri* 11100100?1 1111100?0? 1?111?1110 011?1?1011
*Triceratops horridus* 11100100?1 1111100100 1?11111110 0111111011
*Triceratops prorsus* 11100100?1 1111100100 1?11111110 0111111011
*Bravoceratops polyphemus* ?1???????? ?????????? ??????111? ????111?1?
*Judiceratops tigris* ?????????? ?????????? ?????????? ????1101?0
AMNH 5401 1110010101 0010000010 00111?1100 11111?0110
AMNH 5402 1110010101 ?010000?10 1?111?1100 11111?000?
AMNH 5656 ?????????? ?????????? ?????????? 1?????????
CMN 0491 ?????????? ?????????? ?????????? ??????????
CMN 1254 ?1??0????? ?????????0 ??????1100 ?11?110010
CMN 2245 ?????????? ?????????? ??????11?? ?1111?0002
CMN 2280 1110010101 ?010000010 1?11111100 1111110000
CMN 8800 1110010101 ?010000?1? 00111?1100 1111110???
CMN 41357 111001?101 0010000010 ??1?111100 1111??????
NHMUK R4948 1110010101 ?010000??0 ??11111100 ?111110???
ROM 839 1110010101 0010000010 1?11111100 11111?0000
ROM 843 ?1100??1?? ??????0010 1?11111100 11111?0???
TMP 1981.019.0175 1110010101 0010000010 1?11111100 111111010?
TMP 1983.025.0001 ?1???????? ?????????? ?????????? ?11111011?
TMP 1987.045.0001 111001?101 0010000??0 1?11111100 ?11???????
UALVP 40 1110010101 0010000010 1?11111100 11111?0100
YPM 2016 1110011101 0010000?10 1?11111100 1111110???

Table A. Character-taxon matrix used for phylogenetic analysis (modified after Campbell (2015)). (Continued)

Taxon 50 60 70 80

*Leptoceratops gracilis* ?000?00000 00000?00?? 000000?000 00000?????
*Protoceratops andrewsi* ?000?00000 00000?0100 110000?000 0010000000
*Zuniceratops christopheri* 2111011?0? ?11?0????? ??0??????? ?01?01000?
*Turanoceratops tardabilis* 2????????? ??1?0????? ?????????? ??????????
*Albertaceratops nesmoi* 2110?11112 111???1100 111001011? 1011110011
*Centrosaurus apertus* 2110?11112 1111101100 1110010110 1011110311
*Pachyrhinosaurus lakustai* ?111111112 ?111101100 1110010110 1011110311
*Kosmoceratops richardsoni* 1111110110 1111011112 0010111211 1011100010
*Agujaceratops mariscalensis* 0111110110 1111??111? 1011112?11 ??1110?201
*Utahceratops gettyi* 2111110110 ?111111111 1011112111 1011101201
*Pentaceratops sternbergi* 211111[01]111 1111111111 1011112211 1011101201
*Coahuilaceratops magnacuerna* ?1???????? ???????11? ?01?1????? ?01?1??201
*Anchiceratops ornatus* 1111110110 1111111112 101110?111 10111100[01]1
*Arrhinoceratops brachyops* 1111110110 1111[01]11111 10111[01]0111 1011110011
*Ojoceratops fowleri* ??111????? ???????112 0?101????? ????1100??
*Torosaurus latus* 2111110112 111?[01]11111 101110?[01]12 1010110312
*Torosaurus utahensis* 2111110112 ?1??1??11? 101110?012 1010110312
*Eotriceratops xerinsularis* 21?111?110 ???????11? 1???1????? ??????????
*Nedoceratops hatcheri* 2?11110?12 ?1?1111111 1?101???02 1?11110311
*Triceratops horridus* 2111110112 1111111111 101010?002 11001?????
*Triceratops prorsus* 2111110112 1111011111 101010?002 11001?????
*Bravoceratops polyphemus* ??????0?11 1?????111? ??1?1???1? ?01?1??301
*Judiceratops tigris* 2?11??1?1? ??1????111 1?11?0?0?? ?01?11041?
AMNH 5401 2111111110 1111111111 10?11???12 101110?001
AMNH 5402 2111111112 1111111111 10?110??12 1011100002
AMNH 5656 ?????1??12 1???1?1111 10?1111112 ?011100101
CMN 0491 ?????????? ?????????? ?????11??? ?01?10010?
CMN 1254 21111????? ??1111?111 10111???1? ?01???????
CMN 2245 211111111? 1111111111 1011111112 1011100101
CMN 2280 2111111112 1111111111 1011111110 1011100101
CMN 8800 ?1111??1?? ?111111111 10?1112112 1011100101
CMN 41357 ?111111?12 1111111111 101110??12 ?011100000
NHMUK R4948 ?111111112 11111?1111 1011111112 ?01?100101
ROM 839 2111111110 1111111111 10111???12 1011100101
ROM 843 ?111111110 1111111111 1011111210 1011100102
TMP 1981.019.0175 2111111112 111111111? ?0111???1? 10111??10?
TMP 1983.025.0001 2111111112 1111111111 1011111110 1011100201
TMP 1987.045.0001 ?111111112 1111111111 10111???12 101110???0
UALVP 40 2111111112 11111?111? ?0111???1? ?0111?????
YPM 2016 ?111111112 1111111111 101110??12 1011100102

Table A. Character-taxon matrix used for phylogenetic analysis (modified after Campbell (2015)). (Continued)

Taxon 90 100 110 120

*Leptoceratops gracilis* ?000?????? ?????????? ???000?000 0000000000
*Protoceratops andrewsi* 0000?????? ?????????? ???0000000 0000000000
*Zuniceratops christopheri* ???0?????? ?????????? ????11?1?? ?1?0011011
*Turanoceratops tardabilis* ?????????? ?????????? ?????????? ????0?????
*Albertaceratops nesmoi* 11110?0??? 101000???? ?10??1?1?? ?1?11011?1
*Centrosaurus apertus* 11110?0000 1010011132 1001111111 1111101111
*Pachyrhinosaurus lakustai* 1111??0??? 1?1000???2 1101011111 1?11101111
*Kosmoceratops richardsoni* 01[01]1111321 1101012132 221??????? ?11?011?11
*Agujaceratops mariscalensis* 11011?111? 0???01???? ???111111? ??110?1111
*Utahceratops gettyi* 1101111221 0?00013133 030???1111 ?111011111
*Pentaceratops sternbergi* 1101111221 0?00013133 030?111111 1111011111
*Coahuilaceratops magnacuerna* 1101??1??? ?????????? ?????????? ??1?011111
*Anchiceratops ornatus* 0101111220 [01]2[01]00132330301111111 11?1?01?11
*Arrhinoceratops brachyops* 01011?1110 1200014004 0401111111 1111101111
*Ojoceratops fowleri* ?1011?1110 ??0011400? ?40??????? ?111011?11
*Torosaurus latus* 01011?1110 0?10014004 0401111111 11?1??????
*Torosaurus utahensis* 01011?1111 1210114004 040??????? ?111?11111
*Eotriceratops xerinsularis* ?1011?1??? 12???????? ?????????? ??????????
*Nedoceratops hatcheri* 01011?1110 1????1???? ??????111? ??????????
*Triceratops horridus* ?1011?1110 1200114004 0401111111 1111011111
*Triceratops prorsus* ?1011?1110 1200114004 0401111111 1111011111
*Bravoceratops polyphemus* 11011?1??? ????1??2?? ???1111??? ????????11
*Judiceratops tigris* ?1011?1??? ?????????? ?????????? ??????????
AMNH 5401 11011?1?10 0????????? ????11?1?1 ??????????
AMNH 5402 11010?1?10 0?00010000 030?11?1?1 ?1110?1111
AMNH 5656 11011?1?10 ??00013003 0?0?????1? ??????????
CMN 0491 1101?????? ????01010? ?????????? ??????????
CMN 1254 1101?????? ?????????? ?????????? ?11???1?11
CMN 2245 11010?1110 0?0001?1?? ?30111???? ?111011111
CMN 2280 11011?1110 0?00013103 030?11???1 ?1110?1111
CMN 8800 11011?1110 0?00010100 030??????1 ??????????
CMN 41357 11011?1110 0?11012132 221??????1 ?111011111
NHMUK R4948 11011?1110 0????????? ??????1??1 ??110???11
ROM 839 11011?1110 0??0?????? ???111?111 ?111011111
ROM 843 11011?1110 0?00010103 ?30?11?111 ?11???1111
TMP 1981.019.0175 11010?1??? ?????????? ???111?111 ??????????
TMP 1983.025.0001 11010?1110 0?00013003 030??????? ??????????
TMP 1987.045.0001 11011?1110 12???????? ????11???1 ??????????
UALVP 40 ?1010?1??? ?????????? ???111?1?1 ??110?1?11
YPM 2016 11011?1110 0?10010100 001111?1?1 ??????????

Table A. Character-taxon matrix used for phylogenetic analysis (modified after Campbell (2015)). (Continued)

Taxon 130 140 150 155

*Leptoceratops gracilis* 000001?000 000000000? 0?000000[01]? 0????
*Protoceratops andrewsi* 0000010000 0000000000 000000000? 0????
*Zuniceratops christopheri* ??001????1 100??????? ??01?0??1? ?????
*Turanoceratops tardabilis* ??1[01]11???? ?????????? ?????????? ?????
*Albertaceratops nesmoi* ???111???? ?????????? ?????????0 0?000
*Centrosaurus apertus* 1111111111 1101111111 11011111[01]0 00000
*Pachyrhinosaurus lakustai* 11111111?1 ?10??11111 110111??[01]0 0?000
*Kosmoceratops richardsoni* 1?1?11???? ????1????? ????????10 00???
*Agujaceratops mariscalensis* ??1?111111 110????111 ?11111??10 00???
*Utahceratops gettyi* 111111???1 ?10??????1 ??11111?10 00???
*Pentaceratops sternbergi* 1111111111 110?111111 1111?11??0 00???
*Coahuilaceratops magnacuerna* 1???11???? ?10??????? ????????1? 00???
*Anchiceratops ornatus* ???111???? ?????????? ????????00 0020?
*Arrhinoceratops brachyops* 11??111?1? ?1???????? ????????00 01???
*Ojoceratops fowleri* 1???11???? ??0??????? ????????1? 00???
*Torosaurus latus* ??1111???1 ?10??????? ?????????[01] 00302
*Torosaurus utahensis* ??1?111?11 ?????????? ????????1? ??302
*Eotriceratops xerinsularis* ????1111?? ?????????? ?????????? ?1???
*Nedoceratops hatcheri* ?????1???? ?????????? ?????????? 00???
*Triceratops horridus* 1111111111 1111111111 111111111[01] 00???
*Triceratops prorsus* 1111111111 ??1??????? ???????11[01] 00???
*Bravoceratops polyphemus* ?????????? ?????????? ????????11 10???
*Judiceratops tigris* ?????????? ?????????? ?????????? ?????
AMNH 5401 ????11???? ?????????? ?????????0 00???
AMNH 5402 11??11???? ?????????? ????????10 00???
AMNH 5656 ????11???? ?????????? ?????????? 0????
CMN 0491 ?????????? ?????????? ?????????? ?????
CMN 1254 111??????? ?????????? ????????1? ?????
CMN 2245 1111111111 11001??1?1 ?1??111110 00???
CMN 2280 1111111111 110?1??111 11111???10 00???
CMN 8800 ?????????? ?????????? ?????????? ?0???
CMN 41357 11?1111111 ?10?1111?? ?11??11?10 00111
NHMUK R4948 ??1?111111 11?01????1 ????111??0 00???
ROM 839 111111???? ?????????? ????????10 00???
ROM 843 11111111?1 ?1001111?1 ?111111?10 00???
TMP 1981.019.0175 ??1111???? ?????????? ?????????0 00???
TMP 1983.025.0001 ?????????? ?????????? ?????????0 0????
TMP 1987.045.0001 ???111???? ?????????? ?????????0 00111
UALVP 40 ??1111???? ?????????? ????????10 00???
YPM 2016 ??1111???? ?????????? ?????????0 00011
