# Supplementary material for: A Re-Evaluation of the Chasmosaurine Ceratopsid Genus Chasmosaurus (Dinosauria: Ornithischia) from the Upper Cretaceous (Campanian) Dinosaur Park Formation of Western Canada
Source: PLoS One. 2016 Jan 4;11(1):e0145805. doi: 10.1371/journal.pone.0145805 (PMC4699738; doi:10.1371/journal.pone.0145805)
Supplement: S5 File — (DOC) [file pone.0145805.s005.doc]

**Supplementary Material 5. Ontogenetically variable characters used in phylogenetic analysis.**

Character 8 – Premaxilla, septal flange:
 The presence of this flange appears to vary ontogenetically in *Triceratops*, being present (1) in immature specimens, but absent (0) in mature specimens (Scannella *et al.*, 2013).

Character 35 – Postorbital, postorbital ornamentation in adult:
 Holmes *et al.* (2001) originally diagnosed *Vagaceratops* *irvinensis* as lacking postorbital horncores (0); however, they left open the possibility that the rugose and pitted mound present on the dorsal surface of the postorbital in CMN 41357 and TMP 1987.045.0001 may represent the base of a remodeled and resorbed horncore (1). The *Vagaceratops*-like skulls AMNH 5402 and YPM 2016 were recovered in this study as relatively immature and mature adults, respectively, and possess complete postorbital horncores and pitted mounds, respectively. This suggests that *Vagaceratops*-like specimens possessed postorbital horncores earlier in life, but were subsequently lost through remodeling later in life. Postorbital horncore remodeling also occurs in *Chasmosaurus*-like specimens, as evidenced by specimens possessing both a complete or partially complete horncore on one side of the skull and a pitted mound on the other (e.g. CMN 2280, CMN 8800, and TMP 1981.019.0175).

Character 36 – Postorbital, extent of cornual sinus in base of postorbital ornamentation:
 The extent of the cornual sinus into the postorbital is known to change ontogenetically within *Triceratops* (Farke, 2006). This sinus expands into the base of the postorbital horncore over ontogeny, and eventually excavates the shaft of the horncore.

Character 39 – Postorbital, length of postorbital horncore:
 In *Triceratops* ontogeny, postorbital horncores are relatively short (0, less than 15% basal skull length) early in life, and lengthen (1, greater than 35% of basal skull) later on (Horner & Goodwin, 2006).

Character 40 – Postorbital, curvature of postorbital horncore in lateral view:
 The orientation of the postorbital horncores in lateral view is known to change ontogenetically in *Triceratops*, being posteriorly curved (0) early in life, straightening out (2), and becoming anteriorly curved (1) later on (Horner & Goodwin, 2006).

Character 50 – Epijugal length:
 A decrease in the length of the epijugal relative to its basal width may be partly ontogenetic in *Chasmosaurus*-like specimens, with epijugals being relatively long (0) in some relatively less mature specimens (i.e. AMNH 5401, ROM 839, and ROM 843), and relatively short (2) in relatively more mature specimens (e.g. CMN 2280 and TMP 1981.019.0175).

Character 55 – Frontoparietal fontanelle leading into supracranial cavity complex:
 The absence (0) and presence (1) of a frontoparietal fontanelle in both *Triceratops* and *Torosaurus* is thought to related to ontogeny, with the gradual closure of the fontanelle due to bone overgrowth (Farke, 2010).

Character 56 – Frontoparietal fontanelle, shape:
 The shape of the frontoparietal fontanelle changes as a result of bone overgrowth in some taxa (*Triceratops* and *Torosaurus*; Farke, 2010), and is hence related to ontogeny.

Character 73 – Parietal fenestra:
 In their *Triceratops*-*Torosaurus* synonymy, Scannella & Horner (2010) propose that the formation of parietal fenestrae is ontogenetic with the unfenestrated (0) and putatively immature *Triceratops* maturing into a fenestrated (1) and putatively mature *Torosaurus*. The formation of parietal fenestrae is also ontogenetic in the basal ceratopsian *Protoceratops* (Fastovsky *et al.*, 2011).

Character 75 – Parietal, sharp median crest:
 In *Triceratops* (Goodwin *et al.*, 2006) and *Centrosaurus* (Ryan *et al.*, 2001; Brown *et al.*, 2009) ontogeny, this crest is present (0) early in life, but gradually becomes less pronounced (1) later in ontogeny.

Character 82 – Parietosquamosal frill, marginal undulations:
 These undulations refer to the scalloped free margin of the parietosquamosal frill, and correspond to epiossification (episquamosal and epiparietal) attachment sites. In ceratopsids, these undulations are most pronounced in relatively immature individuals, but become less pronounced later in maturity and are difficult to discern underneath the articulated epiossifications; these undulations do not disappear entirely, however, as they serve as an attachment site for epiossifications throughout maturity.

Character 84 – Marginal dermal ossifications on parietosquamosal frill:
 In ceratopsids, parietosquamosal frill dermal ossifications (episquamosals and epiparietals) are disarticulated from the frill and absent (0) early in life, but are present (1) and articulated with the frill later on (Horner & Goodwin, 2006).

Character 91 – Epiparietosquamosal, marginal ossification crossing squamosal-parietal contact:
 The *Vagaceratops*-like skull AMNH 5402, the inferred fifth epiossification from the midline of the parietal is fused entirely onto the parietal, although the lateral end of the base of this epiossification overhangs the distal end of the squamosal. In chasmosaurine ontogeny, the entire base of each epiparietal and episquamosal fuses onto the frill margin (Horner & Goodwin, 2006). If the epiossification (epiparietal) in AMNH 5402 had undergone complete articulation with the underlying frill margin, it would have partly articulated with the squamosal as well, making it an epiparietosquamosal. Epiparietosquamosals, where present in Dinosaur Park Formation chasmosaurine skulls (AMNH 5402 and TMP 1987.045.0001, and likely TMP 1998.102.0008), are interpreted as being ontogenetic, going from absent (0) to present (1).

Character 93 – Epiparietals, number per side:
 Epiparietal count is relatively conservative amongst chasmosaurines, with all but three genera having three per side. *Anchiceratops* has three to four, *Vagaceratops*-like specimens have four to five, and *Torosaurus* has five to six per side. Mallon *et al.* (2011) found that in *Anchiceratops*, specimens with three epiparietals and one epiparietosquamosal per side were relatively immature and those with four epiparietals per side were more mature. They reasoned that the epiparietosquamosal migrates laterally over ontogeny and becomes articulated exclusively with the parietal, becoming an epiparietal. It is likely that epiparietal count changes from five to four in amongst some members of the *Vagaceratops*-like operative taxonomic unit, as suggested by AMNH 5402 (see character 91 above).

Characters 97, 100, and 102 – Shape of epiparietal 1, 2 and 3, respectively:
 Epiparietal 1, 2, and 3 are well-developed triangular processes (3) in immature specimens of *Triceratops*, but they remodel into elongate low processes (4) later in maturity (Horner & Goodwin, 2006).

Character 98 – Epiparietal, locus P1 orientation:
 P1 is oriented in the plane of the frill (0) in relatively immature Dinosaur Park Formation chasmosaurine specimens, but becomes anterodorsally oriented (1) later in life. This is due to the reorientation of the underlying parietal margin over ontogeny, as it develops into an anterodorsally thickened ridge.

Character 103 – Epiparietal, locus P3 orientation:
 Epiparietal 3 is not preserved in the relatively immature *Vagaceratops*-like specimen AMNH 5402. However, given that the inferred parietal loci is oriented in the plane of the frill, it is likely that this epiparietal would have also been oriented in the plane of the frill (0). Epiparietal 3 is oriented anterodorsally (1) in the more mature member *Vagaceratops*-like skull YPM 2016.

Character 105 – Supraoccipital, contribution to foramen magnum:
 In *Triceratops* ontogeny, the supraoccipital forms the dorsal margin of the foramen magnum (0) early in life, but this element is eliminated from the foramen magnum (1) by the exoccipitals later in life (Goodwin *et al.*, 2006).

Character 149 – Lateral ridge of dentary:
 The presence of this ridge appears to vary ontogenetically in *Triceratops*, being present (0) in immature specimens, but absent (1) in mature specimens (Scannella *et al.*, 2013).

Character 152 – Epiparietal, locus P4 orientation:
 The ontogenetic nature of this character follows the same reasoning as described above for character 103, involving AMNH 5402 (0) and YPM 2016 (1).
