# Supplementary material for: A Re-Evaluation of the Chasmosaurine Ceratopsid Genus Chasmosaurus (Dinosauria: Ornithischia) from the Upper Cretaceous (Campanian) Dinosaur Park Formation of Western Canada
Source: PLoS One. 2016 Jan 4;11(1):e0145805. doi: 10.1371/journal.pone.0145805 (PMC4699738; doi:10.1371/journal.pone.0145805)
Supplement: S7 File — (DOC) [file pone.0145805.s007.doc]

**Supplementary Material 7. List of characters used in ontogenetic analysis.**

**Introduction**

Ontogenetically-variable characters are inferred in this analysis based on the presence of a particular character state amongst smaller skulls relative to another state of the same character present in larger skulls. This inference is also based on sequences of changes which can only progress in one direction (e.g. articulation of epiossifications or other cranial elements). For any given character (e.g. character 2, articulation of epinasal with nasals), the presence of a particular character state (epinasal disarticulated) in relatively small skulls was considered as the immature/basal condition, and coded as “0”. Likewise, the presence of a different state of the same character (epinasal articulated) in larger skulls was considered as the mature condition, and coded as “1”. The size-related states in each of the 18 characters used in this analysis have also been shown to be size-related in other ceratopsid taxa, where noted. In the case of eight characters (1, 2, 4, 11, 12, 14, 15, 17), a third state (2) was added to capture the range of ontogenetic variation seen amongst the specimens. These eight characters were ordered in the analysis. The specimen-character matrix used in this analysis is listed in Table A.
 Rostral-to-epijugal length (parameter 16) was used as a baseline for skull size in this study as it approximates basal skull length, rostral-to-occipital condyle, as used in other studies (e.g. Dodson, 1976; Mallon *et al.*, 2011), but is an easier measurement to obtain than the latter given the incomplete nature of many of the skulls. Measurements for the currently inaccessible skulls AMNH 5401 and AMNH 5402 were obtained from Godfrey & Holmes (1995); their study included rostral-to-epijugal length, but not rostral-to-occipital condyle length. CMN 8800 and CMN 8801 are both partly encased in plaster or matrix, rendering the occipital condyles inaccessible. CMN 41357 does not preserve the braincase, making a rostral-to-occipital condyle measurement impossible. In this study, *Chasmosaurus* specimens were designated as small or large based on the following criteria: small specimens are skulls with (or estimated as having) rostral-to-epijugal lengths of up to 628 mm; e.g. UALVP 40); large skulls have (or are estimated as having) rostral-to-epijugal lengths of 680 mm; e.g. AMNH 5402, or greater.
 Articulated fossilized cranial elements can be held together by: 1, sediment, that has sometimes replaced connective tissue which once held the elements together, but decayed after burial; 2, an interdigitating; or, 3, coalesced contact between them. The fact that most Dinosaur Park Formation chasmosaurine crania were discovered as isolated units (e.g. AMNH 5401, AMNH 5402, AMNH 5656, CMN 0491, CMN 8800, CMN 8802, CMN 8803, CMN 34829, TMP 1981.019.0175, TMP 1983.025.0001, TMP 1998.102.0008, TMP 2009.034.0009, and YPM 2016) suggests that connective tissue decay between skull and body occurred largely before burial. A missing rostral or cranial epiossification (i.e. epinasal, epijugal, episquamosal, and epiparietal) was either not present or formed before death, or represents an element that was lost post mortem. As it is difficult to determine if two closely associated elements are truly fused through the comingling of their boney tissues, or simply held in place by minerals or sediment, such elements are referred to here as 'articulated' unless a portion of their contacting surfaces have been obliterated and are obviously fused. Degree of fusion can then be designated on an element-by-element basis based on the degree of obliteration of connection surfaces. Two articulating elements are considered to exhibit an early stage of articulation if their sutures are visible or open externally. An advanced stage of articulation is indicated if their sutures are obliterated or closed externally.

**List of characters**

(1) Rostral and premaxilla, articulation (Longrich & Field, 2012; character 23, modified):
 (0) – rostral disarticulated.
 (1) – articulated, but suture between dorsal process of rostral and dorsal margin of
 premaxillae open in dorsal view.
 (2) – articulated, with suture between dorsal process of rostral and dorsal margin of
 premaxillae closed in dorsal view.

The rostral is assumed to be a disarticulated element in immature ceratopsids (0),
 but is articulated with the premaxillae later in ontogeny (e.g. *Triceratops*, Horner &
 Goodwin, 2008; *Pachyrhinosaurus lakustai*, Currie *et al.*, 2008; Fig. 13). The rostral is
 known to be articulated with the skull in the smallest known ceratopsian specimens, e.g.,
 the hatchling-sized skulls of *Psittacosaurus* (Sereno, 2010) and *Protoceratops* (Fastovsky
 *et al.*, 2011), suggesting that this element articulated during their embryonic
 development. Early in ontogeny, the suture between these elements remains open when
 viewed externally (1). The suture between the dorsal process of the rostral and the dorsal
 margin of the premaxillae closes in advanced stages of articulation (2), although large
 ceratopsid skulls are known without rostra that never fused.
 These character states are exemplified by the successively larger skulls UALVP
 40 (0), AMNH 5402 (1) and YPM 2016 (2) (Fig. 14). In UALVP 40 (Fig. 14A), the
 rostral is missing (0), although one has been reconstructed in plaster. In AMNH 5402
 (Fig. 14B), the rostral is articulated with the premaxillae, but the suture between the
 dorsal process of the rostral and the dorsal margin of the premaxillae is open in dorsal
 view (1). In YPM 2016 (Fig. 14C), the rostral is articulated with the premaxillae, with the
 suture between the dorsal process of the rostral and the dorsal margin of the premaxillae
 is closed in dorsal view (2).


(2) Epinasal and nasals, articulation (Longrich & Field, 2012; character 15, modified here):
 (0) – disarticulated.
 (1) – articulated, with suture open in lateral view.
 (2) – articulated, with suture closed in lateral view.

The chasmosaurine nasal horncore is formed by the articulation of the epinasal
 with the underlying nasals (Horner & Goodwin, 2006; Fig. 11). The epinasal is a
 separate, disarticulated element (0) early in ontogeny, but later articulates with the dorsal
 margin of the nasals, retaining open sutures when viewed laterally (1); these sutures
 eventually close (2). The suture between the epinasal and nasals is not always visible
 (e.g. Fig. 11C–E), but its presence is inferred in specimens possessing a triangular
 horncore in lateral view; without an articulated epinasal, the nasals have a concave dorsal
 margin in lateral view (Fig. 11A). These character states are exemplified by CMN 1254
 (0), CMN 2245 (1) and ROM 843 (2) (Fig. 11). In CMN 1254 (Fig. 11A), the epinasal is
 preserved, but disarticulated from the underlying nasal (0). In CMN 2245 (Fig. 11B),
 articulation between the epinasal and nasal has taken place, with the suture between them
 open (1), but closed (2) in ROM 843 (Fig. 11C). Horner & Goodwin (2008) suggest that
 in *Triceratops*, the articulation of the epinasal with the nasals occurred at approximately
 the same time as articulation of the paired nasals.


(3) Nasal horncore, modification (new character):
 (0) – unmodified (smooth surface, comes to a point).
 (1) – modified (partially resorbed and displaying rugose or pitted texture).
 The nasal horncore is defined as the articulated nasals and epinasal. Following co-
 ossification of the epinasal and nasals, the dorsal surface of the horncore undergoes
 resorptive pitting, as seen on several large specimens (e.g. Fig. 11D, E). These character
 states are exemplified by ROM 843 (0), and CMN 8800 and YPM 2016 (1) (Fig. 11). In
 ROM 843 (Fig. 11C), the horncore has an unmodified, pointed apex (0). In YPM 2016
 (Fig. 11D), the horncore is truncated with a pitted apex (1). In CMN 8800 (Fig. 11E),
 only the base of the horncore is present, with the rest of the horncore being reduced to a
 pitted surface (1).
 It is proposed here that, as seen on postorbital horncores, the nasal horncore can
 also be modified by resorption, although the signal and mechanism for this is unknown
 (possibly as the result of environmental stress or calcium reclamation). Although some
 extant reptiles (Hoby *et al.*, 2010) and birds (Adkesson & Langan, 2007) have been
 documented to reduce bone mass as the result of environmental stress, complete
 remodeling of bony display structures have not been observed in extant vertebrates. Bone
 resorption has been proposed as a modifying agent in ceratopsids (Sampson *et al.*, 1997),
 but the mechanism for this process is unknown. In their study on cranial pitting and
 lesions in ceratopsids, Tanke & Farke (2007) reasoned that the commonly pitted
 postorbital horncores were likely the result of nonpathological bone resorption or some
 sort of pathology resulting from some sort of bone disease. Tanke & Farke (2007) also
 noted nasal horncore pitting on only one individual (cf. *Centrosaurus*, TMP
 1988.050.0113).
(4) Postorbital horncore, modification (new character):
 (0) – unmodified.
 (1) – partly resorbed, horncore between 80% and 30% of its original inferred length.
 (2) – resorbed to base, horncore is less than 20% its original inferred length, or has been
 completely lost.
 Postorbital horncore length varies considerably amongst specimens (e.g., Fig.
 17A, B). Like the nasal horncore, the postorbital horncores of several large, mature skulls
 exhibit resorptive pitting on their dorsal surfaces, with some expressed as a low-relief
 mound. The horncores have pointed apices (0) in putatively immature specimens; the
 horncores then underwent resorption starting at the tip and progressing to the base (1 and
 2). These character states are exemplified by AMNH 5401 and UALVP 40 (0), TMP
 1981.019.0175 (1), and CMN 8800 (1&2) (Fig. 17). In UALVP 40 (Fig. 17A) and
 AMNH 5401 (Fig. 17B), the horncores taper to a point (0). In TMP 1981.019.0175 (Fig.
 17C), the tip of the horncore is truncated and pitted (1). In CMN 8800 (Fig. 17D), both
 horncores are reduced and heavily pitted, with more of the right horncore preserved (1),
 than on the left side (2).
 This spectrum of postorbital horncore modification has also been identified in
 *Centrosaurus* (Ryan, 1992; Sampson *et al.*, 1997; Ryan *et al.*, 2001; Tanke & Farke,
 2007), *Einiosaurus* (Sampson *et al.*, 1997), *Pachyrhinosaurus lakustai* (Sampson *et al.*,
 1997; Tanke & Farke, 2007), and *Styracosaurus* (Sampson *et al.*, 1997; Tanke & Farke,
 2007) and was found to be size-related, with pitting only occurring in large and,
 presumably, fully mature adults.
 Postorbital horncores are absent in some *Chasmosaurus* specimens, being
 replaced with low-relief, rugose surfaces instead. Although these may be interpreted as
 the pitted remnant of a horncore (as is typically done in the ceratopsian literature), one
 could also argue that these specimens never developed a horncore. Some ceratopsids (e.g.
 *Pachyrhinosaurus lakustai*) only possess horncores as juveniles in horncores and
 incorporated this character into their diagnosis, although they did acknowledge that the
 pitted surface may represent the base of a resorbed horncore. Recent work by Horner &
 Goodwin (2006, 2008), Scannella & Horner (2010, 2011), and Longrich & Field (2012)
 on *Triceratops*, and related taxa suggest that the effect of ontogenetic change has been
 underappreciated in chasmosaurine systematics, and that postorbital horncore
 modification is a real and significant feature in this clade, just as it is in centrosaurines.


(5) Premaxillae, degree of sutural closure (new character):
 (0) – suture between posterodorsal margins of premaxillae open in dorsal view.
 (1) – suture between posterodorsal margins of premaxillae closed in dorsal view.
 Sutures between elements of the skull roof in ceratopsids close in mature
 individuals (Sampson *et al.*, 1997; Horner & Goodwin, 2006; Longrich & Field, 2012).
 Although bone may continue to grow after its sutural contacts are obliterated, growth
 would be restricted as bone can no longer be deposited between these sutures (Herring,
 1974).
 The dorsal margin of the premaxillae is covered by the rostral anteriorly, but is
 exposed posteriorly. The above character states are exemplified by AMNH 5401, AMNH
 5402 and UALVP 40 (0), and CMN 2280 (1) (Fig. 10). UALVP 40 (Fig. 10C, D) to
 AMNH 5402 (Fig. 10E, F) to AMNH 5401 (Fig. 10G, H), illustrate two successively
 larger specimens where the posterodorsal margins of the premaxillae become in
 progressively closer contact (0), with the suture between the posterodorsal margins of the
 premaxillae being closed (1) in CMN 2280 (Fig. 10I, J).


(6) Premaxilla and nasal, degree of sutural closure (Longrich & Field, 2012; character 24):
 (0) – suture between dorsal margins of elements open in dorsal view.
 (1) – suture between dorsal margins of elements closed in dorsal view.
 The posterodorsal processes of the premaxillae insert between the anterodorsal
 processes of the nasals, anterior to the nasal horncore. These character states are
 exemplified by AMNH 5401, AMNH 5402, CMN 1254, and UALVP 40 (0), and CMN
 2280 (1) (Fig. 10). In CMN 1254 (Fig. 10A, B), the sutural contact for the right
 premaxilla is present on the preserved right nasal, suggesting that sutural closure did not
 occur between these elements (0). In UALVP 40 (Fig. 10C, D), AMNH 5402 (Fig. 10E,
 F) and AMNH 5401 (Fig. 10G, H), the suture between these elements is open in dorsal
 view (0). In CMN 2280 (Fig. 10I, J), the suture between these elements is closed in dorsal
 view (1).


(7) Nasals, degree of sutural closure (Longrich & Field, 2012; character 16):
 (0) – suture open in dorsal view.
 (1) – suture closed in dorsal view.
 These character states are exemplified by AMNH 5402, CMN 1254 and UALVP
 40 (0), and CMN 2280 (1) (Fig. 10). On the preserved right nasal of CMN 1254 (Fig.
 10A, B), the entire midline sutural contact (including the horncore) is present, suggesting
 that sutural closure did not occur between the nasals before death (0). In UALVP 40 (Fig.
 10C, D) and AMNH 5402 (Fig. 10E, F), the nasal sutures are closed adjacent to the nasal
 horncore, but open further posteriorly (0). In AMNH 5401 (Fig. 10G, H) and CMN 2280 (Fig. 10I, J), the nasal sutures are closed along their entire length (1).


(8) Nasal and frontal, degree of sutural closure (Longrich & Field, 2012; character 19):
 (0) – suture open in dorsal view.
 (1) – suture closed in dorsal view.
 These character states are exemplified by AMNH 5401, AMNH 5402, CMN
 1254, and UALVP 40 (0), and CMN 2280 (1) (Fig. 10). In CMN 1254 (Fig. 10A, B), the
 nasal and frontal are disarticulated (0). In UALVP 40 (Fig. 10C, D), AMNH 5402 (Fig.
 10E, F) and AMNH 5401 (Fig. 10G, H), the nasal and frontal contact each other, but their
 sutures are still open in dorsal view (0). In CMN 2280 (Fig. 10I, J), the suture between the nasal and frontal is closed in dorsal view (1).


(9) Frontals, degree of sutural closure (Longrich & Field, 2012; character 14):
 (0) – suture open in dorsal view.
 (1) – suture closed in dorsal view.
 These character states are exemplified by AMNH 5401, AMNH 5402, CMN
 1254, and UALVP 40 (0), and CMN 2280 (1) (Fig. 10). On the preserved right frontal of
 CMN 1254 (Fig. 10A, B), the entire midline sutural contact for its unpreserved
 counterpart is present, indicating that sutural closure did not occur between these
 elements before death (0). In UALVP 40 (Fig. 10C, D), AMNH 5402 (Fig. 10E, F) and
 AMNH 5401 (Fig. 10G, H), the frontals contact each other, but their sutural contact is
 still open in dorsal view (0). In CMN 2280 (Fig. 10I, J), the contact between the frontals
 is closed in dorsal view (1).


(10) Frontal and postorbital, degree of sutural closure (Longrich & Field, 2012; character 17):
 (0) – suture open in dorsal view.
 (1) – sutures closed in dorsal view.
 These character states are exemplified by AMNH 5401, AMNH 5402, CMN
 1254, and UALVP 40 (0), and CMN 2280 (1) (Fig. 10). In CMN 1254 (Fig. 10A, B),
 UALVP 40 (Fig. 10C, D), AMNH 5402 (Fig. 10E, F), and AMNH 5401 (Fig. 10G, H),
 the suture between the frontal and postorbital is open in dorsal view (0). In CMN 2280
 (Fig. 10I, J), the contact between the frontal and postorbital is closed in dorsal view (1).

(11) Epijugal, articulation with jugal/quadratojugal (Longrich & Field, 2012; character 22,
 modified here):
 (0) – epijugal disarticulated.
 (1) – articulated, with suture open in lateral view.
 (2) – articulated, with suture closed in lateral view.
 The epijugal is a disarticulated element (0) early in ontogeny, but later articulates
 with the distal ends of the jugal and quadratojugal, retaining open sutures (1); these
 sutures close over time (2). These character states are exemplified by UALVP 40 (0),
 ROM 839 (1) and TMP 1981.019.0175 (2) (Fig. 15). In UALVP 40 (Fig. 15A), the
 epijugal is disarticulated on the right side (0), but articulated with an open suture on the
 opposite side (1; not shown), resulting in a dimorphic state (0&1). In ROM 839 (Fig.
 15B), the epijugal is articulated with an open suture on the right side (1), but
 disarticulated and missing on the opposite side (0; not shown). In TMP 1981.019.0175
 (Fig. 15C), the epijugal is articulated with a closed suture (2).


(12) Squamosal, length/width ratio (Longrich & Field, 2012; character 5 modified and
 quantified):
 (0) – < 2.4.
 (1) – 2.5 to 2.95.
 (2) – > 3.0.
 The squamosal length is defined as the straight distance between the jugal notch
 and the distal end of the squamosal (parameter 19, Fig. 2). The squamosal width is
 defined as the minimum distance between the jugal notch and the medial margin of the
 squamosal (curved measurement, along the surface; parameter 14, Fig. 2). Scannella &
 Horner (2010) noted that the squamosal length/width ratio increases with skull size in
 *Triceratops*, although they defined squamosal width as the maximum width of the
 element. It is unclear whether Scannella & Horner’s (2010) definition of squamosal
 width includes episquamosals; the presence/absence or degree of remodeling of
 episquamosals would affect the maximum squamosal width. Here, the jugal notch was
 chosen as the lateral extent of the squamosal, as it is not affected by the articulation and
 subsequent remodeling of episquamosals.
 The shape of the squamosal is variable amongst *Chasmosaurus* specimens, with
 the length/ratio of this element generally increasing with skull size (Figs 3, 9). The
 above character states are exemplified by CMN 1254 and TMP 1998.128.0001 (0),
 AMNH 5401 and AMNH 5656 (1), and CMN 8800 and NHMUK R4948 (2) (Fig. 9). In
 TMP 1998.128.0001 (Fig. 9A) and CMN 1254 (Fig. 9B), the squamosal has a
 length/width ratio of less than 2.4 (0). In AMNH 5656 (Fig. 9C) and AMNH 5401 (Fig.
 9D), the squamosal has a length/width ratio between 2.5 and 2.95 (1). In NHMUK
 R4948 (Fig. 9E) and CMN 8800 (Fig. 9F), the squamosal has a length/width ratio of
 greater than 3.0 (2).


(13) Squamosals, scalloping of lateral margins (Longrich & Field, 2012; character 6, modified):
 (0) – strongly scalloped.
 (1) – weakly scalloped or unscalloped in dorsal view.
 The scalloping refers to the undulations on the lateral margin of the squamosal,
 which indicate the position of episquamosal attachment sites or loci. These undulations
 are generally less pronounced in relatively large skulls (Fig. 9). These character states
 are exemplified by AMNH 5401, AMNH 5656, CMN 1254, and TMP 1998.128.0001
 (0), and CMN 8800 and NHMUK R4948 (1) (Fig. 9). In TMP 1998.128.0001 (Fig.
 9A), CMN 1254 (Fig. 9B), AMNH 5656 (Fig. 9C), and AMNH 5401 (Fig. 9D) the
 lateral margin of the squamosal is strongly scalloped and undulatory (0). In NHMUK
 R4948 (Fig. 9E) and CMN 8800 (Fig. 9F), the lateral margin of the squamosal is only
 weakly scalloped (1).


(14) Episquamosals, articulation with squamosal (Longrich & Field, 2012; character 20,
 modified):
 (0) – no episquamosals articulated.
 (1) – at least 50% (but not all) of episquamosals articulated.
 (2) – all episquamosals articulated.
 In early ontogeny, episquamosals are disarticulated elements detached from the
 underlying loci that are located at the apices of the undulations located on the lateral
 margin of the squamosal (0). As the animal matured, these episquamosals and the
 squamosal came into contact in a sequential order, progressing in an anterior to posterior
 direction with increasing skull size (1 and 2; Fig. 9). The presence or absence of
 episquamosals can be difficult to determine as, once they are articulated, they are
 remodeled throughout ontogeny (character 15) from triangular-profile elements to low,
 rounded-profile elements whose sutures with the underlying squamosal can become
 completely obliterated. In almost all *Chasmosaurus* specimens, however, the complete
 suture between the episquamosal and squamosal can be discerned, and are coded as
 'open'.
 Episquamosal remodelling can result in a structure that closely resembles the
 unadorned loci of a smaller, putatively less mature specimen (e.g. compare the
 anteriormost locus of CMN 1254, Fig. 9B, with the anteriormost episquamosal of CMN
 8800, Fig. 9F). Such a structure in a large, putatively more mature specimen is
 interpreted as an episquamosal by comparing the structure with more posterior
 episquamosals in that specimen, whose sutures are more distinct.
 Unadorned episquamosal attachment loci can have wide, shallow grooves on the
 dorsal surface that converge at the apex. These grooves can contribute to a complex
 attachment site for the episquamosal as in centrosaurines (e.g. *P*. *lakustai*; Currie *et al.*,
 2008). The number of episquamosals that have articulated with the squamosal (or have
 undergone remodelling, character 15) cannot be quantified, as episquamosal loci count is
 not consistent between specimens (e.g. compare CMN 1254 (Fig. 9B) = seven, and
 CMN 8800 (Fig. 9F) = 10); however, an estimation of none or less than one-half of the
 episquamosals being articulated can be used to differentiate states 0 and 1.
 These character states are exemplified by CMN 1254 and TMP 1998.128.0001
 (0), AMNH 5401 and AMNH 5656 (1), and NHMUK R4948 and CMN 8800 (2) (Fig.
 9). In both TMP 1998.128.0001 (Fig. 9A) and CMN 1254 (Fig. 9B), no
 episquamosals are articulated with the squamosal (0). In AMNH 5656 (Fig. 9C) and
 AMNH 5401 (Fig. 9D), all but the distalmost episquamosal have articulated with the
 squamosal (1). In NHMUK R4948 (Fig. 9E) and CMN 8800 (Fig. 9F), all
 episquamosals have articulated with the squamosal (2); however, the distalmost
 episquamosal is missing on the left squamosal of NHMUK R4948 (1; not shown),
 resulting in a dimorphic state (0&1) for this specimen. Although the episquamosals
 suggest an anterior-to-posterior direction of articulation in specimens, there is a specimen
 (ROM 839) which is missing episquamosal 5 and 6 (1).


(15) Episquamosals, shape (new character):
 (0) – all episquamosals triangular in dorsal profile (i.e. unmodified).
 (1) – anteriormost (approximately one-half of total) D-shaped in dorsal profile,
 posteriormost unmodified.
 (2) – all episquamosals D-shaped in dorsal profile.
 After articulation of the triangular episquamosals (0), they underwent remodeling
 from anterior to posterior along the margin of the squamosal into a low, "D"-shape on the
 anterior half (1), and finally the entire margin (2) with increasing skull size (Fig. 9).
 These character states are exemplified by AMNH 5656 (Fig. 9C) and AMNH 5401 (Fig.
 9D) (0), NHMUK R4948 (1; Fig. 9E), and CMN 8800 (2; Fig. 9F).
 Horner & Goodwin (2006) demonstrated that during *Triceratops* ontogeny the
 episquamosals are laterally compressed, with an approximately triangular profile in
 dorsal view in the smallest known specimens (e.g. MOR 1199, UCMP 136306), but
 become reduced in height proximodistally during maturity, finally completely articulating
 and being obliterated in putatively mature specimens (e.g. MOR 004, MOR 1120).


(16) Parietal, lateral bar (new character):
 (0) – continuous, completely excluding squamosal from parietal fenestra.
 (1) – discontinuous, allowing squamosal to form part of margin of parietal fenestra.
 These character states are exemplified by AMNH 5656 and CMN 1254 (0), and
 CMN 2280 (1) (Fig. 12). In CMN 2280 (Fig. 12D), the lateral parietal bar tapers medially
 and becomes discontinuous (1). The continuity of the lateral parietal bar was previously
 considered to be a diagnostic character within *Chasmosaurus*, being continuous in *C*.
 *belli* and discontinuous in *C*. *russelli* (Godfrey & Holmes, 1995). However, exceptions
 are known, e.g. AMNH 5656 (*C*. *russelli*) has continuous bars, and YPM 2016 (*C*. *belli*)
 has discontinuous bars. This feature, therefore, does not represent a robust diagnostic
 feature separating these two species. Instead, this feature appears to be size-related, with
 a continuous bar occurring in all small and some large-sized *Chasmosaurus* skulls, and a
 discontinuous bar occurring in most large skulls (Figs 3, 12). This suggests that the lateral
 bar becomes narrow, and finally discontinuous, with maturity.
 The continuity of the lateral parietal bar also varies in the holotype of *V*.
 *irvinensis* (CMN 41357), with only the left side being continuous. Lehman (1989) noted
 that the continuity of the lateral parietal bar varies with age, and occasionally between
 sides of the frill in the same individual of *Agujaceratops mariscalensis* and
 *Pentaceratops sternbergi*. It is difficult to assess whether such individuals are
 consistently more mature than individuals with complete bars due to the small sample
 sizes within each taxon (in the case of *V*. *irvinensis*), or insufficient discussion and
 figuring of specimens with the asymmetric condition in the literature (in the case of *A*.
 *mariscalensis* and *P*. *sternbergi*, e.g. Lehman, 1989; Forster *et al.*, 1993; Lehman, 1998).


(17) Epiparietals, articulation with parietal (Longrich & Field, 2012; character 21, modified):
 (0) – no epiparietals articulated.
 (1) – at least one (but not all) epiparietal articulated.
 (2) – all epiparietals articulated.
 Epiparietals, like episquamosals, are inferred to have a dermal origin and
 sequentially articulate with the underlying parietal. Almost every skull previously
 referred to *Chasmosaurus* has, or are assumed to have, three epiparietal loci on each side
 of the posterior bar, for a total of six (Fig. 3). Of these skulls, loci are unadorned (i.e.
 CMN 1254, Fig. 12A), partly adorned (i.e. one, CMN 2245, Fig. 3I; two, AMNH 5656,
 Fig. 3E), or completely adorned (Fig. 3A, C, F–H) with epiparietals. Asymmetric
 epiparietal articulation also occurs, as the left P2 locus of CMN 8803 is adorned, but its
 right counterpart is not (Fig. 3B).
 As no known specimens express (0) this state is assigned to the artificial
 embryonic stage. The remaining character states are exemplified by AMNH 5656 (1), and
 CMN 2280 and TMP 1983.025.0001 (2) (Fig. 12). The medialmost epiparietals (P1 and
 2) are articulated in AMNH 5656 (Fig. 12B), but the lateralmost epiparietal loci (P3) is
 unoccupied (1). In TMP 1983.025.0001 (Fig. 12C) and CMN 2280 (Fig. 12D), all
 epiparietals have articulated with the parietal (2). In ROM 843, the low-relief mound
 present at the medialmost epiparietal loci (P1) is interpreted as a modified epiparietal;
 therefore, all three epiparietals are interpreted as articulated, and this specimen was coded
 as (2).


(18) Medial epiparietals/underlying parietal margin, orientation (new character):
 (0) – in plane of frill.
 (1) – anterodorsally recurved.
 The orientation of the medialmost epiparietals is also variable amongst
 *Chasmosaurus* skulls. About half of these specimens have epiparietal loci that are
 oriented in the plane of the frill (e.g. TMP 1983.025.0001, Fig. 3G), and are oriented
 anterodorsally in all other specimens (e.g. CMN 2280, Fig. 12D). Amongst specimens
 with the latter condition, the P2 is either oriented in the plane of the frill (i.e. ROM 843,
 Fig. 3H) or anterodorsally (all other specimens). The anterodorsal orientation of
 epiparietals is due to an anterodorsally-thickened ridge on the posterior parietal margin,
 which is interrupted medially in specimens with three epiparietals per side (e.g. CMN
 2280), but spans the entire margin in specimens with five epiparietals per side (i.e. YPM
 2016, Fig. 3M). The ridge in CMN 0491 (Fig. 3J) conforms to the former pattern, and
 NHMUK R4948 (Fig. 3K) to the latter, although the number of epiparietals cannot be
 determined reliably for either specimen.
 The above character states are exemplified by AMNH 5656 and TMP
 1983.025.0001 (0), and CMN 2280 (1) (Fig. 12). In CMN 2280 (Fig. 12D), the two
 medial epiparietals (P1 and 2) are slightly anterodorsally recurved. In small (AMNH 5656) and some large-sized *Chasmosaurus* skulls (AMNH 5402 and TMP
 1983.025.0001), epiparietals are oriented in the plane of the frill. However, in nearly all
 large skulls, epiparietals are oriented slightly anterodorsally.

Table A. Character-specimen matrix used for ontogenetic analysis**.**

Specimen 5 10 15 18

Embryo 00000 00000 0000? 000
CMN 1254 ?000? 00000 ?000? 0??
AMNH 5656 ????? ????? 01010 010
UALVP 40 01000 00000 (01)10?0 ???
AMNH 5401 11000 01000 11010 ???
AMNH 5402 12000 00000 22111 020
CMN 2245 ?100? ??011 020(12)1 011
ROM 839 ?2000 01110 (01)2011 0??
NHMUK R4948 12011 01??0 221(12)1 ??1
ROM 843 ?2021 01111 22121 021
TMP 1981.019.0175 220(12)1 11000 201?1 1??
TMP 1983.025.0001 ???1? ????? 21121 120
YPM 2016 22121 11110 21121 121
CMN 2280 221(01)1 11111 21121 121
CMN 8800 ?21(12)1 11111 ?2122 121
