# Supplementary material for: A Re-Evaluation of the Chasmosaurine Ceratopsid Genus Chasmosaurus (Dinosauria: Ornithischia) from the Upper Cretaceous (Campanian) Dinosaur Park Formation of Western Canada
Source: PLoS One. 2016 Jan 4;11(1):e0145805. doi: 10.1371/journal.pone.0145805 (PMC4699738; doi:10.1371/journal.pone.0145805)
Supplement: S10 File — (DOC) [file pone.0145805.s010.doc]

**Supplementary Material 10. Supporting data for cranial morphometric analyses.**

Table A: Measurement dataset used in PCAs 1 and 2; 18 specimens of *Chasmosaurus russelli* (n = 4), *Chasmosaurus* *belli* (n = 5), *Chasmosaurus* sp. (n = 7), and *Vagaceratops irvinensis* (n = 2). Estimated missing data shown in bold. All parameter measurements are in mm.

| Specimen/Parameter | 1 | 4 | 5 | 16 | 18 | 19 | 22 | 27 | 28 | 30 | 31 | 32 |
| --- | --- | --- | --- | --- | --- | --- | --- | --- | --- | --- | --- | --- |
| AMNH 5401 | 370 | 320 | 130 | 730 | 315 | 710 | 115 | 820 | 875 | **678** | **34** | **91** |
| AMNH 5402 | 70 | 190 | 144 | 680 | 250 | 685 | 120 | 920 | 780 | 354 | **33** | **84** |
| AMNH 5656 | **150** | **272** | **156** | **763** | **266** | 569 | 129 | 697 | 854 | 503 | 27 | 86 |
| CMN 1254 | 216 | 242 | 150 | **773** | **264** | 500 | **126** | **990** | **949** | **546** | **33** | **89** |
| CMN 2245 | 102 | 238 | 176 | **753** | 269 | 779 | 140 | 1078 | 988 | 452 | 32 | 116 |
| CMN 2280 | 132 | 263 | **155** | 750 | 278 | 709 | 132 | 1043 | 855 | 557 | 34 | 72 |
| CMN 8800 | **207** | 412 | **158** | 880 | 309 | 997 | **123** | 848 | 1143 | 584 | 36 | 79 |
| CMN 8801 | **152** | 300 | 194 | 835 | **266** | **714** | **134** | **996** | **919** | **486** | **33** | **88** |
| CMN 34829 | 59 | 244 | **150** | **736** | 299 | 850 | **164** | **1017** | **824** | **327** | 40 | **83** |
| CMN 41357 | **63** | 250 | 123 | 795 | 320 | 700 | 225 | 1047 | 753 | 265 | 33 | 76 |
| NHMUK R4948 | **182** | **283** | 155 | **771** | **265** | 840 | **128** | 1160 | **942** | 627 | **33** | 90 |
| ROM 839 | 107 | 250 | 237 | 782 | 231 | 917 | 115 | 893 | 1034 | **431** | 26 | **86** |
| ROM 843 | **143** | 252 | 199 | 897 | 290 | 893 | 151 | 1227 | 1063 | 498 | 30 | 94 |
| TMP 1981.019.0175 | **122** | 302 | 134 | 768 | 217 | 600 | 171 | **1002** | **891** | **435** | **33** | **86** |
| TMP 1983.025.0001 | **192** | 345 | **158** | **773** | **264** | 650 | 125 | 1373 | 952 | 592 | 45 | 102 |
| TMP 1987.045.0001 | **71** | 233 | 124 | 617 | 285 | 633 | 157 | 940 | 755 | 260 | **33** | **83** |
| UALVP 40 | 176 | 240 | 131 | 628 | 180 | 600 | 88 | **992** | **937** | **521** | **33** | **88** |
| YPM 2016 | **106** | 222 | **153** | 800 | 271 | 730 | 193 | 1162 | 840 | 463 | 34 | 75 |

Table B: Results of PCA 1 – standard deviation, variance and cumulative variance, and parameter loadings on principal component (PC) axes.

| Axis | PC1 | PC2 | PC3 | PC4 | PC5 | PC6 | PC7 | PC8 | PC9 | PC410 | PC11 | PC12 |
| --- | --- | --- | --- | --- | --- | --- | --- | --- | --- | --- | --- | --- |
| Standard deviation | 1.9699 | 1.5566 | 1.3514 | 1.2171 | 0.84399 | 0.83510 | 0.65531 | 0.49698 | 0.36525 | 0.31766 | 0.2019 | 0.16653 |
| Variance (%) | 32.34 | 20.19 | 15.22 | 12.34 | 5.936 | 5.812 | 3.579 | 2.058 | 1.112 | 0.841 | 0.34 | 0.231 |
| Cumulative variance (%) | 32.34 | 52.53 | 67.75 | 80.09 | 86.028 | 91.840 | 95.418 | 97.476 | 98.588 | 99.429 | 99.77 | 100 |
| Parameter 1 | -0.3982 | -0.2378 | 0.2395 | -0.1980 | 0.2128 | -0.0007 | 0.2327 | -0.2025 | 0.0670 | -0.2207 | -0.0150 | 0.7032 |
| 4 | -0.3413 | 0.0959 | 0.3528 | -0.2641 | -0.1333 | -0.0391 | -0.5825 | 0.0544 | 0.4552 | -0.2400 | 0.0603 | -0.2241 |
| 5 | -0.2907 | 0.2006 | -0.4730 | 0.1771 | 0.0416 | 0.0477 | 0.1014 | 0.6089 | 0.3358 | -0.1246 | -0.2812 | 0.1664 |
| 16 | -0.2907 | 0.4462 | -0.0381 | -0.1087 | 0.3963 | 0.1481 | -0.1104 | 0.1466 | -0.2557 | 0.3123 | 0.5690 | 0.0622 |
| 18 | 0.0221 | 0.4437 | 0.2267 | -0.3009 | 0.0107 | -0.5054 | 0.4767 | 0.1263 | -0.1320 | -0.3106 | -0.0607 | -0.2107 |
| 19 | -0.1867 | 0.4234 | -0.1986 | 0.0147 | -0.6415 | -0.0107 | 0.1340 | -0.4312 | 0.1771 | 0.2361 | 0.0776 | 0.1937 |
| 22 | 0.2708 | 0.4578 | 0.1031 | -0.0072 | 0.4184 | 0.0092 | -0.2446 | -0.2684 | 0.1334 | 0.1967 | -0.5555 | 0.1853 |
| 27 | -0.0352 | 0.2216 | 0.2317 | 0.6607 | 0.1553 | 0.2971 | 0.2036 | -0.2211 | 0.1696 | -0.4128 | 0.1810 | -0.1576 |
| 28 | -0.4620 | 0.0566 | -0.1453 | 0.0999 | -0.0833 | 0.0574 | -0.2479 | -0.1656 | -0.6562 | -0.2581 | -0.3723 | -0.1316 |
| 30 | -0.4341 | -0.1739 | 0.1626 | -0.0688 | 0.1537 | 0.1730 | 0.3882 | -0.0935 | 0.1756 | 0.4748 | -0.2723 | -0.4546 |
| 31 | 0.0034 | 0.0834 | 0.6239 | 0.2880 | -0.3373 | 0.0652 | -0.0226 | 0.4417 | -0.2198 | 0.2811 | -0.1466 | 0.2416 |
| 32 | -0.2102 | -0.1403 | -0.0317 | 0.4721 | 0.1519 | -0.7700 | -0.1579 | -0.0980 | 0.0715 | 0.2196 | 0.0896 | 0.0186 |

Table C: Results of PCA 2 – standard deviation, variance and cumulative variance, and parameter loadings on principal component (PC) axes.

| Axis | PC1 | PC2 | PC3 | PC4 | PC5 | PC6 | PC7 | PC8 | PC9 | PC10 | PC11 | PC12 |
| --- | --- | --- | --- | --- | --- | --- | --- | --- | --- | --- | --- | --- |
| Standard deviation | 0.5744 | 0.2271 | 0.18245 | 0.17499 | 0.1203 | 0.11079 | 0.09445 | 0.07438 | 0.06797 | 0.03618 | 0.03287 | 0.02127 |
| Variance (%) | 66.78 | 10.44 | 6.737 | 6.198 | 2.93 | 2.484 | 1.805 | 1.120 | 0.935 | 0.265 | 0.219 | 0.092 |
| Cumulative variance (%) | 66.78 | 77.22 | 83.953 | 90.150 | 93.08 | 95.565 | 97.370 | 98.490 | 99.425 | 99.690 | 99.908 | 100 |
| Parameter 1 | -0.8303 | 0.2142 | -0.0033 | 0.0949 | -0.2322 | 0.0872 | -0.0741 | 0.1888 | -0.3056 | 0.1332 | -0.2003 | 0.0413 |
| 4 | -0.1798 | 0.2358 | 0.4685 | -0.1083 | 0.5167 | 0.4075 | -0.1996 | -0.2758 | 0.0462 | -0.3546 | 0.0354 | 0.0528 |
| 5 | -0.0129 | -0.5440 | 0.0688 | -0.0242 | -0.2514 | 0.2839 | -0.0592 | -0.6310 | -0.2874 | 0.2012 | -0.0969 | 0.1433 |
| 16 | -0.0137 | 0.0253 | 0.0916 | 0.0279 | -0.0826 | 0.0950 | -0.1158 | -0.1073 | -0.0148 | 0.1379 | 0.0908 | -0.9601 |
| 18 | 0.0430 | 0.3016 | 0.4143 | 0.0433 | -0.4567 | -0.0703 | 0.5653 | -0.1722 | -0.1175 | -0.1929 | 0.3442 | 0.0381 |
| 19 | 0.0448 | -0.2905 | 0.6955 | -0.2864 | -0.1725 | -0.2914 | -0.2075 | 0.3183 | 0.0829 | 0.0571 | -0.2807 | 0.0056 |
| 22 | 0.2655 | 0.5220 | 0.0326 | 0.0086 | -0.3971 | 0.1995 | -0.4994 | -0.1143 | 0.2313 | 0.3300 | -0.0103 | 0.1830 |
| 27 | 0.0414 | 0.0727 | -0.2876 | -0.7473 | -0.2052 | -0.0109 | -0.2026 | 0.0268 | -0.3254 | -0.3797 | 0.1244 | -0.0476 |
| 28 | -0.1052 | -0.2233 | 0.0993 | -0.1042 | 0.0996 | 0.2154 | -0.1001 | 0.3152 | -0.0358 | 0.3623 | 0.7786 | 0.1179 |
| 30 | -0.4328 | -0.1008 | -0.1006 | -0.1570 | -0.0907 | -0.3957 | -0.0945 | -0.3802 | 0.6285 | -0.0643 | 0.2240 | 0.0141 |
| 31 | -0.0122 | 0.2626 | 0.0224 | -0.4899 | 0.3286 | -0.1279 | 0.3696 | -0.1848 | -0.0544 | 0.6047 | -0.1633 | 0.0024 |
| 32 | -0.0577 | -0.1409 | -0.0885 | -0.2449 | -0.2117 | 0.6226 | 0.3593 | 0.2361 | 0.4923 | -0.0489 | -0.2124 | -0.0431 |
